# Supplementary material for: Nickel-catalyzed acylzincation of allenes with organozincs and CO
Source: Nat Commun. 2023 Oct 31;14:6960. doi: 10.1038/s41467-023-42716-2 (PMC10618444; doi:10.1038/s41467-023-42716-2)
Supplement: Supplementary file 1 — Supplementary Information [file 41467_2023_42716_MOESM1_ESM.pdf]

Supplementary Information

Nickel-Catalyzed Acylzincation of Allenes with Organozincs and CO

Xianqing Wu[1], Chenglong Wang[1], Ning Liu[1], Jingping Qu[1] and Yifeng Chen[1]*

[1]Key Laboratory for Advanced Materials and Joint International Research Laboratory of Precision Chemistry and Molecular Engineering, Feringa Nobel Prize Scientist Joint Research Center, Frontiers Science Center for Materiobiology and Dynamic Chemistry, School of Chemistry and Molecular Engineering, East China University of Science and Technology, 130 Meilong Road, Shanghai, 200237 (China)

E-mail: yifengchen@ecust.edu.cn

Table of Contents

General information 3

Experimental procedures and characterization data for substrates 4

Experimental procedures and characterization data for products 8

Synthetic application 16

Mechanistic studies 21

NMR spectra 26

References 73

General information

All reaction with CO gas was carried out in fume hood with the CO detector. All catalytic reactions were carried out under 1 atm of CO and anhydrous conditions unless otherwise indicated. All raw materials synthesis were carried out under nitrogen atmosphere and anhydrous conditions unless otherwise indicated. Unless otherwise noted, all catalytic reactions were run in dried glassware. THF was distilled from sodium/benzophenone. DMA (CAS 121-69-7) was purchased from Adamas (99.8%, SafeDry, Water ≤ 50 ppm (by K.F.), SafeSeal). $NiBr_2 \cdot DME$ (CAS 28923-39-9) was purchased from Sinocompound. Reactions were monitored by thin-layer chromatography (TLC) carried out on 0.20 mm Huanghai silica gel plates (HSGF 254) using UV light as the visualizing agent and an acidic solution of phosphomolybdic acid (PMA) with heat as the stains. All new compounds were characterized by means of ^{1}H NMR, ^{13}C NMR, ^{19}F NMR and HRMS. NMR spectra were recorded using a Bruker AVANCE III 400 MHz NMR spectrometer and can be found at the end of the paper. High-resolution mass spectra (HRMS) were recorded on a Q Exactive plus 4G mass spectrometer using ESI-Quadrupole-Orbitrap LC-MS. All ^{1}H-NMR data are reported in δ units, parts per million (ppm), and were calibrated relative to the signals for residual chloroform (7.26 ppm) in deuterochloroform ($CDCl_3$). All ^{13}C-NMR data are reported in ppm relative to $CDCl_3$ (77.16 ppm) and were obtained with ^{1}H decoupling. The following abbreviations or combinations thereof were used to explain the multiplicities: s = singlet, d = doublet, t = triplet, q = quartet, quint = quintet, sext = sextet, bs = broad singlet, m = multiplet.

Experimental procedures and characterization data for substrates

Compounds **1a**[1] were synthesized according to the published procedures.

General procedure A for allenes synthesis

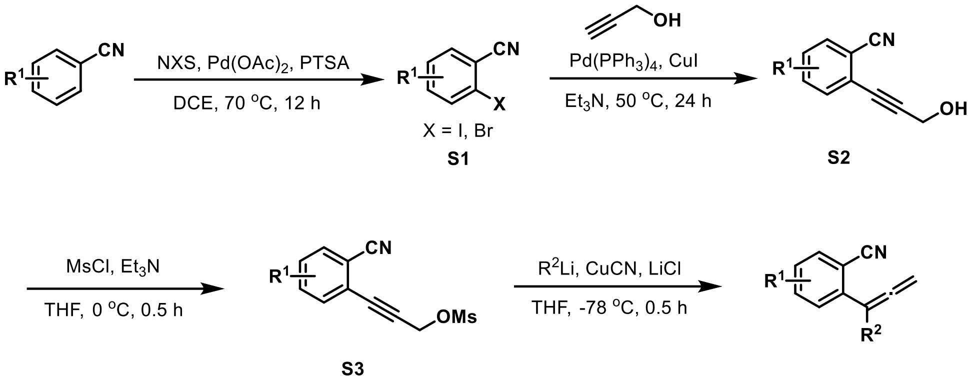

Arylnitrile (1.0 equiv), NXS (1.1 equiv), $Pd(OAc)_2$ (0.05 equiv), PTSA (0.5 equiv), and DCE (0.5 M) were added into a schlenk tube, and the mixture was stirred for 12 h at 70 ℃. After cooling to room temperature, the reaction was quenched with H_2O, extracted with DCM for three times, washed with brine, dried over Na_2SO_4 and then purified by flash column chromatography to afford **S1**.

To a stirred suspension of $Pd(PPh_3)_4$ (0.03 equiv), CuI (0.1 equiv) and 2-Bromobenzonitrile (1.0 equiv) in Et_3N was added propargyl alcohol (1.5 equiv), and the resulted mixture was heated at 50 ℃ for 24 h. When the starting material was completely converted (monitored by TLC), the reaction was quenched with H_2O, extracted with EtOAc for three times, washed with brine, dried over Na_2SO_4 and then purified by flash column chromatography to afford **S2**.

To a solution of **S2** (1.0 equiv) and Et_3N (2.5 equiv) in THF was added MsCl (2.0 equiv) dropwise at 0 °C. After stirring for 0.5 h at same temperature, the reaction was quenched by addition of saturated $NaHCO_3$, extracted with EtOAc, The extract was washed with water and brine, dried over Na_2SO_4 and then concentrated to dryness. The crude **S3** was carried through to the next step;

To a solution of CuCN (4.0 equiv) and anhydrous LiCl (8.0 equiv) in THF was added RLi (4.0 equiv, 1.0 M in Et_2O) at -78 ℃, and the mixture was stirred for 5 min at -30 ℃. Then, the crude **S3** (1.0 equiv) in THF was added to the mixture at -78 ℃. After stirring for 0.5 h at same temperature, the reaction was quenched by addition of saturated aqueous NH_4Cl, extracted with EtOAc for three times, washed with brine, dried over Na_2SO_4 and then purified by flash column chromatography to afford corresponding allenes.

2-(buta-2,3-dien-2-yl)-4-methylbenzonitrile (1b)

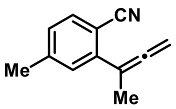

General procedure was followed from **S1** on 2 mmol scale. The reaction mixture was purified by flash column chromatography (PE/EtOAc = 20/1) to afford **1b** as a colorless oil (177.9 mg, 55%)，R_f = 0.51 (PE/EtOAc = 20/1); **^{1}H NMR** (400 MHz, $CDCl_3$): δ 7.52 (d, J = 8.0 Hz, 1H), 7.21 (s, 1H), 7.11 (dd, J = 8.0, 0.8 Hz, 1H), 5.03 (q, J = 3.2 Hz, 2H), 2.40 (s, 3H), 2.13 (t, J = 3.2 Hz, 3H); **^{13}C NMR** (100 MHz, $CDCl_3$): δ 208.3, 143.5, 141.8, 133.9, 128.4, 128.1, 118.6, 108.4, 98.0, 77.9, 21.9, 18.8; **HRMS** (ESI): $[M+H]^+$ Calcd for $C_{12}H_{12}N$: 170.0964; found: 170.0961.

2-(buta-2,3-dien-2-yl)-5-methylbenzonitrile (1c)

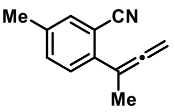

General procedure was followed from benzonitrile on 5 mmol scale. The reaction mixture was purified by flash column chromatography (PE/EtOAc = 20/1) to afford **1c** as a colorless oil (234.2 mg, 28%)，R_f = 0.55 (PE/EtOAc = 20/1); **^{1}H NMR** (400 MHz, $CDCl_3$): δ 7.44 (s, 1H), 7.35 (dd, *J* = 8.0, 1.2 Hz, 1H), 7.30 (d, *J* = 8.0 Hz, 1H), 5.03 (q, *J* = 3.2 Hz, 2H), 2.36 (s, 3H), 2.12 (t, *J* = 3.2 Hz, 3H); **^{13}C NMR** (100 MHz, $CDCl_3$): δ 208.2, 139.0, 137.4, 134.3, 133.6, 127.6, 118.5, 111.2, 97.8, 78.0, 20.8, 18.8; **HRMS** (ESI): $[M+H]^+$ Calcd for $C_{12}H_{12}N$: 170.0964; found: 170.0959.

2-(buta-2,3-dien-2-yl)-4,5-dimethylbenzonitrile (1d)

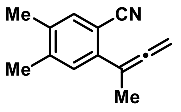

General procedure was followed from benzonitrile on 5 mmol scale. The reaction mixture was purified by flash column chromatography (PE/EtOAc = 20/1) to afford **1d** as a colorless oil (309.4 mg, 34%)，R_f = 0.64 (PE/EtOAc = 20/1); **^{1}H NMR** (400 MHz, $CDCl_3$): δ 7.39 (s, 1H), 7.16 (s, 1H), 5.01 (q, *J* = 3.2 Hz, 2H), 2.31 (s, 3H), 2.26 (s, 3H), 2.12 (t, *J* = 3.2 Hz, 3H); **^{13}C NMR** (100 MHz, $CDCl_3$): δ 208.2, 142.3, 139.3, 136.2, 134.6, 128.9, 118.7, 108.5, 97.9, 77.7, 20.3, 19.3, 18.9; **HRMS** (ESI): $[M+H]^+$ Calcd for $C_{13}H_{14}N$: 184.1121; found: 184.1123.

3-(buta-2,3-dien-2-yl)-[1,1'-biphenyl]-4-carbonitrile (1e)

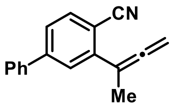

General procedure was followed from benzonitrile on 7 mmol scale. The reaction mixture was purified by flash column chromatography (PE/EtOAc = 20/1) to afford **1e** as a colorless oil (212.6 mg, 62%)，R_f = 0.60 (PE/EtOAc = 20/1); **^{1}H NMR** (400 MHz, $CDCl_3$): δ 7.71 (d, *J* = 8.0 Hz, 1H), 7.62–7.58 (m, 3H), 7.53 (dd, *J* = 8.4, 2.0 Hz, 1H), 7.51–7.47 (m, 2H), 7.45–7.41 (m, 1H), 5.09 (q, *J* = 3.2 Hz, 2H), 2.21 (t, *J* = 3.2 Hz, 3H); **^{13}C NMR** (100 MHz, $CDCl_3$): δ 208.4, 145.6, 142.4, 139.5, 134.5, 129.2, 128.7, 127.4, 126.4, 126.0, 118.4, 110.0, 98.0, 78.3, 18.8; **HRMS** (ESI): $[M+H]^+$ Calcd for $C_{17}H_{14}N$: 232.1121; found: 232.1115.

2-(buta-2,3-dien-2-yl)-4-methoxybenzonitrile (1f)

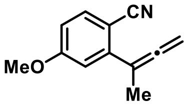

General procedure was followed from **S1** on 2 mmol scale. The reaction mixture was purified by flash column chromatography (PE/EtOAc = 20/1) to afford **1f** as a colorless oil (283.1 mg, 77%)，R_f = 0.49 (PE/EtOAc = 20/1); **^{1}H NMR** (400 MHz, $CDCl_3$): δ 7.53 (d, *J* = 8.8 Hz, 1H), 6.87 (d, *J* = 2.8 Hz, 1H), 6.79 (dd, *J* = 8.8, 2.4 Hz, 1H), 5.02 (q, *J* = 3.2 Hz, 2H), 3.83 (s, 3H), 2.11 (t, *J* = 3.2 Hz, 3H); **^{13}C NMR** (100 MHz, $CDCl_3$): δ 208.2, 162.7, 143.8, 135.6, 118.6, 113.4, 112.8, 103.1, 97.9, 78.0, 55.6, 18.6; **HRMS** (ESI): $[M+H]^+$ Calcd for $C_{12}H_{12}NO$: 186.0913; found: 186.0910.

2-(buta-2,3-dien-2-yl)-4,5-dimethoxybenzonitrile (1g)

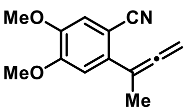

General procedure was followed from benzonitrile on 5 mmol scale. The reaction mixture was purified by flash column chromatography (PE/EtOAc = 20/1) to afford **1g** as a colorless oil (390.0 mg, 36%)，R_f = 0.37 (PE/EtOAc = 20/1); **^{1}H NMR** (400 MHz, $CDCl_3$): δ 7.04 (s, 1H), 6.83 (s, 1H), 5.02 (q, *J* = 3.2 Hz, 2H), 3.93 (s, 3H), 3.89 (s, 3H), 2.13 (t, *J* = 3.2

Hz, 3H); ^{13}**C NMR** (100 MHz, $CDCl_3$): δ 208.1, 152.5, 148.0, 136.5, 118.7, 115.1, 110.1, 102.6, 97.9, 77.7, 56.3, 56.2, 18.9; **HRMS** (ESI): $[M+H]^+$ Calcd for $C_{13}H_{14}NO_2$: 216.1019; found: 216.1022.

6-(buta-2,3-dien-2-yl)benzo[d][1,3]dioxole-5-carbonitrile (1h)

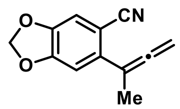

General procedure was followed from benzonitrile on 5 mmol scale. The reaction mixture was purified by flash column chromatography (PE/EtOAc = 20/1) to afford **1h** as a colorless oil (364.5 mg, 37%)，R_f = 0.35 (PE/EtOAc = 20/1); ^{1}**H NMR** (400 MHz, $CDCl_3$): δ 7.00 (s, 1H), 6.84 (s, 1H), 6.05 (s, 2H), 5.00 (q, *J* = 3.2 Hz, 2H), 2.08 (t, *J* = 3.2 Hz, 3H); ^{13}**C NMR** (100 MHz, $CDCl_3$): δ 208.0, 151.6, 146.8, 138.8, 118.3, 112.3, 107.9, 103.8, 102.5, 97.7, 77.8, 19.0; **HRMS** (ESI): $[M+H]^+$ Calcd for $C_{12}H_{10}NO_2$: 200.0706; found: 200.0710.

2-(buta-2,3-dien-2-yl)-4-chlorobenzonitrile (1i)

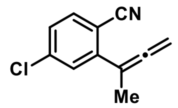

General procedure was followed from **S1** on 2 mmol scale. The reaction mixture was purified by flash column chromatography (PE/EtOAc = 20/1) to afford **1i** as a colorless oil (306.2 mg, 80%)，R_f = 0.49 (PE/EtOAc = 20/1); ^{1}**H NMR** (400 MHz, $CDCl_3$): δ 7.57 (d, *J* = 8.4 Hz, 1H), 7.460 (d, *J* = 2.0 Hz, 1H), 7.29 (dd, *J* = 8.0, 2.0 Hz, 1H), 5.10 (q, *J* = 3.2 Hz, 2H), 2.13 (t, *J* = 3.2 Hz, 3H); ^{13}**C NMR** (100 MHz, $CDCl_3$): δ 208.5, 143.6, 139.3, 135.1, 128.0, 127.6, 117.5, 109.7, 97.3, 78.9, 18.6; **HRMS** (ESI): $[M+H]^+$ Calcd for $C_{11}H_9ClN$: 190.0418; found: 190.0421.

2-(buta-2,3-dien-2-yl)-5-fluorobenzonitrile (1j)

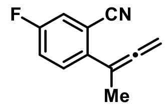

General procedure was followed from **S1** on 2 mmol scale. The reaction mixture was purified by flash column chromatography (PE/EtOAc = 20/1) to afford **1j** as a colorless oil (212.9 mg, 62%)，R_f = 0.59 (PE/EtOAc = 20/1); ^{1}**H NMR** (400 MHz, $CDCl_3$): δ 7.40 (dd, *J* = 8.8, 5.2 Hz, 1H), 7.33 (dd, *J* = 8.0, 2.8 Hz, 1H), 7.26 (ddd, *J* = 8.8, 8.0, 2.8 Hz, 1H), 5.04 (q, *J* = 3.2 Hz, 2H), 2.12 (t, *J* = 3.2 Hz, 3H); ^{13}**C NMR** (100 MHz, $CDCl_3$): δ 208.2, 160.8 (d, $J_{C\text{-}F}$ = 248.2 Hz), 138.3 (d, $J_{C\text{-}F}$ = 3.6 Hz), 129.6 (d, $J_{C\text{-}F}$ = 8.0 Hz), 120.4 (d, $J_{C\text{-}F}$ = 14.8 Hz), 120.3 (d, $J_{C\text{-}F}$ = 11.9 Hz), 117.0 (d, $J_{C\text{-}F}$ = 2.6 Hz), 112.6 (d, $J_{C\text{-}F}$ = 8.9 Hz), 97.1, 78.3, 18.9; ^{19}**F NMR** (376 MHz, $CDCl_3$): -113.4; **HRMS** (ESI): $[M+H]^+$ Calcd for $C_{11}H_9FN$: 174.0714; found: 174.0711.

2-(buta-2,3-dien-2-yl)-6-fluorobenzonitrile (1k)

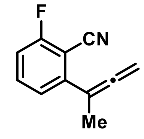

General procedure was followed from **S1** on 2 mmol scale. The reaction mixture was purified by flash column chromatography (PE/EtOAc = 20/1) to afford **1k** as a colorless oil (285.5 mg, 82%)，R_f = 0.53 (PE/EtOAc = 20/1); ^{1}**H NMR** (400 MHz, $CDCl_3$): δ 7.53 (td, *J* = 8.0, 6.0 Hz, 1H), 7.21 (d, *J* = 8.0 Hz, 1H), 7.07 (t, *J* = 8.4 Hz, 1H), 5.09 (q, *J* = 3.2 Hz, 2H), 2.13 (t, *J* = 3.2 Hz, 3H); ^{13}**C NMR** (100 MHz, $CDCl_3$): δ 208.4, 164.2 (d, $J_{C\text{-}F}$ = 256.9 Hz), 143.8, 134.0 (d, $J_{C\text{-}F}$ = 9.2 Hz), 123.1 (d, $J_{C\text{-}F}$ = 3.1 Hz), 114.2 (d, $J_{C\text{-}F}$ = 20.1 Hz), 112.9, 100.6 (d, $J_{C\text{-}F}$ = 15.4 Hz), 97.4 (d, $J_{C\text{-}F}$ = 2.3 Hz), 78.8, 18.6; ^{19}**F NMR** (376 MHz, $CDCl_3$): -105.6; **HRMS** (ESI): $[M+H]^+$ Calcd for $C_{11}H_9FN$: 174.0714; found: 174.0717.

2-(hepta-1,2-dien-3-yl)benzonitrile (1m)

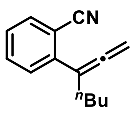

General procedure was followed from **S1** on 5 mmol scale. The reaction mixture was purified by flash column chromatography (PE/EtOAc = 20/1) to afford **1f** as a colorless oil (638.4 mg, 65%)， R_f = 0.61 (PE/EtOAc = 20/1); **^{1}H NMR** (400 MHz, $CDCl_3$): δ 7.65 (d, *J* = 8.0 Hz, 1H), 7.55 (t, *J* = 8.0 Hz, 1H), 7.41 (d, *J* = 8.0 Hz, 1H), 7.32 (t, *J* = 7.6 Hz, 1H), 5.07 (t, *J* = 3.2 Hz, 2H), 2.46–2.41 (m, 2H), 1.50–1.34 (m, 4H), 0.91 (t, *J* = 3.2 Hz, 3H); **^{13}C NMR** (100 MHz, $CDCl_3$): δ 207.6, 142.0, 133.9, 132.6, 128.0, 127.3, 118.3, 112.1, 103.0, 78.8, 31.7, 30.0, 22.3, 14.0; **HRMS** (ESI): $[M+H]^+$ Calcd for $C_{14}H_{16}N$: 198.1277; found: 198.1273.

Procedure for the synthesis of 2-(penta-1,2-dien-3-yl)benzonitrile (1l)

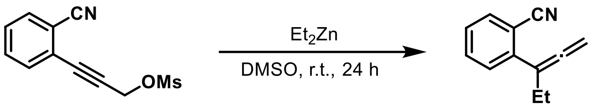

S3

To a solution of **S3**[1] (1.0 equiv) in DMSO was added Et_2Zn (2.0 equiv, 1.0 M in hexane) dropwise at r.t. After stirring for 24 h at same temperature, the reaction was quenched by addition of saturated aqueous NH_4Cl, extracted with EtOAc, The extract was washed with water and brine, dried over Na_2SO_4 and then purified by flash column chromatography to afford **1l** (73.0 mg, 54%); R_f = 0.51 (PE/EtOAc = 20/1). **^{1}H NMR** (400 MHz, $CDCl_3$): δ 7.65 (dd, *J* = 8.0, 0.8 Hz, 1H), 7.55 (td, *J* = 8.0, 1.2 Hz, 1H), 7.41 (d, *J* = 7.6 Hz, 1H), 7.32 (td, *J* = 7.6, 0.8 Hz, 1H), 5.11 (t, *J* = 3.2 Hz, 2H), 2.49–2.41 (m, 2H), 1.12 (t, *J* = 3.2 Hz, 3H); **^{13}C NMR** (100 MHz, $CDCl_3$): δ 207.4, 142.0, 133.9, 132.6, 128.0, 127.3, 118.3, 112.0, 104.7, 79.5, 25.1, 12.5; **HRMS** (ESI): $[M+H]^+$ Calcd for $C_{12}H_{12}N$: 170.0964; found: 170.0960.

Experimental procedures and characterization data for products

Preparation of $ZnCl_2$ Solution (1.0 M in THF)

Zinc chloride (1.0 M in THF): Finely powdered anhydrous $ZnCl_2$ was weighed into a flame-dried Schlenk tube in the glove box in an inert atmosphere. The tube was heated with a heat gun for 2 min under vacuum and then back filled with nitrogen (This process was repeated three times.). After cooling, the flask was backfilled with N_2, and THF (anhydrous, 1.0 M) was added. The suspension was vigorously stirred for 30 min before it was used.

General procedure A for preparation of ^{n}BuZnCl from transmetallation of ^{n}BuLi to $ZnCl_2$

To a solution of $ZnCl_2$ (1.0 M) in THF was added ^{n}BuLi (2.4 M, 1.0 equiv) dropwise at 0 ℃. The mixture was vigorously stirred at the same temperature for 30 min.

General procedure B for the preparation of organozinc reagents via direct insertion of zinc

Anhydrous LiCl (2.0 equiv) was placed in an N_2-flushed sealed tube and dried for 10 min under the vacuum with heat gun. Zinc powder (2.0 equiv) was added under N_2 and the heterogeneous mixture of Zn and LiCl was dried again for 10 min under the vacuum with heat gun. The sealed tube was backfilled with N_2 (This process was repeated for three times). THF (1.0 M) was added and the Zn was activated with $BrCH_2CH_2Br$ (5 mol%) at 85 ℃ for 30 min. After cooling to rt, Me_3SiCl (1 mol%) and I_2 (0.5 mol%) was added under N_2, then the mixture was refluxed for another 20 min. Alkyl bromide (1.0 equiv)and the reaction mixture was stirred at was then added neat at room temperature and the reaction mixture was stirred at a 65 °C oil bath for 12-48 h.

All the above organozinc reagents are titrated according to the literature.[2]

Procedure for entry 1 of table 1

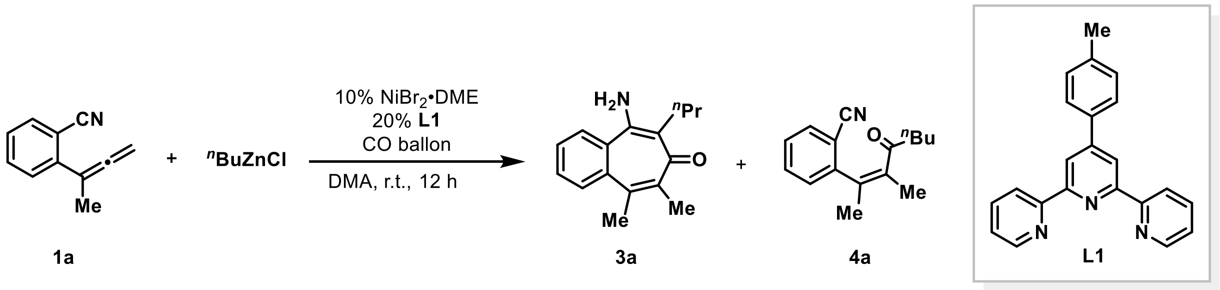

A 10 mL oven-dried tube charged with $NiBr_2 \cdot DME$ (10 mol%, 0.01 mmol, 3.1 mg) and **L1** (20 mol%, 0.02 mmol, 6.4 mg) was evacuated and backfilled with N_2 three times. The reaction mixture was evacuated again and backfilled with CO (1 atm, balloon), followed by addition of DMA (0.1 M), **1a** (1.0 equiv, 0.1 mmol, 15.5 mg) and alkylzinc reagent (1.5 equiv) at r.t. The tube was screw-capped and the reaction mixture was allowed to stir at r.t. for 12 h. The mixture was quenched with saturated aqueous NH_4Cl and extracted with EtOAc. The separated organic layer was washed with brine, dried over anhydrous Na_2SO_4, and concentrated under reduced pressure to yield the **3a** (33%) and **4a** (28%, E/Z = 1/16, **^{1}H NMR** (400 MHz, $CDCl_3$): δ 7.65 (dd, J = 7.6, 1.2 Hz, 1H), 7.51 (td, J = 7.6, 1.6 Hz, 1H), 7.35 (td, J = 8.0, 1.2 Hz, 1H), 7.17 (d, J = 7.2 Hz, 1H), 2.17 (t, J = 7.2 Hz, 2H), 2.12 (q, J = 1.2 Hz, 3H), 2.03

(q, J = 1.2 Hz, 3H), 1.33 (quint, J = 7.6 Hz, 2H), 1.08 (sext, J = 7.6 Hz, 2H), 0.76 (t, J = 7.2 Hz, 3H); ^{13}**C NMR** (100 MHz, $CDCl_3$) δ 207.4, 148.2, 138.3, 135.8, 133.1, 132.9, 129.5, 127.7, 118.1, 111.0, 41.7, 26.0, 22.2, 21.2, 16.3, 13.9), which was determined by ^{1}H NMR using CH_2Br_2 as the internal standard.

General procedure for the acylzincation of allenes

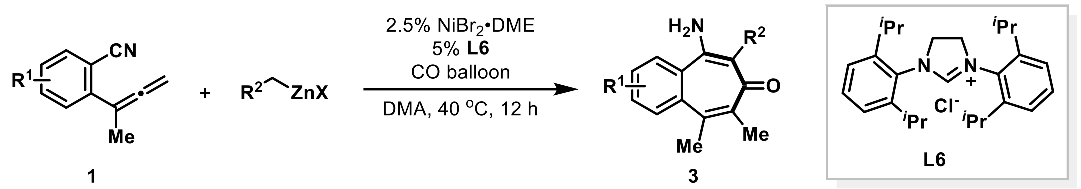

A 10 mL oven-dried tube charged with $NiBr_2$ ·DME (2.5 mol%) and **L6** (5 mol%) was evacuated and backfilled with N_2 three times. The reaction mixture was evacuated again and backfilled with CO (1 atm, balloon), followed by addition of DMA (0.1 M), allene (1.0 equiv) and alkylzinc reagent (1.5 equiv) at r.t. The tube was screw-capped and the reaction mixture was allowed to stir at 40 °C oil bath for 12 h. The mixture was quenched with saturated aqueous NH_4Cl and extracted with EtOAc. The separated organic layer was washed with brine, dried over anhydrous Na_2SO_4, and concentrated under reduced pressure to yield the crude product, which was purified by silica gel flash column chromatography.

Characterization Data for products

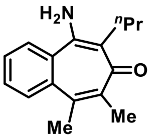

5-amino-8,9-dimethyl-6-propyl-7*H*-benzo[7]annulen-7-one (3a)

General procedure was followed with **1a** on 0.2 mmol scale with butylzinc chloride (0.40 M, prepared according to the general procedure A). The reaction mixture was purified by flash column chromatography (PE/EtOAc = 3/1) to afford **3a** as a colorless oil (34.2 mg, 71%). R_f = 0.42 (PE/EtOAc = 3/1); ^{1}**H NMR** (400 MHz, $CDCl_3$): δ 7.77 (dd, J = 8.0, 1.6 Hz, 1H), 7.61 (dd, J = 8.0, 1.6 Hz, 1H), 7.37 (td, J = 7.6, 1.6 Hz, 1H), 7.32 (td, J = 7.6, 1.6 Hz, 1H), 4.24 (bs, 2H), 2.52 (t, J = 7.6 Hz, 2H), 2.31 (s, 3H), 2.16 (s, 3H), 1.56 (sext, J = 7.6 Hz, 2H), 0.97 (t, J = 7.6 Hz, 3H); ^{13}**C NMR** (100 MHz, $CDCl_3$): δ 196.1, 147.3, 141.9, 138.1, 133.3, 133.2, 127.9, 127.7, 127.1, 125.8, 123.3, 32.2, 21.3, 21.1, 18.6, 14.4; **HRMS** (ESI): $[M+H]^+$ Calcd for $C_{16}H_{20}NO$: 242.1539; found: 242.1524.

5-amino-8,9-dimethyl-6-phenethyl-7*H*-benzo[7]annulen-7-one (3b)

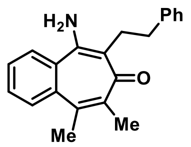

General procedure was followed with **1a** on 0.2 mmol scale with (3-phenylpropyl)zinc(II) bromide (0.65 M, prepared according to the general procedure B). The reaction mixture was purified by flash column chromatography (PE/EtOAc = 5/1-3/1) to afford **3b** as a colorless oil (40.8 mg, 67%). R_f = 0.49 (PE/EtOAc = 3/1); ^{1}**H NMR** (400 MHz, $CDCl_3$): δ 7.69 (dd, J = 8.0, 1.6 Hz, 1H), 7.63 (dd, J = 8.0, 1.2 Hz, 1H), 7.38 (td, J = 7.2, 1.6 Hz, 1H), 7.31 (td, J = 8.0, 1.2 Hz, 1H), 7.28–7.24 (m, 3H), 7.24–7.18 (m, 2H), 4.03 (bs, 2H), 2.89–2.80 (m, 4H), 2.33 (q, J = 0.8 Hz, 3H), 2.11 (q, J = 0.8 Hz, 3H); ^{13}**C NMR** (100 MHz, $CDCl_3$): δ 195.6, 148.2, 142.2, 142.0, 138.1, 133.4, 133.3, 128.7, 128.6, 127.9, 127.8, 127.2, 126.1, 125.7, 122.3, 34.5, 32.6, 21.2, 18.4.; **HRMS** (ESI): $[M+H]^+$ Calcd for $C_{21}H_{22}NO$: 304.1696; found: 304.1689.

5-amino-8,9-dimethyl-6-nonyl-7*H*-benzo[7]annulen-7-one (3c)

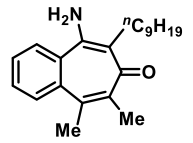

General procedure was followed with **1a** on 0.1 mmol scale with decylzinc(II) bromide (0.51 M, prepared according to the general procedure B). The reaction mixture was purified by flash column chromatography (PE/EtOAc = 10/1-5/1) to afford **3c** as a colorless oil (17.5 mg, 55%). R_f = 0.52 (PE/EtOAc = 5/1); **^{1}H NMR** (400 MHz, $CDCl_3$): δ 7.78 (dd, *J* = 8.0, 1.2 Hz, 1H), 7.62 (dd, *J* = 8.0, 1.2 Hz, 1H), 7.38 (td, *J* = 7.2, 1.2 Hz, 1H), 7.33 (td, *J* = 7.6, 1.2 Hz, 1H), 4.19 (bs, 2H), 2.53 (t, *J* = 7.6 Hz, 2H), 2.32 (s, 3H), 2.17 (s, 3H), 1.53 (quint, *J* = 7.6 Hz, 2H), 1.33–1.27 (m, 12H), 0.88 (t, *J* = 6.8 Hz, 3H); **^{13}C NMR** (100 MHz, $CDCl_3$): δ 196.1, 147.0, 142.0, 138.2, 133.3, 133.2, 127.9, 127.7, 127.1, 125.8, 123.7, 32.0, 30.3, 30.1, 29.8, 29.8, 29.5, 28.2, 22.8, 21.1, 18.7, 14.3; **HRMS** (ESI): $[M+H]^+$ Calcd for $C_{22}H_{32}NO$: 326.2478; found: 326.2461.

5-amino-8,9-dimethyl-6-phenethyl-7*H*-benzo[7]annulen-7-one (3d)

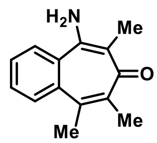

General procedure was followed with **1a** on 0.1 mmol scale with ethylzinc(II) bromide (0.50 M, prepared from transmetallation of EtMgBr with $ZnCl_2$). The reaction mixture was purified by flash column chromatography (PE/EtOAc = 5/1-3/1) to afford **3d** as a colorless oil (7.6 mg, 35%). R_f = 0.20 (PE/EtOAc = 5/1); **^{1}H NMR** (400 MHz, $CDCl_3$): δ 7.78 (dd, *J* = 8.0, 1.2 Hz, 1H), 7.66 (dd, *J* = 7.6, 1.2 Hz, 1H), 7.40 (td, *J* = 7.2, 1.2 Hz, 1H), 7.35 (td, *J* = 8.0, 1.2 Hz, 1H), 4.24 (bs, 2H), 2.34 (q, *J* = 0.8 Hz, 3H), 2.19 (q, *J* = 0.8 Hz, 3H), 2.09 (s, 3H); **^{13}C NMR** (100 MHz, $CDCl_3$): δ 195.3, 147.7, 141.9, 138.2, 133.9, 132.7, 128.1, 127.9, 127.3, 125.8, 118.1, 21.5, 18.8, 15.4.; **HRMS** (ESI): $[M+H]^+$ Calcd for $C_{14}H_{16}NO$: 214.1226; found: 214.1221.

5-amino-6-isopropyl-8,9-dimethyl-7*H*-benzo[7]annulen-7-one (3e)

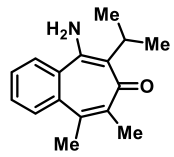

General procedure was followed with **1a** on 0.1 mmol scale with isopropylzinc(II) bromide (0.53 M, prepared according to the general procedure B). The reaction mixture was purified by flash column chromatography (PE/EtOAc = 10/1-5/1) to afford **3e** as a colorless oil (12.1 mg, 50%). R_f = 0.65 (PE/EtOAc = 5/1); **^{1}H NMR** (400 MHz, $CDCl_3$): δ 7.83 (dd, *J* = 7.6, 1.2 Hz, 1H), 7.57 (dd, *J* = 7.6, 1.6 Hz, 1H), 7.34 (td, *J* = 7.2, 1.2 Hz, 1H), 7.29 (td, *J* = 7.2, 1.2 Hz, 1H), 4.08 (bs, 2H), 2.79 (quint, *J* = 6.8 Hz, 1H), 2.25 (s, 3H), 2.11 (s, 3H), 1.28 (d, *J* = 6.8 Hz, 6H); **^{13}C NMR** (100 MHz, $CDCl_3$): δ 198.9, 143.2, 142.2, 138.5, 133.7, 131.2, 127.5, 127.5, 127.2, 126.7, 126.7, 29.4, 20.9, 20.0, 17.3; **HRMS** (ESI): $[M+H]^+$ Calcd for $C_{16}H_{20}NO$: 242.1539; found: 242.1526.

5-amino-8,9-dimethyl-6-(pent-4-en-1-yl)-7*H*-benzo[7]annulen-7-one (3f)

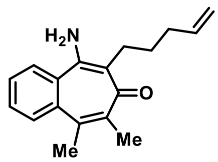

General procedure was followed with **1a** on 0.1 mmol scale with hex-5-en-1-ylzinc(II) bromide (0.58 M, prepared according to the general procedure B). The reaction mixture was purified by flash column chromatography (PE/EtOAc = 20/1-10/1) to afford **3f** as a colorless oil (16.3 mg, 61%). R_f = 0.33 (PE/EtOAc = 10/1); **^{1}H NMR** (400 MHz, $CDCl_3$): δ 7.77 (dd, *J* = 8.0, 1.2 Hz, 1H), 7.61 (dd, *J* = 8.0, 1.2 Hz, 1H), 7.37 (td, *J* = 7.2, 1.2 Hz, 1H), 7.30 (td, *J* = 8.0, 1.2 Hz, 1H), 5.86 (ddt, *J* = 16.8, 10.0, 6.4 Hz, 1H), 5.05 (dd, *J* = 17.2, 2.0 Hz, 1H), 5.00 (dd, *J* = 10.4, 1.2 Hz, 1H), 4.30 (bs, 2H), 2.52 (t, *J* = 7.6 Hz, 2H), 2.31 (s, 3H), 2.13 (s, 3H), 2.12 (dt, *J* = 7.2， 7.2 Hz, 2H)，1.62 (quint, *J* = 7.6 Hz, 2H); **^{13}C NMR** (100 MHz, $CDCl_3$): δ 195.8,

147.5, 141.9, 138.8, 138.1, 133.3, 133.2, 127.9, 127.7, 127.1, 125.8, 122.9, 115.0, 33.8, 29.5, 27.1, 21.2, 18.6; **HRMS** (ESI): $[M+H]^+$ Calcd for $C_{18}H_{22}NO$: 268.1696; found: 268.1682.

(*S*)-5-amino-6-(2,6-dimethylhept-5-en-1-yl)-8,9-dimethyl-7*H*-benzo[7]annulen-7-one (3g)

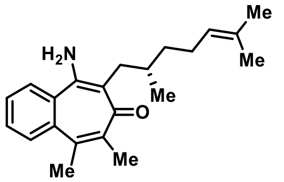

General procedure was followed with **1a** on 0.1 mmol scale with (*S*)-(3,7-dimethyloct-6-en-1-yl)zinc(II) bromide (0.30 M, prepared according to the general procedure B). The reaction mixture was purified by flash column chromatography (PE/EtOAc = 5/1) to afford **3g** as a colorless oil (20.9 mg, 65%). R_f = 0.49 (PE/EtOAc = 5/1); **^{1}H NMR** (400 MHz, $CDCl_3$): δ 7.76 (dd, *J* = 7.6, 1.2 Hz, 1H), 7.61 (dd, *J* = 7.6, 1.2 Hz, 1H), 7.37 (td, *J* = 7.2, 0.8 Hz, 1H), 7.32 (td, *J* = 7.6, 1.2 Hz, 1H), 5.09 (t, *J* = 6.8 Hz, 1H), 4.20 (bs, 2H), 2.50 (d, *J* = 7.6 Hz, 2H), 2.32 (s, 3H), 2.16 (s, 3H), 2.10 (quint, *J* = 7.2 Hz, 1H), 1.99 (quint, *J* = 7.2 Hz, 1H), 1.74 (sext, *J* = 7.2 Hz, 1H), 1.68 (s, 3H), 1.61 (s, 3H), 1.47–1.38 (m, 1H), 1.29–1.27 (m, 1H), 0.92 (d, *J* = 6.8 Hz, 3H); **^{13}C NMR** (100 MHz, $CDCl_3$) δ 196.7, 147.4, 142.0, 138.2, 133.4, 133.1, 131.6, 127.8, 127.7, 127.1, 125.7, 124.9, 122.6, 37.5, 37.2, 31.9, 25.9, 25.8, 21.0, 20.0, 19.0, 17.9; **HRMS** (ESI): $[M+H]^+$ Calcd for $C_{22}H_{30}NO$: 324.2322; found: 324.2314.

5-amino-6-(5-chloropentyl)-8,9-dimethyl-7*H*-benzo[7]annulen-7-one (3h)

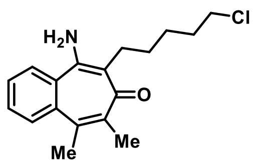

General procedure was followed with **1a** on 0.2 mmol scale with (6-chlorohexyl)zinc(II) bromide (0.81 M, prepared according to the general procedure B). The reaction mixture was purified by flash column chromatography (PE/EtOAc = 5/1-3/1) to afford **3h** as a colorless oil (30.5 mg, 50%). R_f = 0.39 (PE/EtOAc = 3/1); **^{1}H NMR** (400 MHz, $CDCl_3$): δ 7.78 (d, *J* = 7,6 Hz, 1H), 7.63 (d, *J* = 8.0 Hz, 1H), 7.39 (t, *J* = 7.6 Hz, 1H), 7.34 (t, *J* = 8.0 Hz, 1H), 4.25 (bs, 2H), 3.54 (t, *J* = 6.8 Hz, 2H), 2.54 (t, *J* = 7.2 Hz, 2H), 2.32 (s, 3H), 2.16 (s, 3H), 1.82 (quint, *J* = 7.2 Hz, 2H), 1.61–1.49 (m, 4H); **^{13}C NMR** (100 MHz, $CDCl_3$): δ 195.8, 147.3, 142.0, 138.2, 133.5, 133.2, 128.0, 127.8, 127.2, 125.8, 123.0, 45.3, 32.6, 30.1, 27.3, 27.2, 21.2, 18.7; **HRMS** (ESI): $[M+H]^+$ Calcd for $C_{18}H_{23}ClNO$: 304.1463; found: 304.1453.

5-amino-6-(4-fluorobutyl)-8,9-dimethyl-7*H*-benzo[7]annulen-7-one (3i)

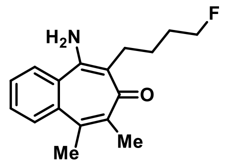

General procedure was followed with **1a** on 0.2 mmol scale with (5-fluoropentyl)zinc(II) bromide (0.67 M, prepared according to the general procedure B). The reaction mixture was purified by flash column chromatography (PE/EtOAc = 3/1) to afford **3i** as a colorless oil (29.4 mg, 54%). R_f = 0.41 (PE/EtOAc = 3/1); **^{1}H NMR** (400 MHz, $CDCl_3$): δ 7.79 (dd, *J* = 8.0, 1.2 Hz, 1H), 7.63 (dd, *J* = 8.0, 1.2 Hz, 1H), 7.40 (td, *J* = 7.6, 1.2 Hz, 1H), 7.35 (td, *J* = 7.6, 1.2 Hz, 1H), 4.57 (t, *J* = 5.6 Hz, 2H), 4.45 (t, *J* = 5.6 Hz, 2H), 4.27 (bs, 2H), 2.58 (t, *J* = 7.6 Hz, 2H), 2.33 (s, 3H), 2.16 (s, 3H), 1.85–1.77 (m, 2H), 1.75–1.66 (m, 2H); **^{13}C NMR** (100 MHz, $CDCl_3$): δ 195.7, 147.7, 142.0, 138.2, 133.6, 133.1, 128.0, 127.9, 127.2, 125.9, 122.6, 84.3 (d, $J_{C\text{-}F}$ = 162.9 Hz), 30.3 (d, $J_{C\text{-}F}$ = 19.2 Hz), 29.6, 23.6 (d, $J_{C\text{-}F}$ = 4.8 Hz), 21.3, 18.6; **^{19}F NMR** (376 MHz, $CDCl_3$): -218.3; **HRMS** (ESI): $[M+H]^+$ Calcd for $C_{17}H_{21}FNO$: 274.1602; found: 274.1597.

5-(5-amino-8,9-dimethyl-7-oxo-7*H*-benzo[7]annulen-6-yl)pentyl pivalate (3j)

General procedure was followed with **1a** on 0.1 mmol scale with (6-(pivaloyloxy)hexyl)zinc(II) bromide (0.435 M, prepared according to the general procedure B). The reaction mixture was purified by flash

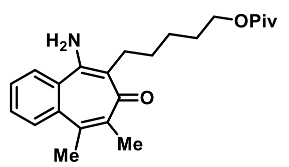

column chromatography (PE/EtOAc = 5/1) to afford **3j** as a colorless oil (19.9 mg, 54%). R_f = 0.41 (PE/EtOAc = 5/1); **^{1}H NMR** (400 MHz, $CDCl_3$): δ 7.77 (d, *J* = 7.6 Hz, 1H), 7.62 (d, *J* = 8.0 Hz, 1H), 7.37 (td, *J* = 7.2, 1.2 Hz, 1H), 7.32 (td, *J* = 7.6, 1.6 Hz, 1H), 4.27 (bs, 2H), 4.05 (t, *J* = 6.8 Hz, 2H), 2.53 (t, *J* = 7.6 Hz, 2H), 2.31 (s, 3H), 2.15 (s, 3H), 1.66 (sext, *J* = 7.2 Hz, 2H), 1.57 (quint, *J* = 7.6 Hz, 2H), 1.42 (quint, *J* = 7.2 Hz, 2H), 1.18 (s, 9H); **^{13}C NMR** (100 MHz, $CDCl_3$): δ 195.8, 178.9, 147.5, 141.9, 138.1, 133.4, 133.2, 127.9, 127.8, 127.2, 125.8, 123.0, 64.4, 38.9, 30.1, 28.6, 27.5, 27.3, 26.1, 21.2, 18.6; **HRMS** (ESI): $[M+H]^+$ Calcd for $C_{23}H_{32}NO_3$: 370.2377; found: 370.2368.

ethyl 5-(5-amino-8,9-dimethyl-7-oxo-7*H*-benzo[7]annulen-6-yl)pentanoate (3k)

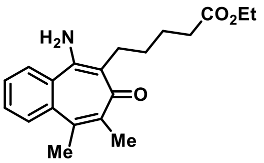

General procedure was followed with **1a** on 0.1 mmol scale with (6-ethoxy-6-oxohexyl)zinc(II) bromide (0.48 M, prepared according to the general procedure B). The reaction mixture was purified by flash column chromatography (PE/EtOAc = 3/1) to afford **3k** as a white solid (15.5 mg, 47%). R_f = 0.20 (PE/EtOAc = 5/1); **^{1}H NMR** (400 MHz, $CDCl_3$): δ 7.76 (d, *J* = 7.6 Hz, 1H), 7.61 (d, *J* = 7.6 Hz, 1H), 7.37 (t, *J* = 7.6 Hz, 1H), 7.31 (t, *J* = 7.2 Hz, 1H), 4.38 (bs, 2H), 4.11 (q, *J* = 7.2 Hz, 2H), 2.54 (t, *J* = 7.6 Hz, 2H), 2.35 (t, *J* = 7.2 Hz, 2H), 2.30 (s, 3H), 2.15 (s, 3H), 1.70 (quint, *J* = 7.2 Hz, 2H), 1.56 (quint, *J* = 7.6 Hz, 2H), 1.24 (t, *J* = 7.2 Hz, 3H); **^{13}C NMR** (100 MHz, $CDCl_3$): δ 209.9, 174.0, 147.6, 142.0, 138.2, 133.4, 133.3, 127.9, 127.8, 127.2, 125.8, 122.8, 60.4, 34.1, 29.7, 27.2, 25.1, 21.2, 18.6, 14.4; **HRMS** (ESI): $[M+H]^+$ Calcd for $C_{20}H_{26}NO_3$: 328.1907; found: 328.1903.

4-(5-amino-8,9-dimethyl-7-oxo-7*H*-benzo[7]annulen-6-yl)butanenitrile (3l)

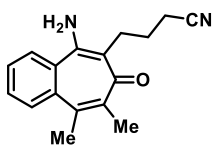

General procedure was followed with **1a** on 0.2 mmol scale with (4-cyanobutyl)zinc(II) bromide (0.64 M, prepared according to the general procedure B). The reaction mixture was purified by flash column chromatography (PE/EtOAc = 3/1-2/1) to afford **3l** as a white solid (17.2 mg, 32%). R_f = 0.53 (PE/EtOAc = 1/1); **^{1}H NMR** (400 MHz, $CDCl_3$): δ 7.79 (dd, *J* = 8.0, 1.2 Hz, 1H), 7.65 (dd, *J* = 8.0, 1.2 Hz, 1H), 7.42 (td, *J* = 7.6, 1.2 Hz, 1H), 7.36 (td, *J* = 8.0, 1.2 Hz, 1H), 4.40 (bs, 2H), 2.62 (t, *J* = 7.2 Hz, 2H), 2.42 (t, *J* = 7.2 Hz, 2H), 2.33 (s, 3H), 2.15 (s, 3H), 1.96 (quint, *J* = 7.2 Hz, 2H); **^{13}C NMR** (100 MHz, $CDCl_3$): δ 195.1, 148.7, 141.9, 138.2, 134.2, 132.8, 128.3, 128.1, 127.4, 126.0, 120.5, 120.2, 28.9, 23.8, 21.4, 18.6, 17.1; **HRMS** (ESI): $[M+H]^+$ Calcd for $C_{17}H_{19}N_2O$: 267.1492; found: 267.1484.

5-amino-2,8,9-trimethyl-6-propyl-7*H*-benzo[7]annulen-7-one (3m)

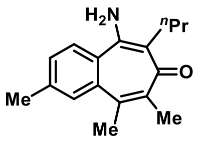

General procedure was followed with **1b** on 0.1 mmol scale with butylzinc chloride (0.41 M, prepared according to the general procedure A). The reaction mixture was purified by flash column chromatography (PE/EtOAc = 5/1) to afford **3m** as a colorless oil (16.1 mg, 63%). R_f = 0.41 (PE/EtOAc = 5/1); **^{1}H NMR** (400 MHz, $CDCl_3$): δ 7.66 (d, *J* = 8.4 Hz, 1H), 7.41 (s, 1H), 7.14 (dd, *J* = 8.4, 1.6 Hz, 1H), 4.20 (bs, 2H), 2.51 (t, *J* = 7.6 Hz, 2H), 2.40 (s, 3H), 2.31 (s, 3H), 2.16 (s, 3H), 1.56 (sext, *J* = 7.6 Hz, 2H), 0.97 (t, *J* = 7.2 Hz, 3H); **^{13}C NMR** (100 MHz, $CDCl_3$): δ 196.1, 147.4, 141.8, 138.2, 137.5, 133.2, 130.8, 128.2, 128.2, 125.8, 122.8, 32.2, 21.5, 21.4, 21.2, 18.7, 14.4; **HRMS** (ESI): $[M+H]^+$ Calcd for $C_{17}H_{22}NO$: 256.1696; found: 256.1686.

9-amino-2,5,6-trimethyl-8-propyl-7*H*-benzo[7]annulen-7-one (3n)

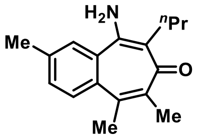

General procedure was followed with **1c** on 0.1 mmol scale with butylzinc chloride (0.38 M, prepared according to the general procedure A). The reaction mixture was purified by flash column chromatography (PE/EtOAc = 5/1) to afford **3n** as a colorless oil (15.8 mg, 62%). R_f = 0.35 (PE/EtOAc = 5/1); **^{1}H NMR** (400 MHz, $CDCl_3$): δ 7.59 (s, 1H), 7.52 (d, *J* = 8.4 Hz, 1H), 7.19 (d, *J* = 8.0 Hz, 1H), 4.21 (bs, 2H), 2.51 (t, *J* = 7.6 Hz, 2H), 2.39 (s, 3H), 2.30 (s, 3H), 2.15 (s, 3H), 1.56 (sext, *J* = 7.6 Hz, 2H), 0.98 (t, *J* = 7.2 Hz, 3H); **^{13}C NMR** (100 MHz, $CDCl_3$): δ 196.1, 147.2, 141.2, 136.9, 135.6, 133.4, 133.2, 128.8, 128.0, 126.0, 123.3, 32.3, 21.4, 21.3, 21.1, 18.6, 14.4; **HRMS** (ESI): $[M+H]^+$ Calcd for $C_{17}H_{22}NO$: 256.1696; found: 256.1688.

5-amino-2,3,8,9-tetramethyl-6-propyl-7H-benzo[7]annulen-7-one (3o)

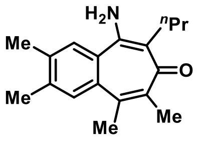

General procedure was followed with **1d** on 0.2 mmol scale with butylzinc chloride (0.40 M, prepared according to the general procedure A). The reaction mixture was purified by flash column chromatography (PE/EtOAc = 5/1) to afford **3o** as a colorless oil (32.9 mg, 61%). R_f = 0.34 (PE/EtOAc = 5/1); **^{1}H NMR** (400 MHz, $CDCl_3$): δ 7.54 (s, 1H), 7.37 (s, 1H), 4.21 (bs, 2H), 2.53 (t, *J* = 7.6 Hz, 2H), 2.31 (s, 3H), 2.30 (s, 6H), 2.15 (s, 3H), 1.56 (sext, *J* = 7.6 Hz, 2H), 0.97 (t, *J* = 7.2 Hz, 3H); **^{13}C NMR** (100 MHz, $CDCl_3$): δ 195.9, 147.3, 141.1, 136.5, 136.0, 135.9, 133.4, 131.0, 128.9, 126.5, 122.7, 32.2, 21.3, 21.3, 19.9, 19.8, 18.7, 14.5; **HRMS** (ESI): $[M+H]^+$ Calcd for $C_{18}H_{24}NO$: 270.1852; found: 270.1853.

5-amino-8,9-dimethyl-2-phenyl-6-propyl-7*H*-benzo[7]annulen-7-one (3p)

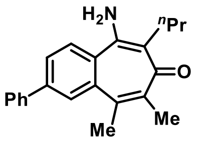

General procedure was followed with **1e** on 0.2 mmol scale with butylzinc chloride (0.42 M, prepared according to the general procedure A). The reaction mixture was purified by flash column chromatography (PE/EtOAc = 5/1) to afford **3p** as a colorless oil (36.1 mg, 57%). R_f = 0.34 (PE/EtOAc = 5/1); **^{1}H NMR** (400 MHz, $CDCl_3$): δ 7.84 (d, *J* = 8.0 Hz, 1H), 7.82 (d, *J* = 1.6 Hz, 1H), 7.62–7.60 (m, 2H), 7.54 (dd, *J* = 8.8, 2.0 Hz, 1H), 7.47 (t, *J* = 7.6 Hz, 2H), 7.39 (tt, *J* = 7.6, 1.2 Hz, 1H), 4.29 (bs, 2H), 2.55 (t, *J* = 7.6 Hz, 2H), 2.38 (s, 3H), 2.20 (s, 3H), 1.59 (sext, *J* = 7.6 Hz, 2H), 1.00 (t, *J* = 7.2 Hz, 3H); **^{13}C NMR** (100 MHz, $CDCl_3$): δ 196.1, 147.2, 142.2, 140.4, 140.4, 138.5, 133.2, 132.2, 129.0, 127.8, 127.3, 126.5, 126.5, 125.9, 123.4, 32.2, 21.3, 21.2, 18.8, 14.4; **HRMS** (ESI): $[M+H]^+$ Calcd for $C_{22}H_{24}NO$: 318.1852; found: 318.1843.

5-amino-2-methoxy-8,9-dimethyl-6-propyl-7*H*-benzo[7]annulen-7-one (3q)

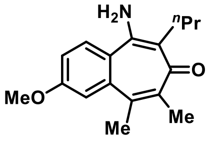

General procedure was followed with **1f** on 0.1 mmol scale with butylzinc chloride (0.40 M, prepared according to the general procedure A). The reaction mixture was purified by flash column chromatography (PE/EtOAc = 3/1) to afford **3q** as a colorless oil (18.2 mg, 67%). R_f = 0.42 (PE/EtOAc = 3/1); **^{1}H NMR** (400 MHz, $CDCl_3$): δ 7.72 (d, *J* = 8.8 Hz, 1H), 7.08 (d, *J* = 2.8 Hz, 1H), 6.89 (dd, *J* = 8.8, 2.8 Hz, 1H), 4.19 (bs, 2H), 3.85 (s, 3H), 2.50 (t, *J* = 7.6 Hz, 2H), 2.30 (q, *J* = 0.8 Hz, 3H), 2.16 (q, *J* = 0.8 Hz, 3H), 1.55 (sext, *J* = 7.6 Hz, 2H), 0.97 (t, *J* = 7.2 Hz, 3H); **^{13}C NMR** (100 MHz, $CDCl_3$): δ 195.9, 158.7, 147.4, 142.1, 139.8, 132.7, 127.7, 126.8, 122.1, 113.5, 112.3, 55.5, 32.1, 21.4, 21.3, 18.8, 14.4; **HRMS** (ESI): $[M+H]^+$ Calcd for $C_{17}H_{22}NO_2$: 272.1645; found: 272.1635.

5-amino-2,3-dimethoxy-8,9-dimethyl-6-propyl-7*H*-benzo[7]annulen-7-one (3r)

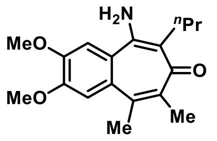

General procedure was followed with **1g** on 0.2 mmol scal with butylzinc chloride (0.40 M, prepared according to the general procedure A)e. The reaction mixture was purified by flash column chromatography (PE/EtOAc = 3/1-2/1) to afford **3r** as a colorless oil (25.2 mg, 42%). R_f = 0.21 (PE/EtOAc = 3/1); **^{1}H NMR** (400 MHz, $CDCl_3$): δ 7.26 (s, 1H), 7.04 (s, 1H), 4.13 (bs, 2H), 3.93 (s, 6H), 2.52 (t, *J* = 7.6 Hz, 2H), 2.31 (s, 3H), 2.16 (s, 3H), 1.56 (sext, *J* = 7.6 Hz, 2H), 0.97 (t, *J* = 7.2 Hz, 3H); **^{13}C NMR** (100 MHz, $CDCl_3$): δ 195.6, 148.4, 148.0, 146.8, 140.7, 133.0, 132.3, 127.0, 123.0, 110.1, 108.2, 56.1, 56.0, 32.2, 21.5, 21.4, 18.7, 14.5; **HRMS** (ESI): $[M+H]^+$ Calcd for $C_{18}H_{24}NO_3$: 302.1751; found: 302.1753.

5-amino-8,9-dimethyl-6-propyl-7*H*-cyclohepta[4,5]benzo[1,2-d][1,3]dioxol-7-one (3s)

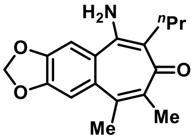

General procedure was followed with **1h** on 0.2 mmol scale with butylzinc chloride (0.40 M, prepared according to the general procedure A). The reaction mixture was purified by flash column chromatography (PE/EtOAc = 5/1-3/1) to afford **3s** as a colorless oil (36.0 mg, 63%). R_f = 0.32 (PE/EtOAc = 5/1); **^{1}H NMR** (400 MHz, $CDCl_3$): δ 7.25 (s, 1H), 7.03 (s, 1H), 6.00 (s, 2H), 4.10 (bs, 2H), 2.50 (t, *J* = 7.6 Hz, 2H), 2.25 (s, 3H), 2.14 (s, 3H), 1.54 (sext, *J* = 7.6 Hz, 2H), 0.96 (t, *J* = 7.2 Hz, 3H); **^{13}C NMR** (100 MHz, $CDCl_3$): δ 195.7, 147.4, 147.1, 146.9, 140.6, 133.8, 133.0, 128.2, 122.7, 107.2, 105.3, 101.7, 32.2, 21.6, 21.3, 18.7, 14.4; **HRMS** (ESI): $[M+H]^+$ Calcd for $C_{17}H_{20}NO_3$: 286.1438; found: 286.1429.

5-amino-2-chloro-8,9-dimethyl-6-propyl-7*H*-benzo[7]annulen-7-one (3t)

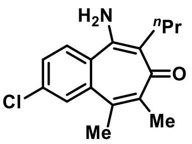

General procedure was followed with **1i** on 0.1 mmol scale with butylzinc chloride (0.40 M, prepared according to the general procedure A). The reaction mixture was purified by flash column chromatography (PE/EtOAc = 5/1-3/1) to afford **3t** as a colorless oil (16.6 mg, 30%). R_f = 0.48 (PE/EtOAc = 3/1); **^{1}H NMR** (400 MHz, $CDCl_3$): δ 7.72 (d, *J* = 8.4 Hz, 1H), 7.57 (d, *J* = 2.4 Hz, 1H), 7.27 (dd, *J* = 8.4, 2.4 Hz, 1H), 4.16 (bs, 2H), 2.50 (t, *J* = 7.6 Hz, 2H), 2.29 (s, 3H), 2.15 (s, 3H), 1.55 (sext, *J* = 7.6 Hz, 2H), 0.97 (t, *J* = 7.2 Hz, 3H); **^{13}C NMR** (100 MHz, $CDCl_3$): δ 196.0, 146.3, 143.2, 139.7, 133.6, 131.9, 131.8, 127.7, 127.6, 127.2, 123.7, 32.2, 21.3, 21.0, 18.8, 14.4; **HRMS** (ESI): $[M+H]^+$ Calcd for $C_{16}H_{19}ClNO$: 276.1150; found: 276.1138.

9-amino-2-fluoro-5,6-dimethyl-8-propyl-7*H*-benzo[7]annulen-7-one (3u)

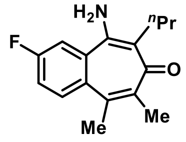

General procedure was followed with **1j** on 0.1 mmol scale with butylzinc chloride (0.41 M, prepared according to the general procedure A). The reaction mixture was purified by flash column chromatography (PE/EtOAc = 5/1) to afford **3u** as a colorless oil (13.7 mg, 53%). R_f = 0.50 (PE/EtOAc = 5/1); **^{1}H NMR** (400 MHz, $CDCl_3$): δ 7.60 (dd, *J* = 8.8, 5.6 Hz, 1H), 7.48 (dd, *J* = 10.8, 2.8 Hz, 1H), 7.08 (ddd, *J* = 10.4, 7.6, 2.8 Hz, 1H), 4.14 (bs, 2H), 2.51 (t, *J* = 7.6 Hz, 2H), 2.29 (s, 3H), 2.14 (s, 3H), 1.55 (sext, *J* = 7.6 Hz, 2H), 0.97 (t, *J* = 7.6 Hz, 3H); **^{13}C NMR** (100 MHz, $CDCl_3$): δ 196.2, 161.1 (d, J_{C-F} = 245.1 Hz), 145.9, 141.5, 135.2 (d, J_{C-F} = 7.4 Hz), 134.6 (d, J_{C-F} = 3.4 Hz), 132.7, 130.4 (d, J_{C-F} = 8.0 Hz), 124.0, 115.2 (d, J_{C-F} = 21.1 Hz), 112.2 (d, J_{C-F} = 23.1 Hz), 32.3, 21.2, 21.2, 18.6, 14.4; **^{19}F NMR** (376 MHz, $CDCl_3$): -111.1; **HRMS** (ESI): $[M+H]^+$ Calcd for $C_{16}H_{19}FNO$: 260.1445; found: 260.1433.

9-amino-1-fluoro-5,6-dimethyl-8-propyl-7*H*-benzo[7]annulen-7-one (3v)

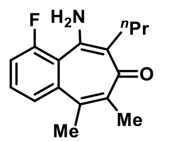

General procedure was followed with **1k** on 0.2 mmol scale with butylzinc chloride (0.40 M, prepared according to the general procedure A). The reaction mixture was purified by flash column chromatography (PE/EtOAc = 5/1) to afford **3v** as a colorless oil (16.6 mg, 32%). R_f = 0.45 (PE/EtOAc = 5/1); **^{1}H NMR** (400 MHz, $CDCl_3$): δ 7.36 (d, *J* = 8.0 Hz, 1H), 7.28 (td, *J* = 8.0, 5.6 Hz, 1H), 7.02 (dd, *J* = 12.4, 8.0 Hz, 1H), 4.53 (bs, 2H), 2.54 (t, *J* = 7.6 Hz, 2H), 2.28 (s, 3H), 2.11 (s, 3H), 1.55 (sext, *J* = 7.6 Hz, 2H), 0.97 (t, *J* = 7.2 Hz, 3H); **^{13}C NMR** (100 MHz, $CDCl_3$): δ 197.3, 159.0 (d, J_{C-F} = 245.7 Hz), 142.9 (d, J_{C-F} = 22.7 Hz), 140.4 (d, J_{C-F} = 2.4 Hz), 132.6 (d, J_{C-F} = 2.7 Hz), 128.1 (d, J_{C-F} = 10.6 Hz), 124.7, 123.4, 123.4, 121.1 (d, J_{C-F} = 10.9 Hz), 114.0 (d, J_{C-F} = 25.1 Hz), 32.9, 21.7, 21.1, 18.5, 14.3; **^{19}F NMR** (376 MHz, $CDCl_3$): -114.2; **HRMS** (ESI): $[M+H]^+$ Calcd for $C_{16}H_{19}FNO$: 260.1445; found: 260.1447.

5-amino-9-ethyl-8-methyl-6-propyl-7*H*-benzo[7]annulen-7-one (3w)

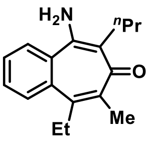

General procedure was followed with **1l** on 0.2 mmol scale with butylzinc chloride (0.40 M, prepared according to the general procedure A). The reaction mixture was purified by flash column chromatography (PE/EtOAc = 5/1) to afford **3w** as a colorless oil (30.7 mg, 60%). R_f = 0.33 (PE/EtOAc = 5/1); **^{1}H NMR** (400 MHz, $CDCl_3$): δ 7.78 (dd, *J* = 7.6, 1.2 Hz, 1H), 7.70 (d, *J* = 7.6 Hz, 1H), 7.37 (td, *J* = 7.6, 1.2 Hz, 1H), 7.32 (td, *J* = 7.2, 1.2 Hz, 1H), 4.22 (bs, 2H), 2.75 (q, *J* = 7.2 Hz, 2H), 2.50 (t, *J* = 7.6 Hz, 2H), 2.19 (s, 3H), 1.56 (sext, *J* = 7.6 Hz, 2H), 1.18 (t, *J* = 7.6 Hz, 3H), 0.98 (t, *J* = 7.6 Hz, 3H); **^{13}C NMR** (100 MHz, $CDCl_3$): δ 197.4, 147.5, 141.0, 137.8, 137.0, 134.1, 127.7, 127.5, 127.1, 126.0, 123.3, 32.2, 27.1, 21.3, 18.3, 14.4, 13.7; **HRMS** (ESI): $[M+H]^+$ Calcd for $C_{17}H_{22}NO$: 256.1696; found: 256.1688.

5-amino-9-butyl-8-methyl-6-propyl-7*H*-benzo[7]annulen-7-one (3x)

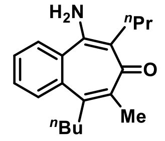

General procedure was followed with **1m** on 0.2 mmol scale with butylzinc chloride (0.40 M, prepared according to the general procedure A). The reaction mixture was purified by flash column chromatography (PE/EtOAc = 5/1-1/1) to afford **3x** as a colorless oil (28.8 mg, 51%). R_f = 0.51 (PE/EtOAc = 3/1); **^{1}H NMR** (400 MHz, $CDCl_3$): δ 7.76 (dd, *J* = 7.6, 1.6 Hz, 1H), 7.67 (dd, *J* = 8.0, 1.2 Hz, 1H), 7.36 (td, *J* = 7.6, 1.6 Hz, 1H), 7.31 (td, *J* = 7.6, 1.6 Hz, 1H), 4.22 (bs, 2H), 2.72 (t, *J* = 8.0 Hz, 2H), 2.50 (t, *J* = 8.0 Hz, 2H), 2.17 (s, 3H), 1.56 (sext, *J* = 7.6 Hz, 2H), 1.51–1.45 (m, 2H), 1.33 (sext, *J* = 7.2 Hz, 2H), 0.97 (t, *J* = 7.6 Hz, 3H), 0.90 (t, *J* = 7.2 Hz, 3H); **^{13}C NMR** (100 MHz, $CDCl_3$): δ 197.6, 147.4, 141.2, 137.1, 136.8, 134.1, 127.6, 127.6, 127.0, 125.9, 123.3, 33.6, 32.2, 31.2, 23.1, 21.3, 18.5, 14.3, 14.0; HRMS (ESI): $[M+H]^+$ Calcd for $C_{19}H_{26}NO$: 284.2009; found: 284.2011.

Synthetic application

(i) 1.0-mmol scale reaction

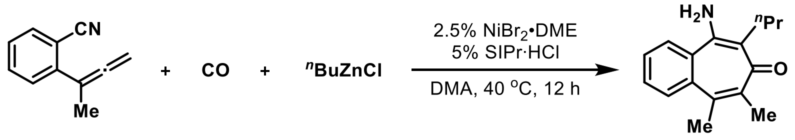

A 500 mL sealed tube charged with NiBr$_2$·DME (2.5 mol%, 7.7 mg) and SIPr·HCl (5 mol%, 21.5 mg) was evacuated and backfilled with N_2 three times. The reaction mixture was evacuated again and backfilled with CO (1 atm, balloon), followed by addition of DMA (10 mL), allene (1.0 equiv, 1.0 mmol, 155 mg) and ^{n}BuZnCl (1.5 equiv, 1.5 mmol, 3.6 mL, 0.42 M in THF) at r.t. The tube was screw-capped and reaction mixture was allowed to warm to 40 °C oil bath for 12 h. The mixture was quenched with saturated aqueous NH_4Cl and extracted with EtOAc. The separated organic layer was washed with brine, dried over anhydrous Na_2SO_4, and concentrated under reduced pressure to yield the crude product, which was purified by silica gel flash column chromatography (183.2 mg, 76%).

(ii) Derivatization

a) Procedure for the synthesis of 8,9-dimethyl-6-propyl-5*H*-benzo[7]annulene-5,7(6*H*)-dione

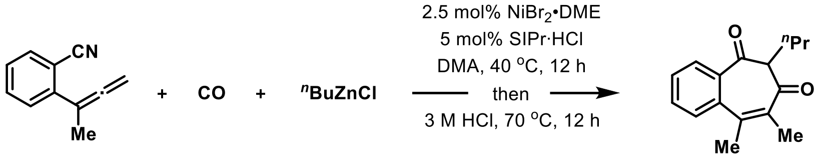

1a **5**

A 20 mL oven-dried tube charged with NiBr$_2$·DME (2.5 mol%, 0.8 mg) and SIPr·HCl (5 mol%, 2.2 mg) was evacuated and backfilled with N_2 three times. The reaction mixture was evacuated again and backfilled with CO (1 atm, balloon), followed by addition of DMA (0.1 M, 1 mL), **1a** (0.1 mmol, 15.5 mg) and ^{n}BuZnCl (1.5 equiv, 0.15 mmol, 0.36 mL, 0.42 M in THF) at r.t. The tube was screw-capped and the reaction mixture was allowed to stir at 40 °C oil bath for 12 h. The mixture was quenched with 3 M HCl (3 mL) and stirred for 12 h at 70 °C. After cooling to r.t., The mixture was neutralized with saturated $NaHCO_3$ and extracted with EtOAc. The separated organic layer was washed with brine, dried over anhydrous Na_2SO_4, and concentrated under reduced pressure to yield the crude product **5**, which was purified by silica gel flash column chromatography (11.6 mg, 48%).

^{1}H NMR (400 MHz, $CDCl_3$): δ 7.47 (ddd, *J* = 8.4, 6.8, 2.0 Hz, 1H), 7.35–7.28 (m, 3H), 3.70 (t, *J* = 7.2 Hz, 1H), 2.17 (s, 3H), 2.00 (s, 3H), 1.95–1.78 (m, 2H), 1.36–1.30 (m, 2H), 0.92 (t, *J* = 7.2 Hz, 3H);

^{13}C NMR (100 MHz, $CDCl_3$): δ 203.4, 202.9, 138.1, 137.9, 137.3, 135.9, 131.6, 128.0, 127.8, 126.9, 71.6, 29.8, 20.7, 20.4, 17.2, 14.0;

HRMS (ESI): $[M+H]^+$ Calcd for $C_{16}H_{19}O_2$: 243.1380; found: 243.1383.

b) Procedure for the synthesis of 8,9-dimethyl-6-propyl-5H-benzo[7]annulen-5-one

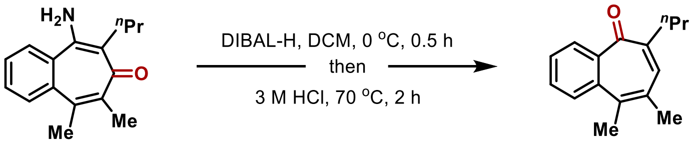

3a **6**

To a solution of **3a** (1.0 equiv, 0.05 mmol, 12.1 mg) in DCM (1 mL) was added DIBAL-H (3.0 equiv, 1.0 M in hexane, 0.15 mL) dropwise at 0 °C. After stirring for 0.5 h at same temperature, the reaction was quenched with 3 M HCl (2 mL) at 0 °C, and then the resulted mixture was stirred at 70 ℃ for 2 h. After cooling to r.t., The mixture was neutralized with saturated $NaHCO_3$ and extracted with EtOAc. The separated organic layer was washed with brine, dried over anhydrous Na_2SO_4, and concentrated under reduced pressure to yield the crude product **6**, which was purified by silica gel flash column chromatography (7.7 mg, 68%).

^{1}H NMR (400 MHz, $CDCl_3$): δ 7.79 (dd, *J* = 8.0, 1.6 Hz, 1H), 7.72 (d, *J* = 8.0 Hz, 1H), 7.56 (dd, *J* = 7.2, 1.2 Hz, 1H), 7.46 (t, *J* = 7.2 Hz, 1H), 6.70 (s, 1H), 2.60 (t, *J* = 7.6 Hz, 2H), 2.37 (s, 3H), 2.21 (s, 3H), 1.52 (sext, *J* = 7.6 Hz, 2H), 0.87 (t, *J* = 7.6 Hz, 3H);

^{13}C NMR (100 MHz, $CDCl_3$): δ 194.8, 144.2, 140.4, 138.4, 136.7, 135.1, 131.8, 130.4, 128.3, 128.0, 127.1, 37.0, 23.7, 22.7, 21.9, 14.0;

HRMS (ESI): $[M+H]^+$ Calcd for $C_{16}H_{19}O$: 227.1430; found: 227.1423.

c) Procedure for the synthesis of 4,12-dimethyl-10-propyl-3a,4,10,10a-tetrahydro-1*H*-4,10-ethenobenzo[4,5]cyclohepta[1,2-c]furan-1,3,9-trione

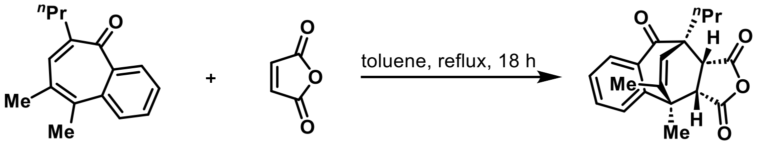

6 7

6 (1.0 equiv, 0.1 mmol, 11.3 mg), maleic anhydride (1.0 equiv, 9.8 mg), and toluene (1 mL) were added into a schlenk tube, and the mixture was stirred for 18 h at 110 ℃. After cooling to room temperature, the reaction was quenched with H_2O, extracted with EtOAc for three times, washed with brine, dried over Na_2SO_4 and then purified by flash column chromatography to afford **7** (10.4 mg, 32%).

^{1}H NMR (400 MHz, $CDCl_3$): δ 8.03 (dd, *J* = 7.6, 1.6 Hz, 1H), 7.52 (ddd, *J* = 8.0, 7.2, 1.6 Hz, 1H), 7.45 (dd, *J* = 8.0, 1.2 Hz, 1H), 7.40 (td, *J* = 7.6, 1.2 Hz, 1H), 5.52 (s, 1H), 3.53 (d, *J* = 8.8 Hz, 1H), 3.46 (d, *J* = 8.8 Hz, 1H), 2.16 (ddd, *J* = 16.0, 12.0, 4.4 Hz, 1H), 2.02 (s, 3H), 1.87 (ddd, *J* = 16.0, 12.0, 4.0 Hz, 1H), 1.81 (d, *J* = 1.6 Hz, 3H), 1.67–1.56 (m, 1H), 1.51–1.39 (m, 1H), 1.00 (t, *J* = 7.2 Hz, 3H);

^{13}C NMR (100 MHz, $CDCl_3$): δ 192.2, 169.4, 168.9, 151.7, 145.9, 133.7, 133.2, 131.9, 128.4, 124.9, 124.7, 55.6, 54.9, 46.8, 44.9, 36.1, 21.1, 20.4, 17.7, 14.8; **HRMS** (ESI): $[M+H]^+$ Calcd for $C_{20}H_{21}O_4$: 325.1434; found: 325.1426.

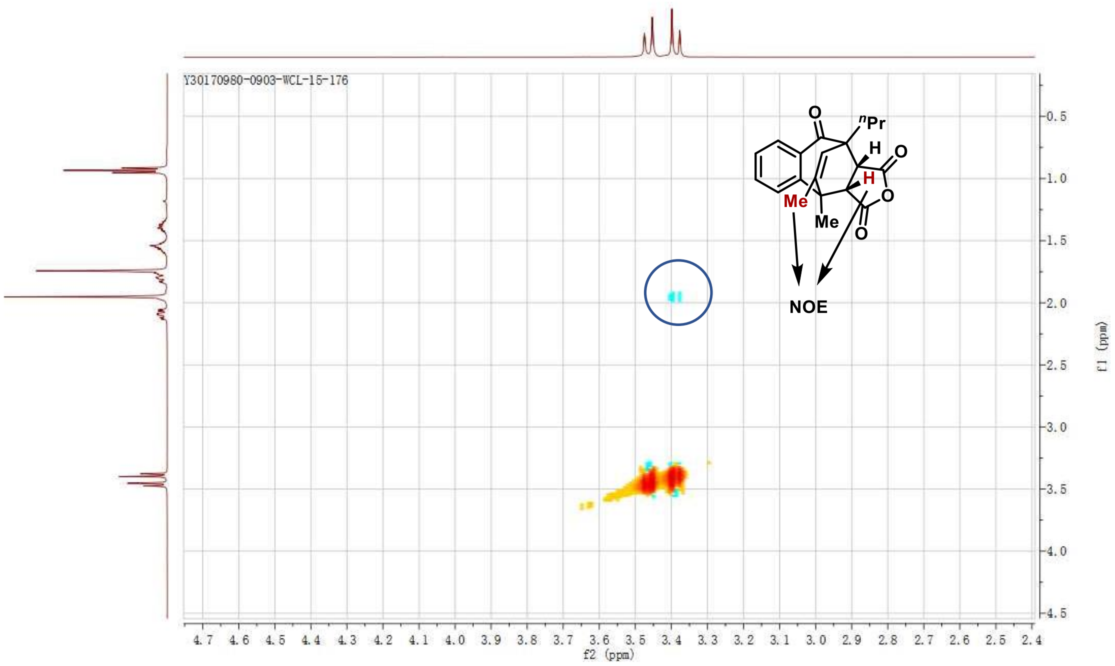

d) Procedure for the synthesis of (*E*)-8,9-dimethyl-3,4,5,6-tetrahydro-1*H*-benzo[6,7]cyclohepta[1,2-b]azocine-2,7-dione

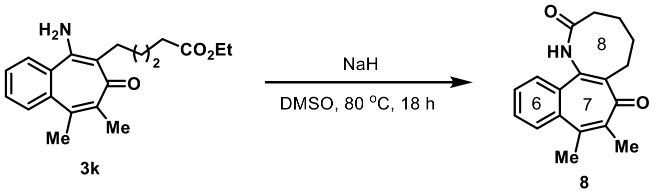

To a solution of NaH (1.1 equiv, 0.055 mmol, 2.2 mg) in DMSO (1 mL) was added **3k** (1.0 equiv, 0.05 mmol, 16.4 mg), and the resulted mixture was heated at 80 ℃ for 24 h. After cooling to room temperature, the reaction was quenched with H_2O, extracted with EtOAc for three times, washed with brine, dried over Na_2SO_4 and then purified by flash column chromatography to afford **8** (5.6 mg, 40%) R*f* = 0.18 (PE/EtOAc = 3/1); **^{1}H NMR** (400 MHz, $CDCl_3$): δ 7.67 (ddd, *J* = 9.6, 8.4, 1.2 Hz, 2H), 7.45 (td, *J* = 7.2, 1.2 Hz, 1H), 7.39 (td, *J* = 8.0, 1.6 Hz, 1H), 6.94 (bs, 1H), 2.65–2.55 (m, 2H), 2.42–2.37 (m, 1H), 2.37 (q, *J* = 0.8 Hz, 3H), 2.25–2.20 (m, 1H), 2.20 (q, *J* = 0.8 Hz, 3H), 2.13–2.08 (m, 1H), 2.03–2.01 (m, 2H), 1.70–1.67 (m, 1H); **^{13}C NMR** (100 MHz, $CDCl_3$): δ 200.0, 176.4, 141.1, 139.7, 138.9, 137.7, 134.2, 132.3, 128.6, 128.2, 127.6, 127.4, 33.0, 30.7, 26.8, 24.1, 21.4, 18.5; **HRMS** (ESI): $[M+H]^+$ Calcd for $C_{18}H_{20}NO_2$: 282.1489; found: 282.1482.

e) Procedure for the synthesis of spirocompound 11

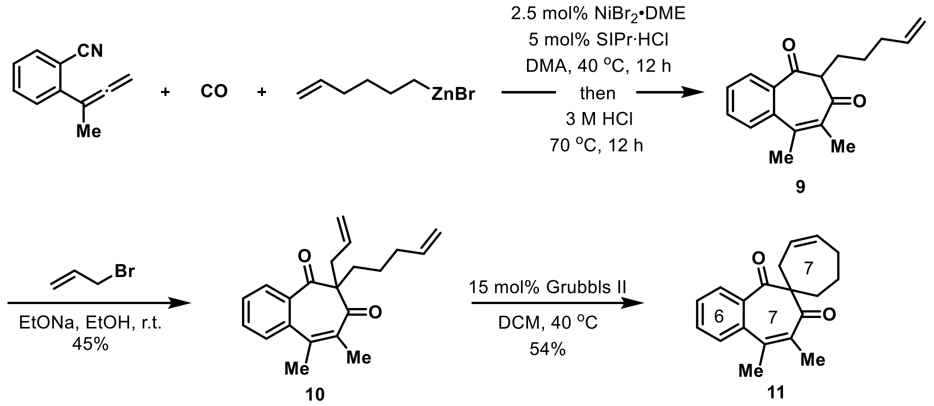

A 20 mL oven-dried tube charged with $NiBr_2$·DME (2.5 mol%, 2.4 mg) and SIPr·HCl (5 mol%, 6.6 mg) was evacuated and backfilled with N_2 three times. The reaction mixture was evacuated again and backfilled with CO (1 atm, balloon), followed by addition of DMA (0.1 M, 3 mL), **1a** (0.3 mmol, 46.5 mg) and organiczinc (1.5 equiv, 0.45 mmol, 0.68 mL, 0.66 M in THF) at r.t. The tube was screw-capped and the reaction mixture was allowed to stir at 40 ^{o}C oil bath for 12 h. The mixture was quenched with 3 M HCl (6 mL) and stirred for 12 h at 70 ^{o}C. After cooling to r.t., The mixture was neutralized with saturated $NaHCO_3$ and extracted with EtOAc. The separated organic layer was washed with brine, dried over anhydrous Na_2SO_4, and concentrated under reduced pressure to yield the crude product **9**, which was purified by silica gel flash column chromatography (40.3 mg, 50%); R*f* = 0.43 (PE/EtOAc = 20/1); ^{1}**H NMR** (400 MHz, $CDCl_3$): δ 7.48 (ddd, *J* = 8.0, 6.8, 2.0 Hz, 1H), 7.35–7.28 (m, 1H), 5.76 (ddt, *J* = 16.8, 10.0, 6.4 Hz, 1H), 5.00 (dq, *J* = 16.8, 1.6 Hz, 1H), 4.97–4.93 (m, 1H), 3.70 (t, *J* = 6.8 Hz, 1H), 2.17 (q, *J* = 0.8 Hz, 3H), 2.06 (q, *J* = 7.2 Hz, 2H), 1.99 (q, *J* = 0.8 Hz, 3H), 1.96–1.86 (m, 2H), 1.45–1.36 (m, 2H); ^{13}**C NMR** (100 MHz, $CDCl_3$): δ 203.1, 202.7, 138.1, 138.1, 137.9, 137.4, 135.9, 131.6, 128.1, 127.8, 126.9, 115.2, 71.6, 33.6, 27.2, 26.4, 20.7, 17.2; **HRMS** (ESI): $[M+H]^+$ Calcd for $C_{18}H_{21}O_2$: 269.1536; found: 269.1528.

A 10 mL oven-dried tube charged with **9** (1.0 equiv, 0.12 mmol, 32.2 mg), NaOEt (2.0 equiv, 0.24 mmol, 16.3 mg), EtOH (1 mL). After stirring at room temperature for 20 min, the allyl bromide (2.0 equiv, 0.24 mmol, 20.8 uL) was added and then stirring at room temperature for 12 h. The reaction was quenched with H_2O, extracted with EtOAc for three times, washed with brine, dried over Na_2SO_4 and then purified by flash column chromatography to afford **10** (16.7 mg, 45%); R*f* = 0.53 (PE/EtOAc = 20/1); ^{1}**H NMR** (400 MHz, $CDCl_3$): δ 7.42 (td, *J* = 7.6, 1.6 Hz, 1H), 7.28–7.24 (m, 2H), 7.19 (dd, *J* = 7.6, 1.2 Hz, 1H), 5.76 (ddt, *J* = 16.8, 10.0, 6.8 Hz, 1H), 5.76 (ddt, *J* = 17.2, 9.6, 7.2 Hz, 1H), 5.10–5.06 (m, 2H), 4.93–5.01 (m, 2H), 2.56 (abqd, *J* = 14.8, 7.2 Hz, 2H), 2.09 (q, *J* = 0.8 Hz, 3H), 2.01 (q, *J* = 7.2 Hz, 2H), 1.95 (q, *J* = 0.8 Hz, 3H), 1.89–1.77 (m, 2H), 1.27–1.18 (m, 2H); ^{13}**C NMR** (100 MHz, $CDCl_3$): δ 206.4, 204.9, 138.7, 138.1, 137.6, 136.5, 135.9, 132.2, 131.2, 127.7, 127.1, 127.0, 119.1, 115.3, 75.3, 35.4, 34.0, 30.5, 22.8, 19.8, 17.5; **HRMS** (ESI): $[M+H]^+$ Calcd for $C_{21}H_{25}O_2$: 309.1849; found: 309.1840.

10 (1.0 equiv, 0.04 mmol, 12.3 mg), Grubbls II catalyst (15 mol%, 3.8 mg), and DCE (1 mL) were added into a 10 ml schlenk tube, and the mixture was stirred for 24 h at 40 ℃. After cooling to room temperature, the reaction was quenched with H_2O, extracted with DCM for three times, washed with brine, dried over Na_2SO_4 and then purified by flash column chromatography to afford **11** (6.1 mg, 54%); R*f* = 0.42 (PE/EtOAc = 20/1); ^{1}**H NMR** (400 MHz, $CDCl_3$): δ 7.42 (ddd, *J* = 8.4, 7.6, 1.6 Hz, 1H), 7.28–7.24

(m, 2H), 7.20 (dd, *J* = 8.0, 1.6 Hz, 1H), 5.81 (td, *J* = 10.8, 5.6 Hz, 1H), 5.55 (dtt, *J* = 10.4, 6.8, 1.2 Hz, 1H), 2.61 (abqd, *J* = 14.8, 6.0 Hz, 2H), 2.26–2.21 (m, 2H), 2.13 (sext, *J* = 4.0 Hz, 2H), 2.09 (q, *J* = 1.2 Hz, 3H), 1.98 (q, *J* = 1.2 Hz, 3H), 1.77–1.66 (m, 2H); **^{13}C NMR** (100 MHz, $CDCl_3$): δ 206.9, 205.9, 138.4, 137.5, 136.2, 135.1, 133.5, 130.9, 127.6, 127.0, 126.9, 125.1, 73.9, 32.2, 29.7, 29.2, 21.0, 19.4, 17.9; **HRMS** (ESI): $[M+H]^+$ Calcd for $C_{19}H_{21}O_2$: 281.1536; found: 281.1528.

Mechanistic studies

a) The effect of cyano group

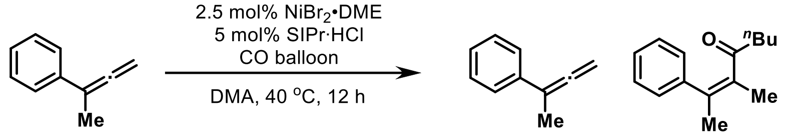

1n | **1n**, 30% | **4n**, 22% Z/E = 6/1

A 10 mL sealed tube charged with $NiBr_2$·DME (2.5 mol%, 0.8 mg) and SIPr·HCl (5 mol%, 2.2 mg) was evacuated and backfilled with N_2 three times. The reaction mixture was evacuated againand backfilled with CO (1 atm, balloon), followed by addition of DMA (1 mL), **1n** (1.0 equiv, 0.1 mmol, 13.0 mg) and ^{n}BuZnCl (1.5 equiv, 0.15 mmol, 0.37 mL, 0.42 M in THF) at r.t. The tube was screw-capped and reaction mixture was allowed to warm to 40 °C oil bath for 12 h. The mixture was quenched with saturated aqueous NH_4Cl and extracted with EtOAc. The separated organic layer was washed with brine, dried over Na_2SO_4 and then purified by flash column chromatography to afford **1n** (3.9 mg, 30%), **4n**[3] (4.8 mg, 22%, *Z*/*E* = 6/1, **^{1}H NMR** (400 MHz, $CDCl_3$): δ 7.36–7.31 (m, 1H), 7.30–7.27 (m, 2H), 7.15–7.13 (m, 2H), 2.10 (q, *J* = 1.2 Hz, 3H), 1.94 (q, *J* = 1.2 Hz, 3H), 1.89 (t, *J* = 7.2 Hz, 2H), 1.27 (quint, *J* = 7.6 Hz, 2H), 0.99 (sext, *J* = 7.6 Hz, 2H), 0.68 (t, *J* = 7.2 Hz, 3H)). This result indicated that the cyano group not only served as an electrophilic component and significantly promoted the reaction efficiency, but also acted as a directing group to improve the stereo- and regioselectivity of this reaction.

b) Real-time tracing experiment

A 10 mL sealed tube charged with $NiBr_2$·DME (2.5 mol%, 0.4 mg) and SIPr·HCl (5 mol%, 1.1 mg) was evacuated and backfilled with N_2 three times. The reaction mixture was evacuated againand backfilled with CO (1 atm, balloon), followed by addition of DMA (0.5 mL), **1a** (1.0 equiv, 0.05 mmol, 7.8 mg) and ^{n}BuZnCl (1.5 equiv, 0.075 mmol, 0.18 mL, 0.41 M in THF) at r.t. The tube was screw-capped and reaction mixture was allowed to stir at 40 °C. The mixture was quenched with saturated aqueous NH_4Cl and extracted with EtOAc. The separated organic layer was washed with brine, dried over anhydrous Na_2SO_4, and concentrated under reduced pressure to yield the crude product, which was determined by ^{1}H NMR using CH_2Br_2 as the internal standard. Only a small amount of **4a** was observed within the first 30 minutes of the reaction, and no product could be observed. With prolonging the reaction time, the concentration of benzotropone **3a** began to increase with the consumption of both **1a** and **4a**. The starting material **1a** was completely consumed within 5 h, while the remaining **4a** was fully transformed into product **3a** in about 10 h. The above results indicated that compound **4a** might be an intermediate of this reaction.

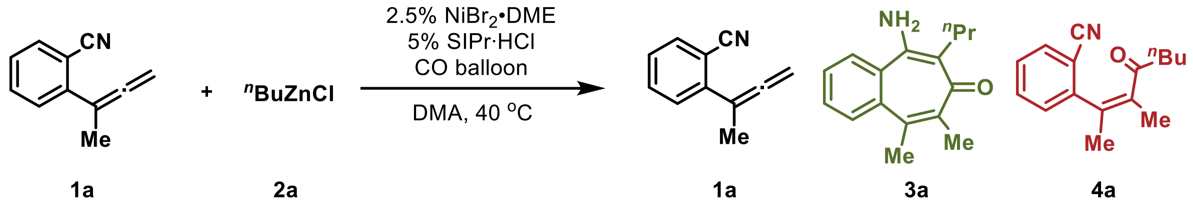

Entry	Time	Conc. (mol/L)		
		1a	3a	4a
1	0.17 h	0.062	0	0.007
2	0.5 h	0.045	0	0.020
3	1 h	0.031	0.003	0.024
4	2 h	0.024	0.015	0.022
5	3 h	0.015	0.024	0.021
6	4 h	0.010	0.033	0.018
7	5 h	0	0.043	0.016
8	6 h	0	0.047	0.013
9	8 h	0	0.051	0.006
10	10 h	0	0.055	0.002

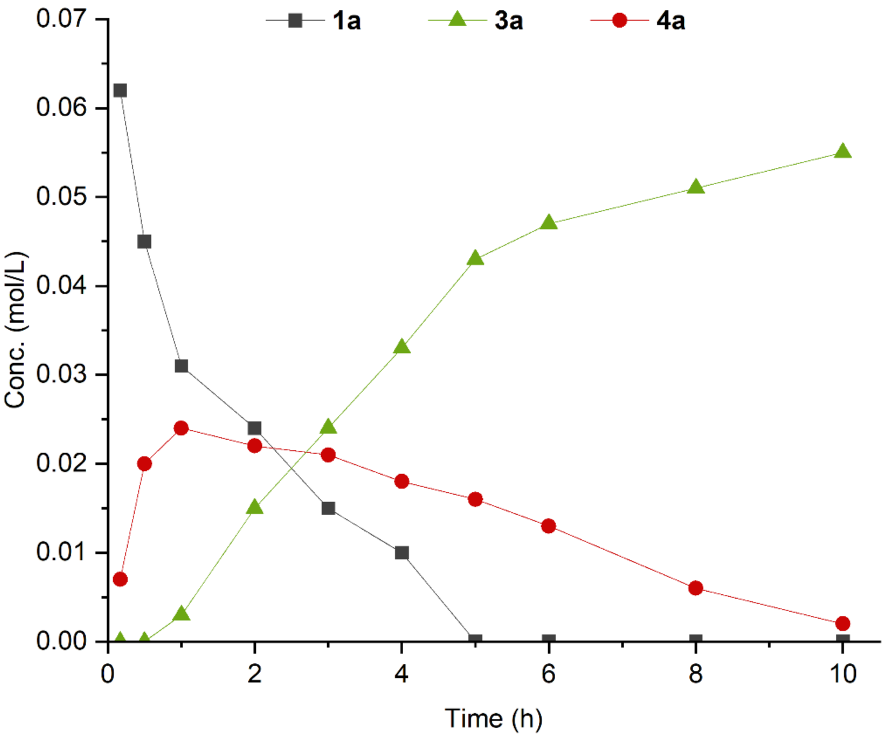

Supplementary Figure 1. Time-course experiment of acylzincation/cyclization reaction

c) Control experiments

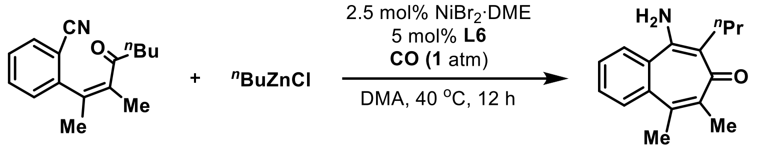

4a 2a 3a

wihtout ^{n}BuZnCl — no reaction

without $NiBr_2$•DME and L_6 — 72% yield

A 10 mL sealed tube charged with $NiBr_2$·DME (2.5 mol%) and SIPr·HCl (5 mol%) was evacuated and backfilled with N_2 three times. The reaction mixture was evacuated againand backfilled with CO (1 atm, balloon), followed by addition of DMA (0.1 M), **4a** (1.0 equiv) at r.t. The tube was screw-capped and reaction mixture was allowed to warm to 40 °C oil bath for 12 h. The desired product **3a** was not obeserved. Similarly, when the control experiments was performed in the absence of nickel catalyst and ligand **L6**, **3a** was obtained in 72% yield. These combined results demonstrated that organozinc reagent was necessitated for the cyclization process of intermediate **4a**.

d) Deuterium zinc reagent experiment

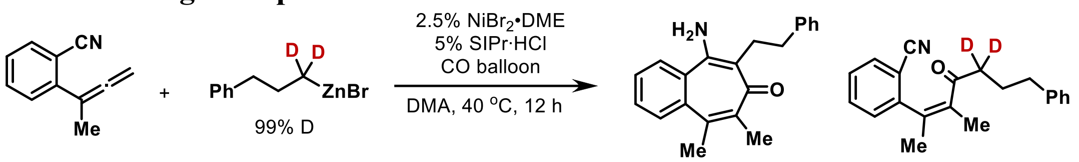

1a **2b'** **3b**, 36%, 0% D **4b'**, 20%, 99% D

A 10 mL sealed tube charged with $NiBr_2$·DME (2.5 mol%, 0.4 mg) and SIPr·HCl (5 mol%, 1.1 mg) was evacuated and backfilled with N_2 three times. The reaction mixture was evacuated againand backfilled with CO (1 atm, balloon), followed by addition of DMA (0.5 mL), **1a** (1.0 equiv, 0.05 mmol, 7.8 mg) and **2b'** (1.5 equiv, 0.075 mmol, 0.17 mL, 0.45 M in THF) at r.t. The tube was screw-capped and reaction mixture was allowed to stir at 40 °C for 12 h. The mixture was quenched with saturated aqueous NH_4Cl and extracted with EtOAc. The separated organic layer was washed with brine, dried over Na_2SO_4 and then purified by flash column chromatography to afford **3b** (5.5 mg, 36%), **4b'** (3.1 mg, 20%, *Z/E* = 12/1), **^{1}H NMR** (400 MHz, $CDCl_3$): δ 7.60 (dd, *J* = 7.6, 1.2 Hz, 1H), 7.49 (td, *J* = 7.6, 1.2 Hz, 1H), 7.32 (td, *J* = 7.6, 1.2 Hz, 1H), 7.24–7.21 (m, 2H), 7.18–7.13 (m, 2H), 7.05–7.03 (m, 2H), 2.39 (t, *J* = 7.6 Hz, 2H), 2.11 (q, *J* = 0.8 Hz, 3H), 2.01 (q, *J* = 0.8 Hz, 3H), 1.66 (t, *J* = 7.2 Hz, 2H).

e) Deuterium reagents trap experiment

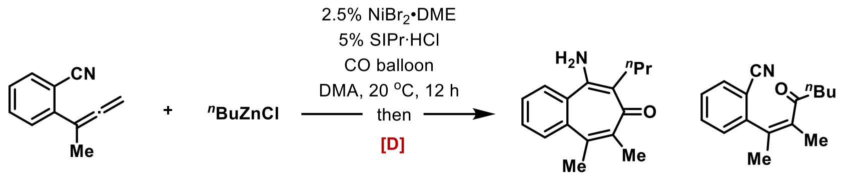

1a **2a** **3a** **4a**

Entry	[D]	Yield (%)	
		3a	4a
1	D_2O, r.t. 3 h	30 (0% D)	23 (0% D)
2	CH_3OD, r.t. 3 h	27 (0% D)	22 (0% D)
3	CH_3CO_2D, r.t. 0.5 h	28 (0% D)	19 (0% D)

A 10 mL sealed tube charged with $NiBr_2$·DME (2.5 mol%, 0.4 mg) and SIPr·HCl (5 mol%, 1.1 mg) was evacuated and backfilled with N_2 three times. The reaction mixture was evacuated againand backfilled with CO (1 atm, balloon), followed by addition of DMA (0.5 mL), **1a** (1.0 equiv, 0.05 mmol, 7.8 mg) and ^{n}BuZnCl (1.5 equiv, 0.075 mmol, 0.18 mL, 0.41 M in THF) at r.t. The tube was screw-capped and reaction mixture was allowed to stir at 40 °C for 12 h. The mixture was quenched with saturated aqueous NH_4Cl and extracted with EtOAc. The separated organic layer was washed with brine, dried over Na_2SO_4 and then purified by flash column chromatography to afford **3a** and **4a**.

f) Deuterium solvents experiment

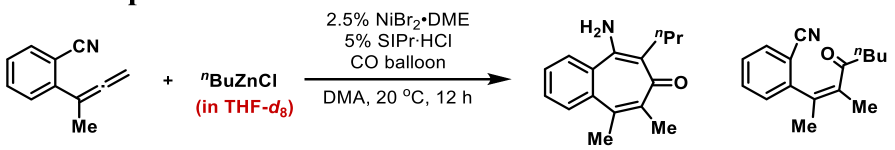

1a **2a'** **3a**, 24%, 0% D **4a**, 21%, 0% D

A 10 mL sealed tube charged with $NiBr_2$·DME (2.5 mol%, 0.4 mg) and SIPr·HCl (5 mol%, 1.1 mg) was evacuated and backfilled with N_2 three times. The reaction mixture was evacuated againand backfilled with CO (1 atm, balloon), followed by addition of DMA (0.5 mL), **1a** (1.0 equiv, 0.05 mmol, 7.8 mg) and ^{n}BuZnCl (1.5 equiv, 0.075 mmol, 0.25 mL, 0.3 M in THF-d_8) at r.t. The tube was screw-capped and reaction mixture was allowed to stir at 40 °C for 12 h. The mixture was quenched with saturated aqueous NH_4Cl and extracted with EtOAc. The separated organic layer was washed with brine, dried over Na_2SO_4 and then purified by flash column chromatography to afford **3a** (2.9 mg, 24%) and **4a** (2.5 mg, 21%).

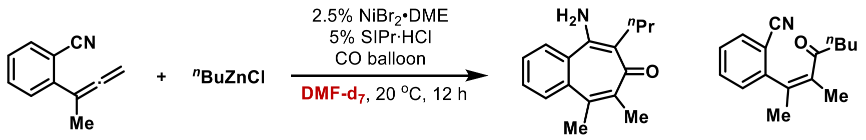

1a **2a** **3a**, 21%, 0% D **4a**, 22%, 0% D

A 10 mL sealed tube charged with $NiBr_2$·DME (2.5 mol%, 0.4 mg) and SIPr·HCl (5 mol%, 1.1 mg) was evacuated and backfilled with N_2 three times. The reaction mixture was evacuated againand backfilled with CO (1 atm, balloon), followed by addition of DMF-d_7 (0.5 mL), **1a** (1.0 equiv, 0.05 mmol,

7.8 mg) and ^{n}BuZnCl (1.5 equiv, 0.075 mmol, 0.18 mL, 0.41 M in THF) at r.t. The tube was screw-capped and reaction mixture was allowed to stir at 40 ^{o}C for 12 h. The mixture was quenched with saturated aqueous NH_4Cl and extracted with EtOAc. The separated organic layer was washed with brine, dried over Na_2SO_4 and then purified by flash column chromatography to afford **3a** (2.5 mg, 21%) and **4a** (2.6 mg, 22%).

In conclusion: The above deuterium experiments ruled out the possibility for the intramolecular metal shift, and the zinc intermediate was trapped in the reaction mixture prior to the aqueous work-up procedure. At the current stage, we are not aware of the exact proton source to quench the zinc intermediate **D**. We envisioned that the basic zinc intermediate might be trapped with DMA, unfortunately, we cannot afford the anhydrous DMA-d_9 as solvent.

NMR spectra

^{1}H NMR-spectrum (400 MHz, $CDCl_3$) of **1b**

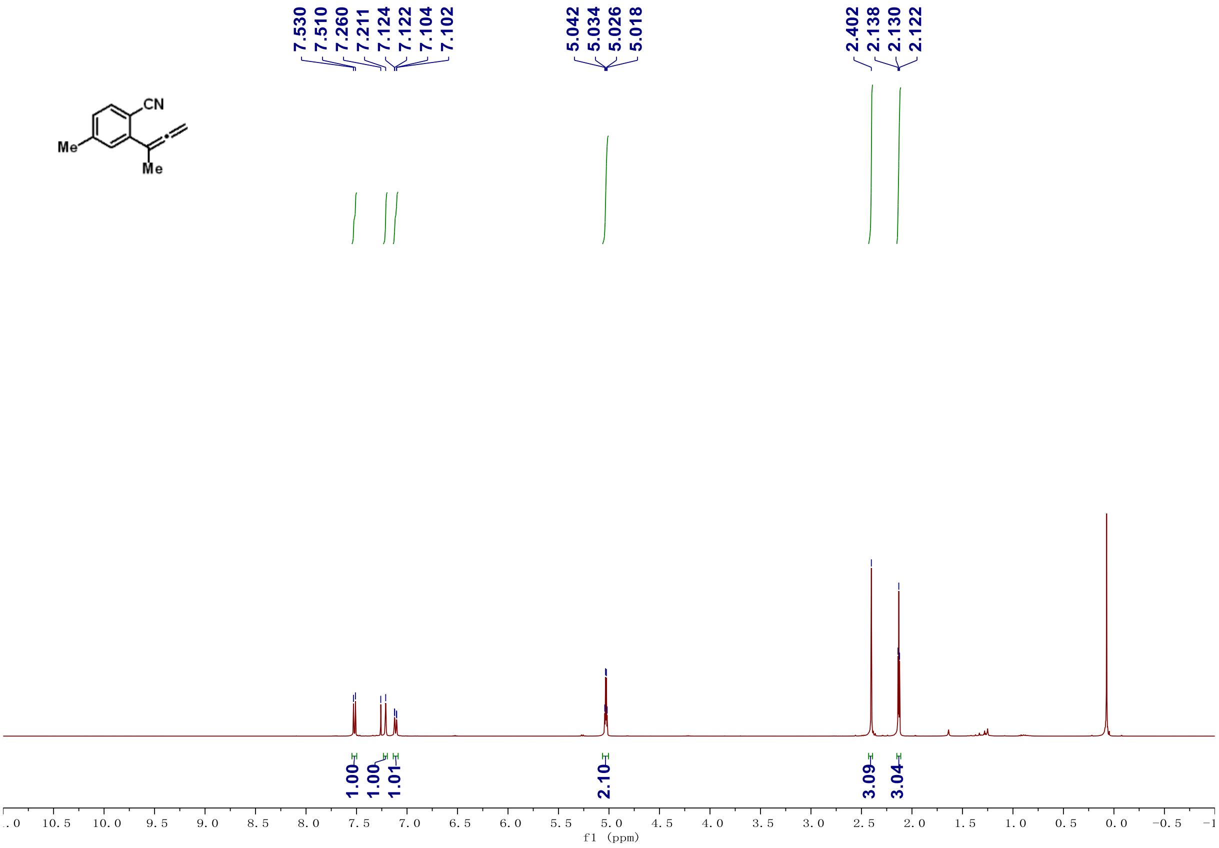

^{13}C NMR-spectrum (100 MHz, $CDCl_3$) of **1b**

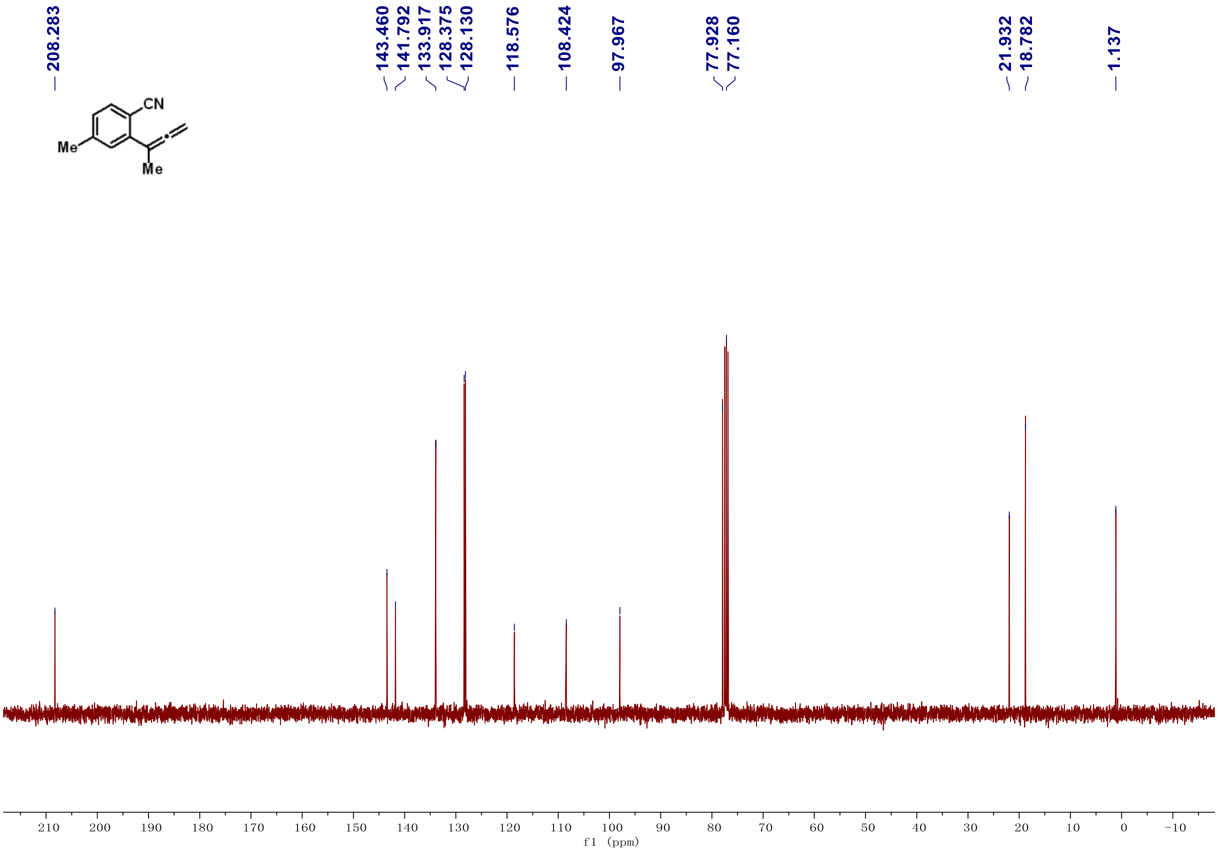

^{1}H NMR-spectrum (400 MHz, $CDCl_3$) of **1c**

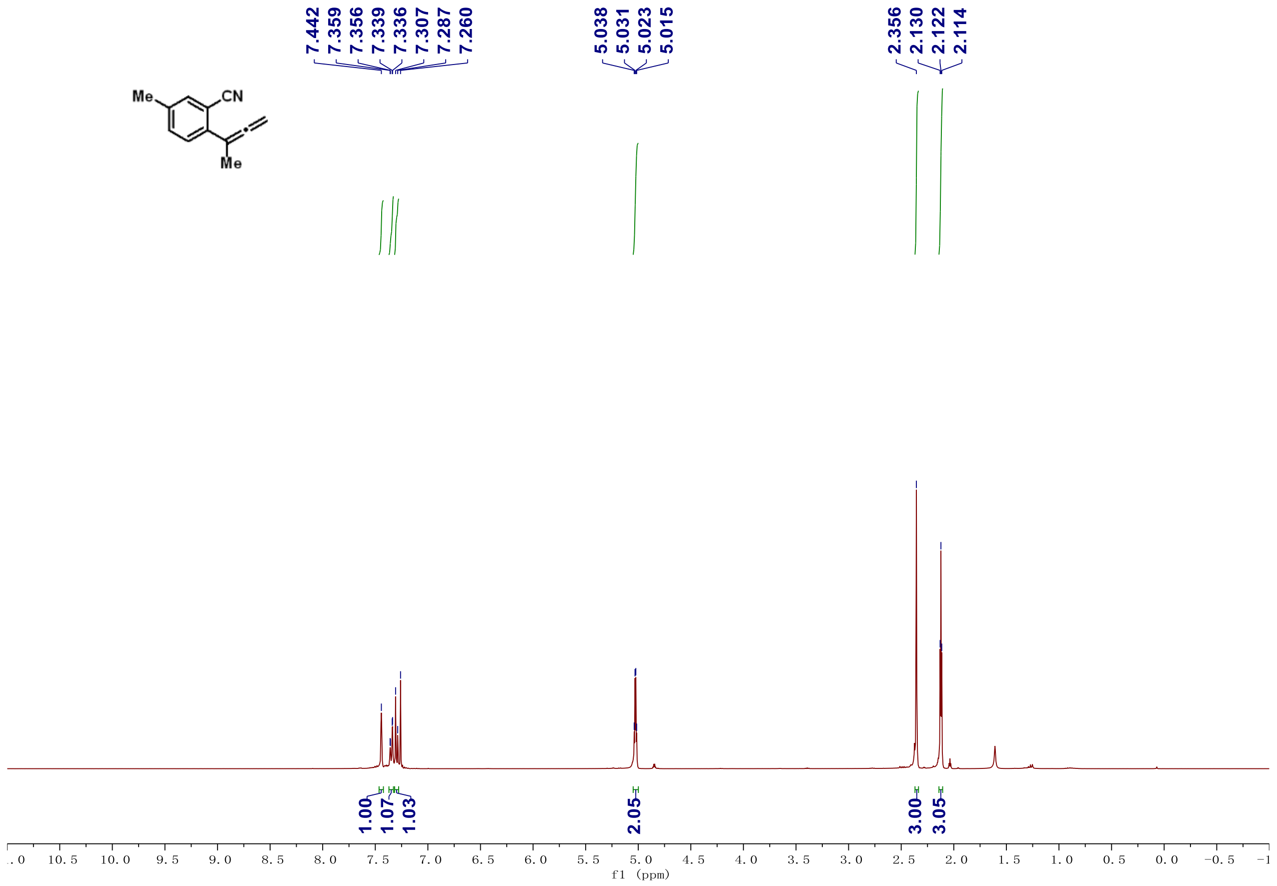

^{13}C NMR-spectrum (100 MHz, $CDCl_3$) of **1c**

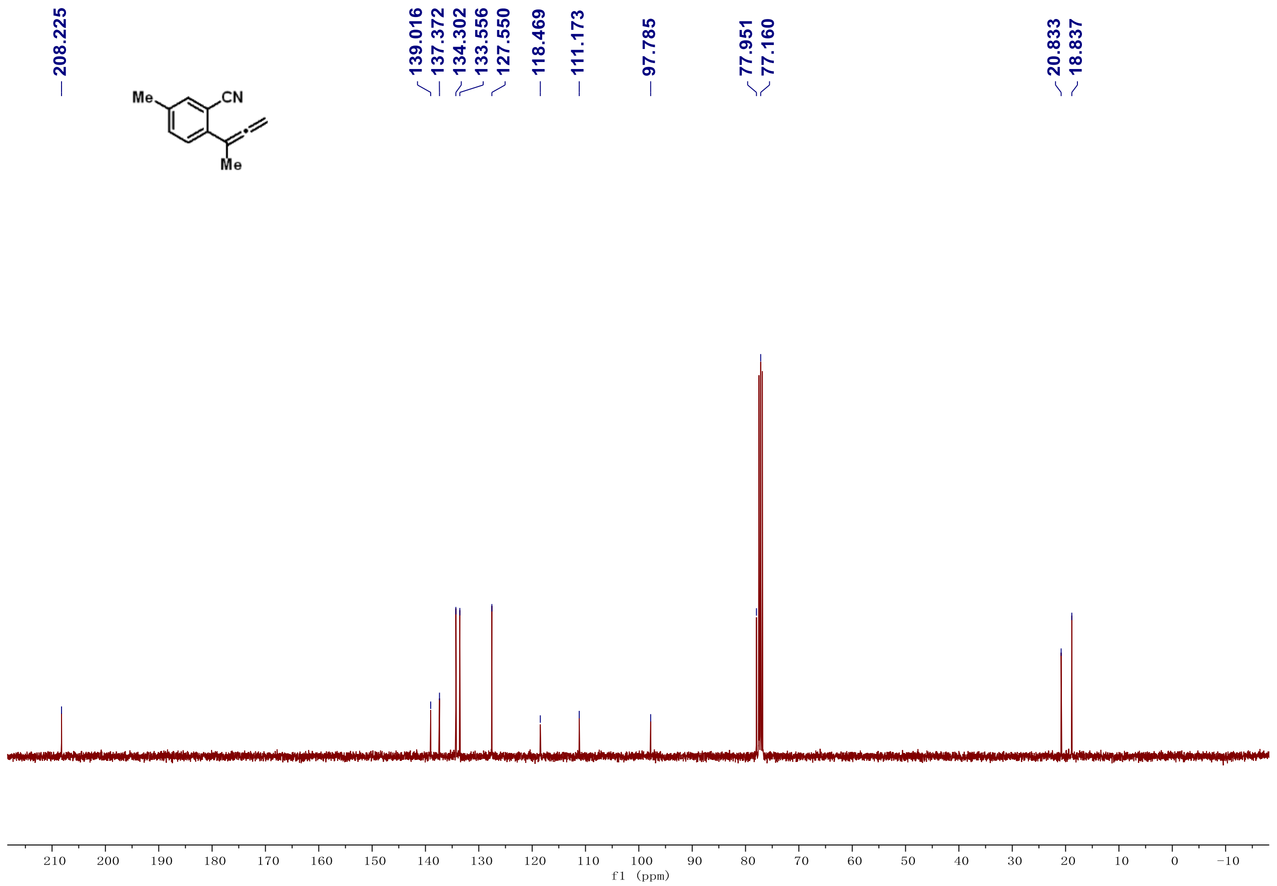

^{1}H NMR-spectrum (400 MHz, $CDCl_3$) of **1d**

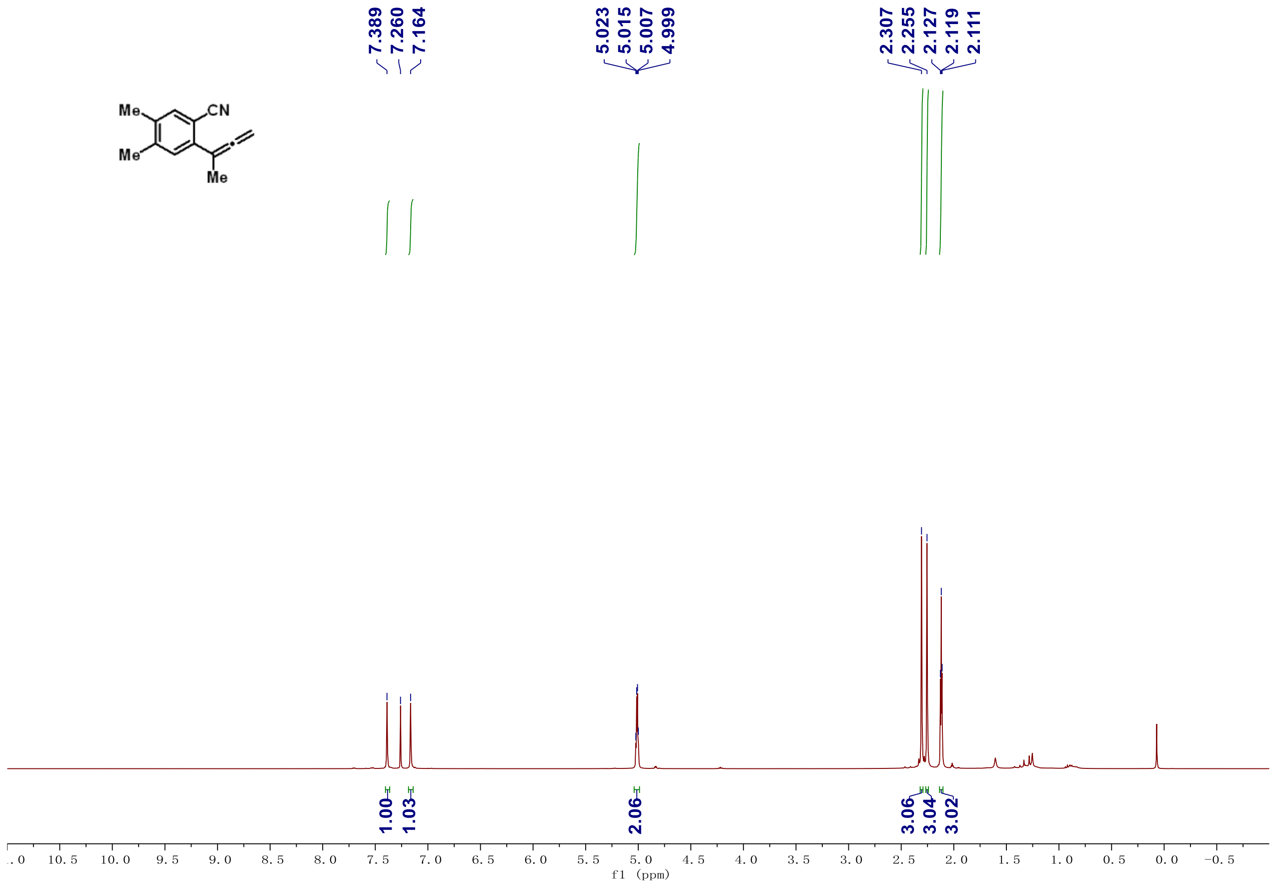

^{13}C NMR-spectrum (100 MHz, $CDCl_3$) of **1d**

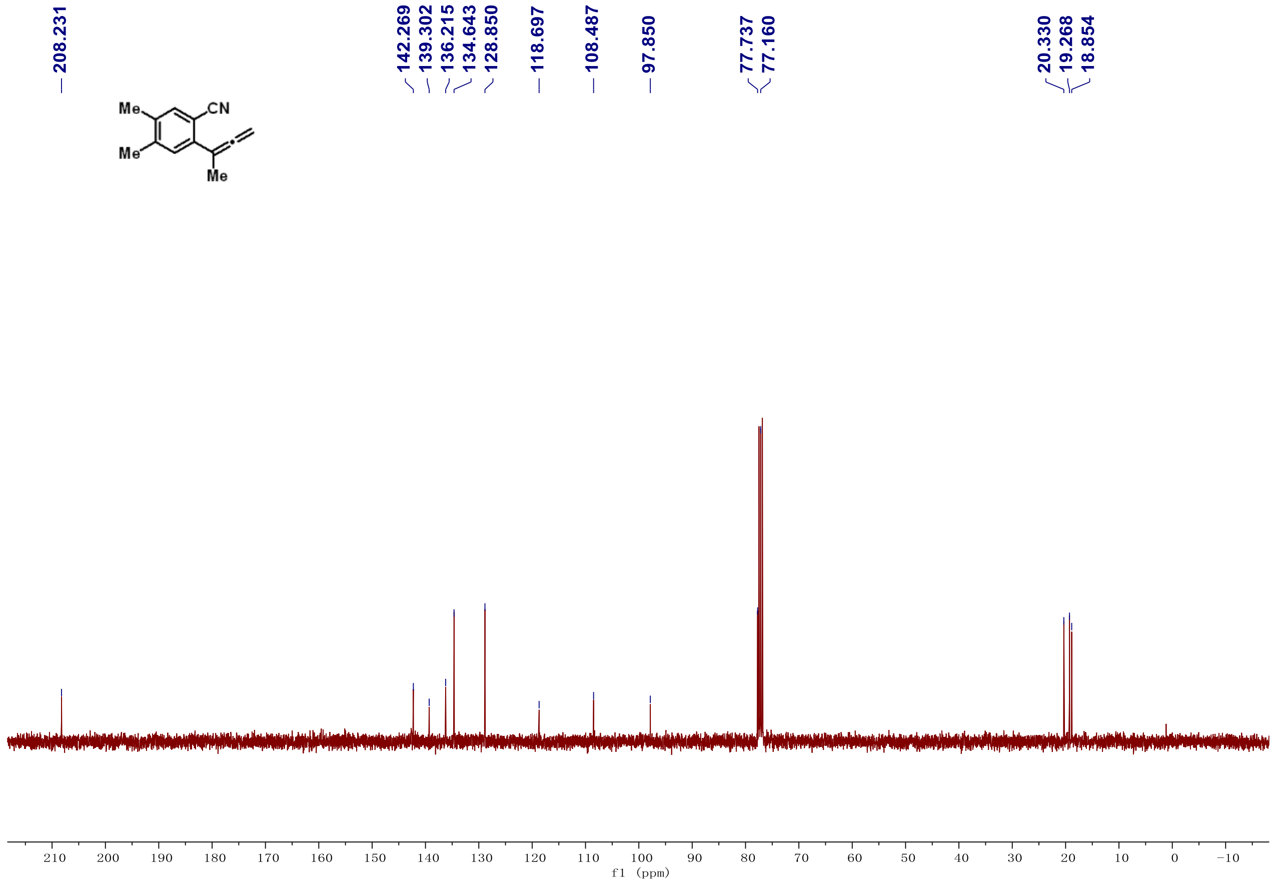

^{1}H NMR-spectrum (400 MHz, $CDCl_3$) of **1e**

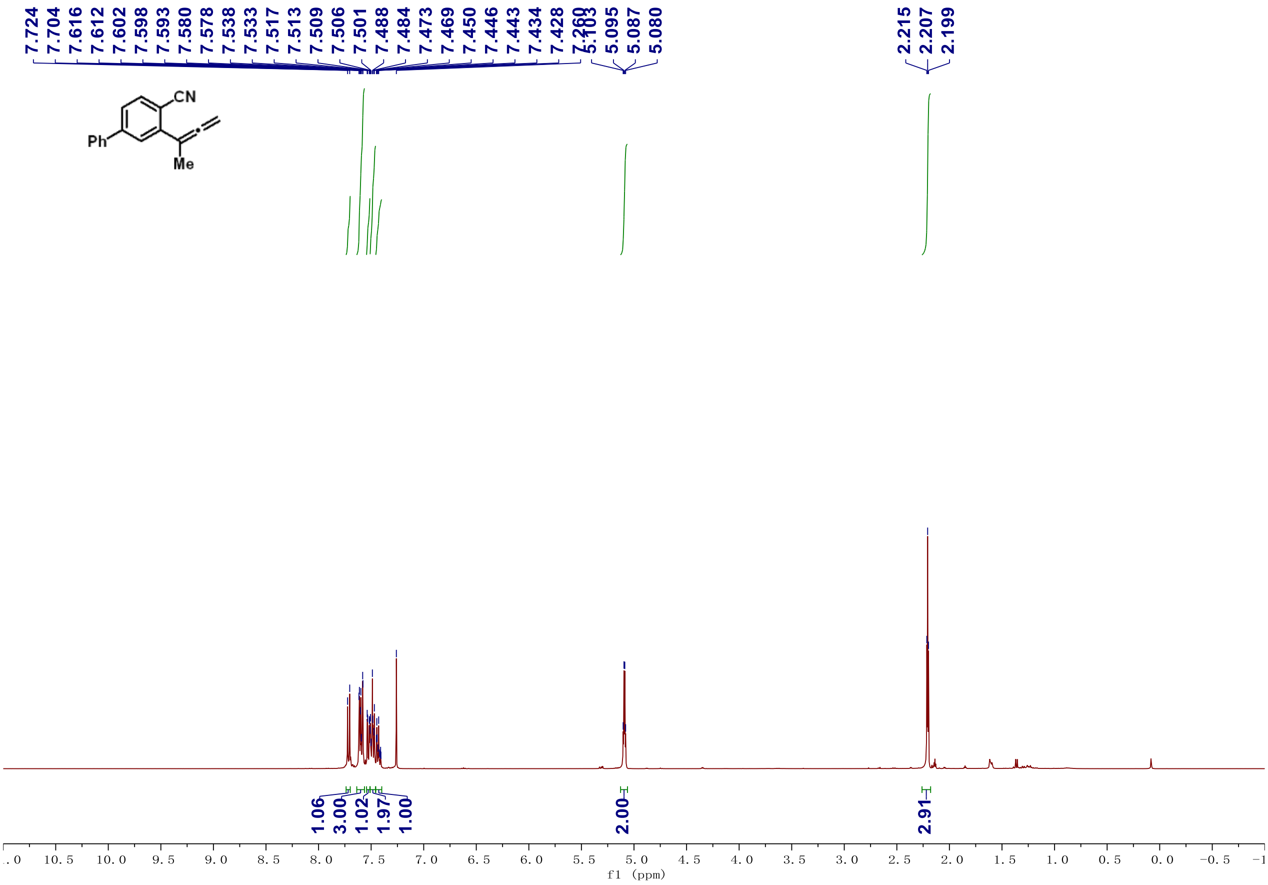

^{13}C NMR-spectrum (100 MHz, $CDCl_3$) of **1e**

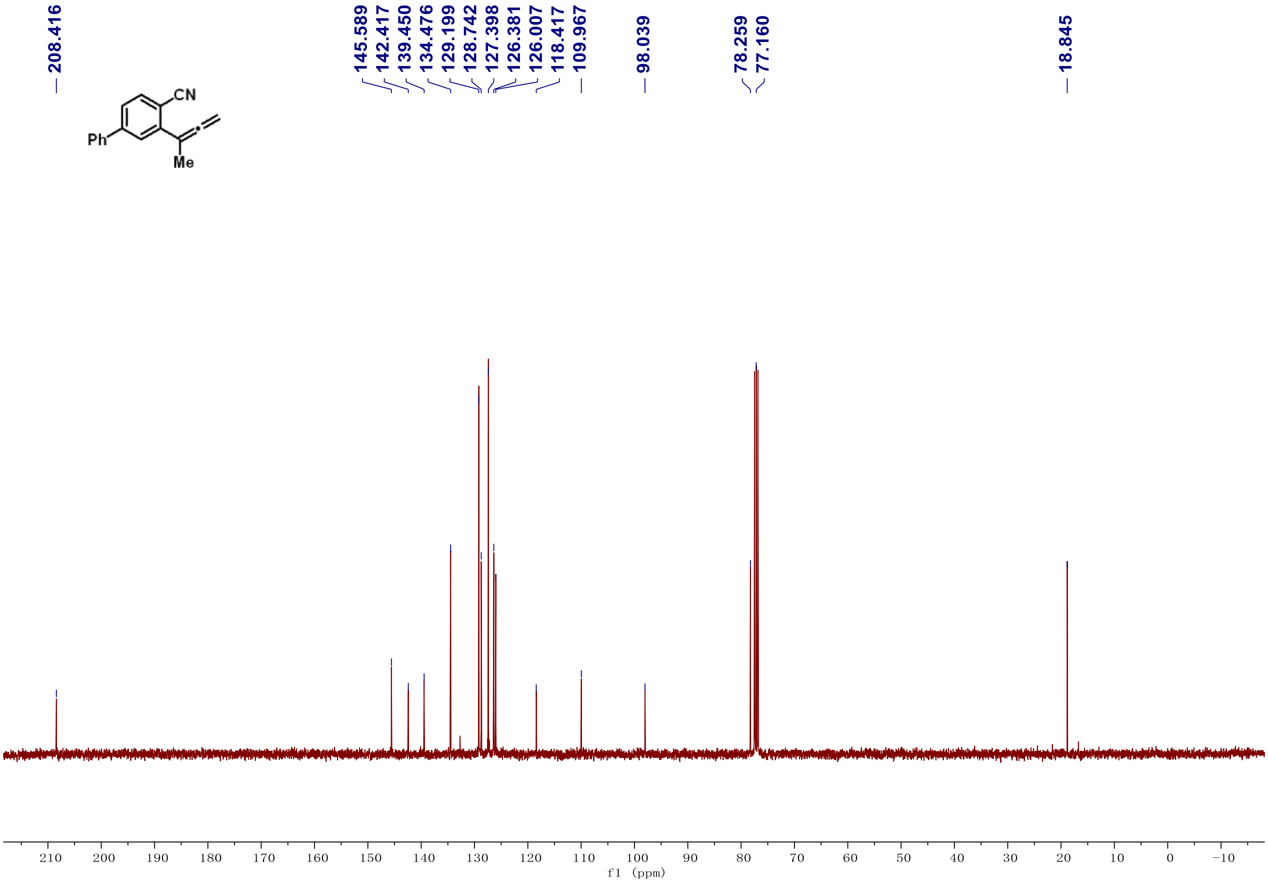

^{1}H NMR-spectrum (400 MHz, $CDCl_3$) of **1f**

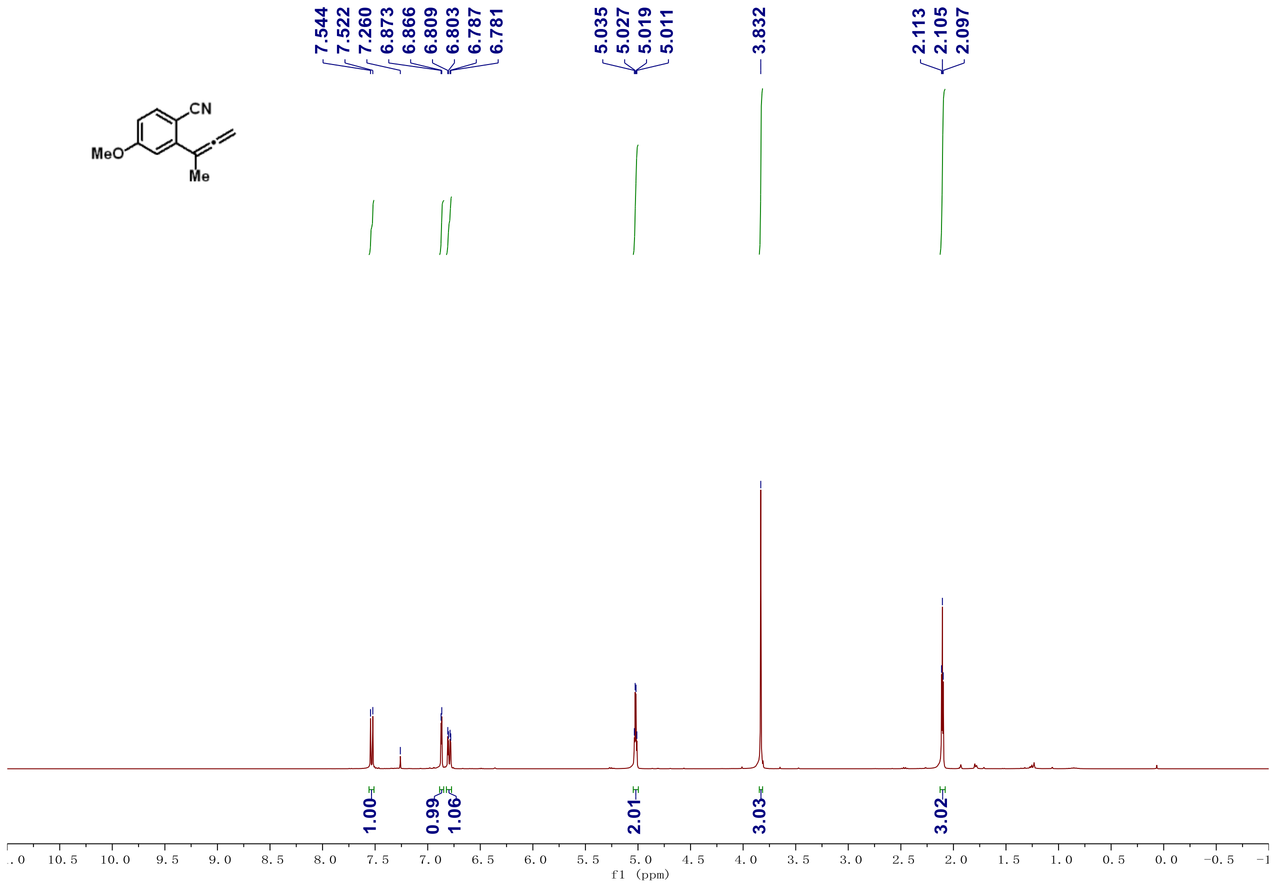

^{13}C NMR-spectrum (100 MHz, $CDCl_3$) of **1f**

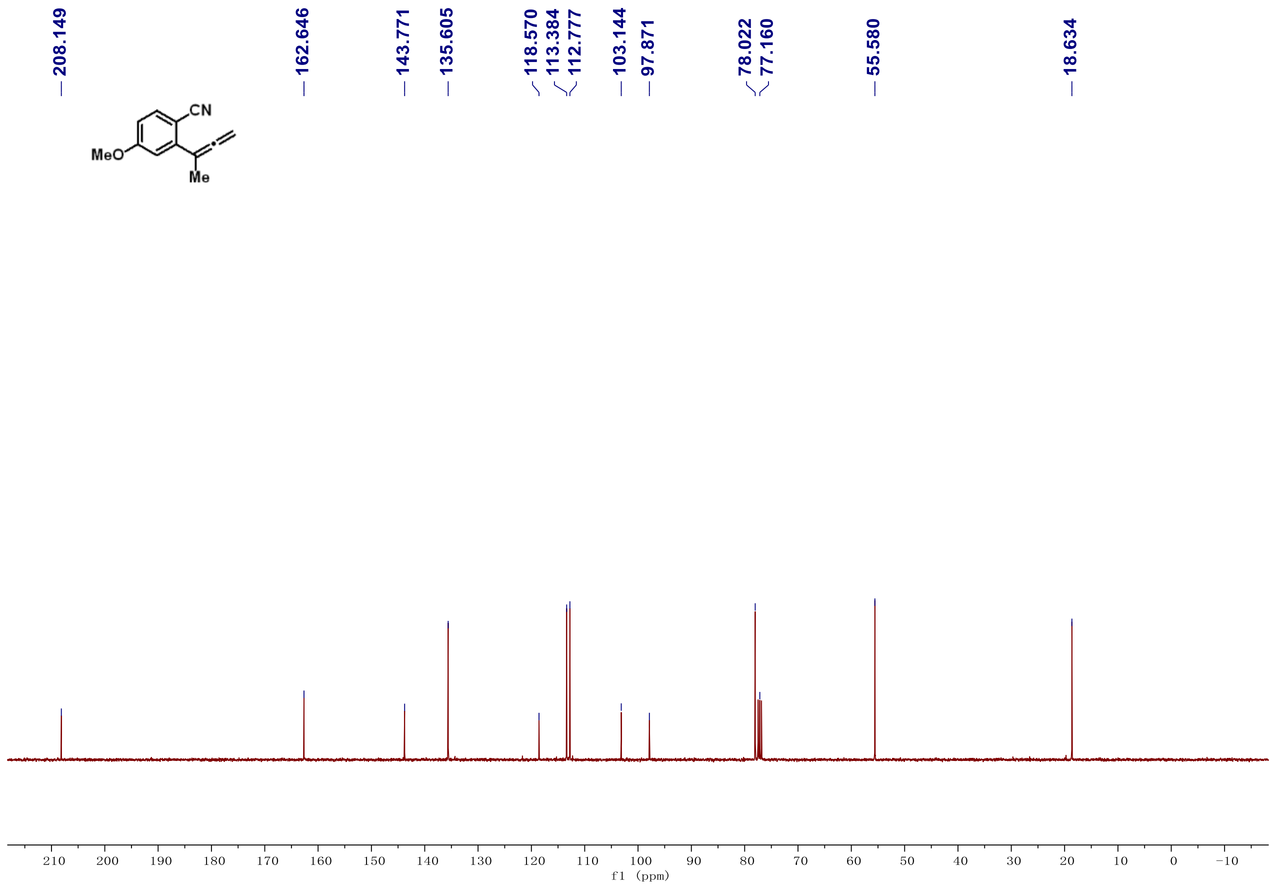

^{1}H NMR-spectrum (400 MHz, $CDCl_3$) of **1g**

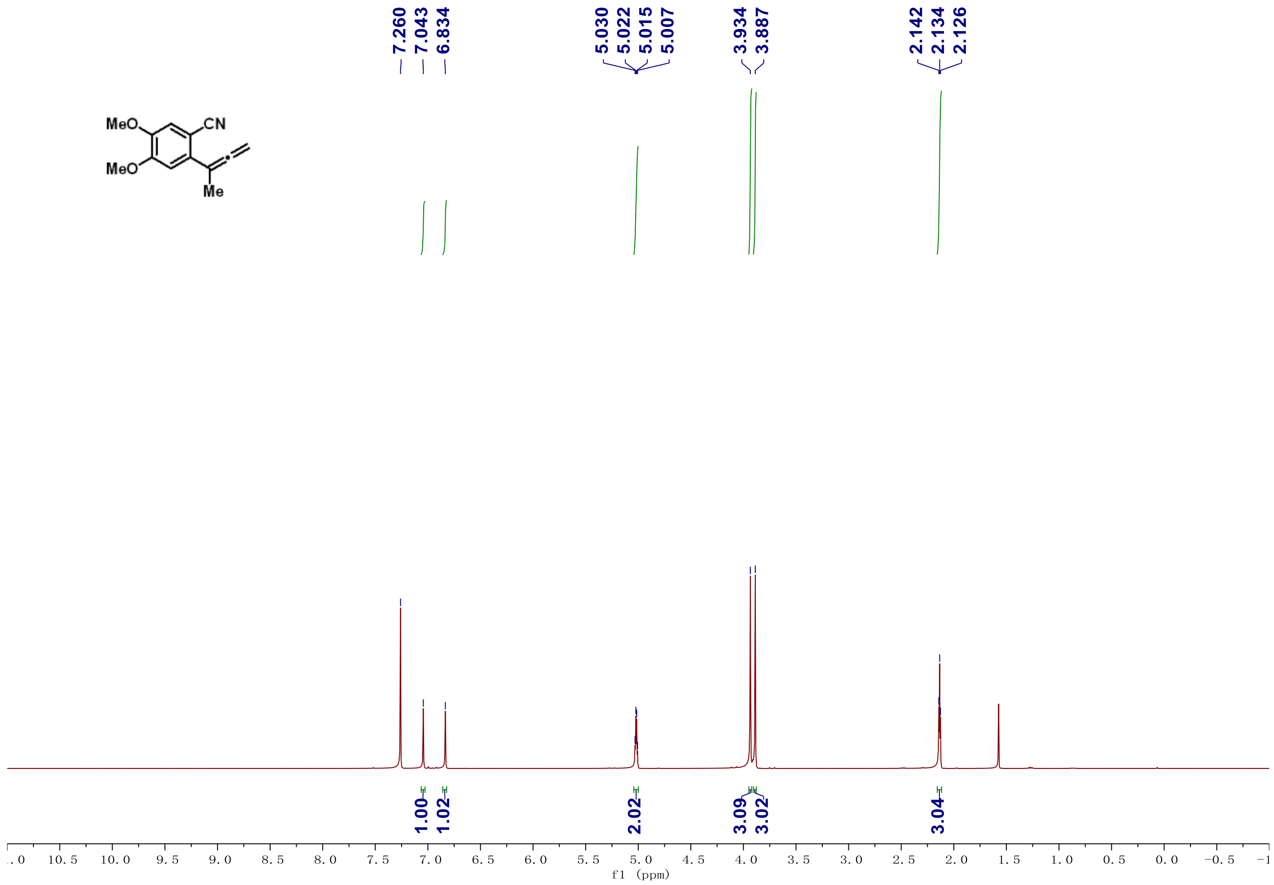

^{13}C NMR-spectrum (100 MHz, $CDCl_3$) of **1g**

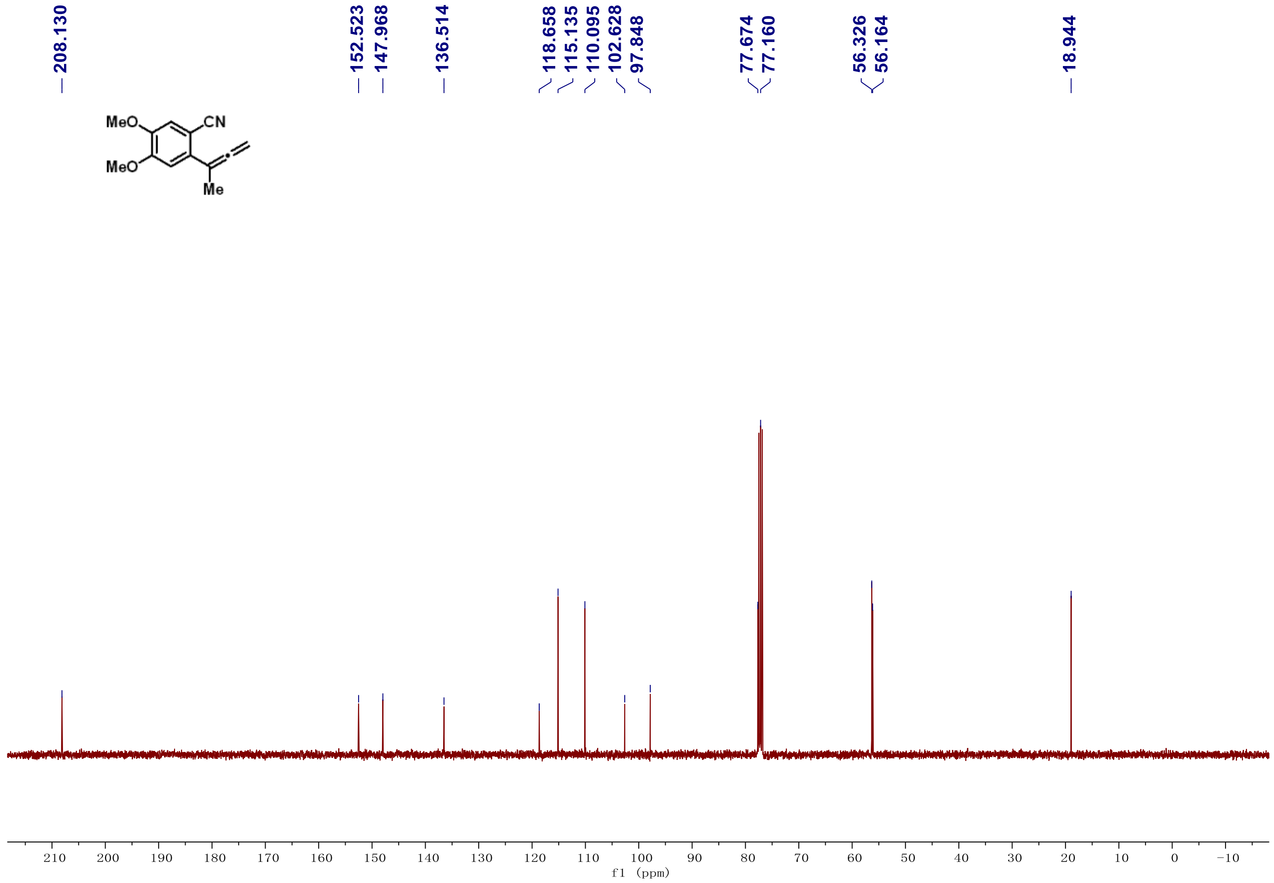

^{1}H NMR-spectrum (400 MHz, CDCl3) of **1h**

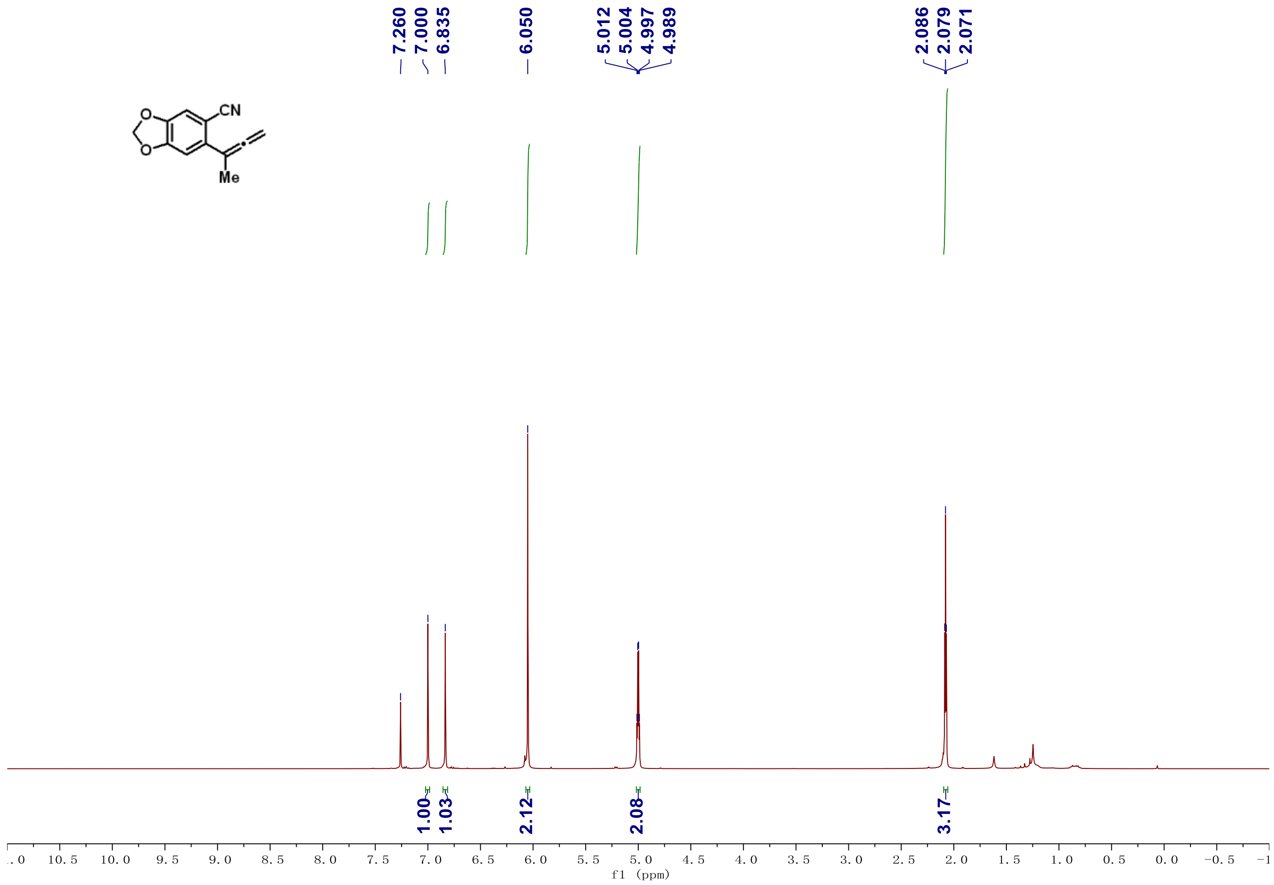

^{13}C NMR-spectrum (100 MHz, CDCl3) of **1h**

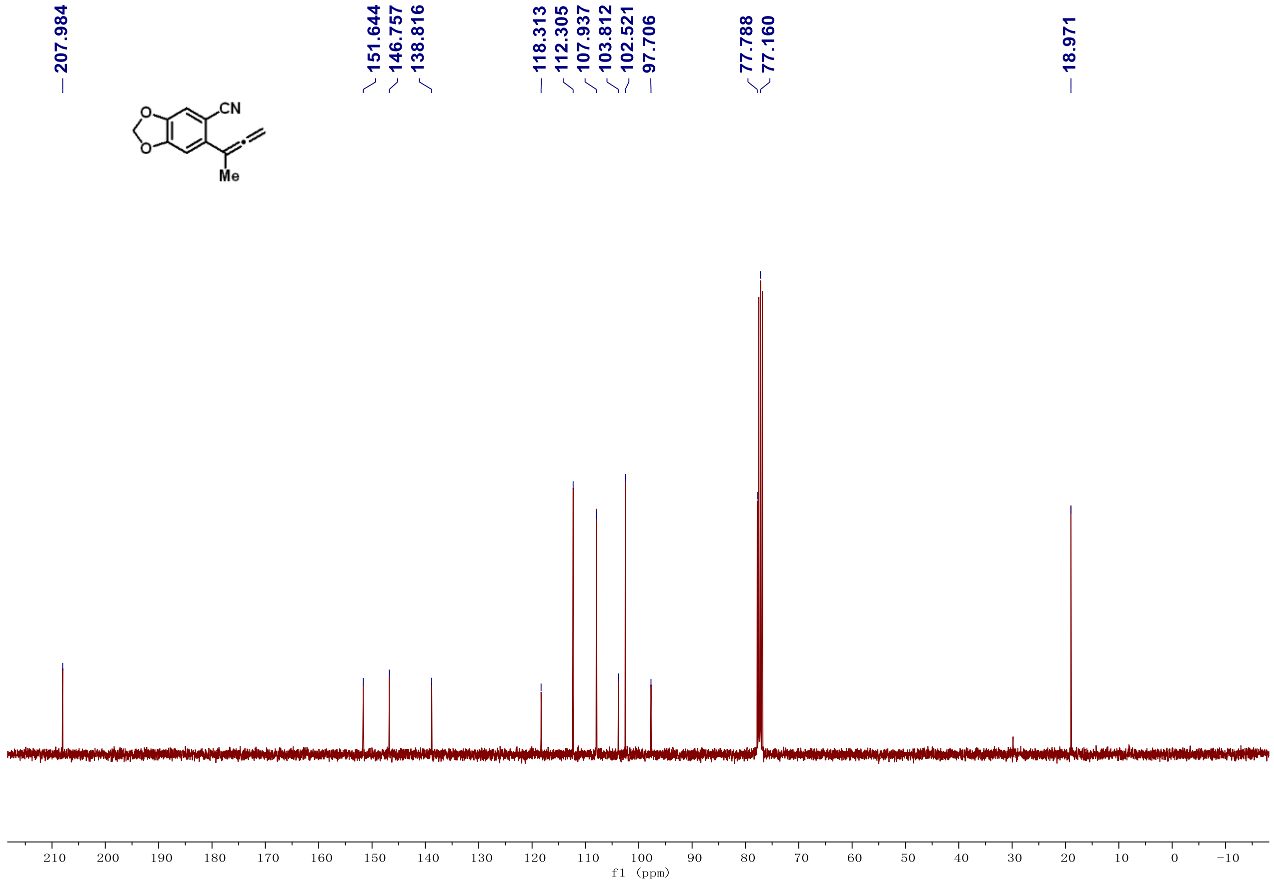

^{1}H NMR-spectrum (400 MHz, $CDCl_3$) of **1i**

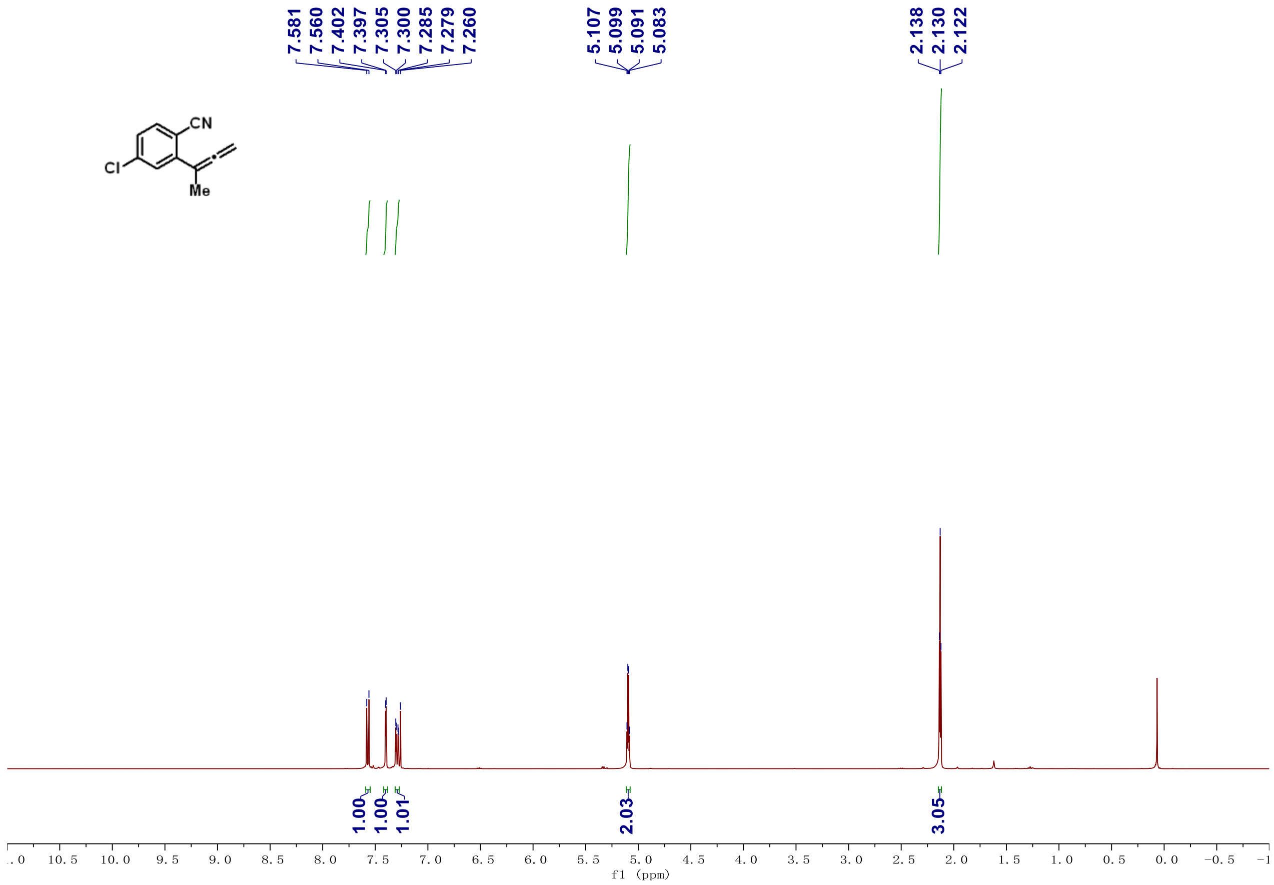

^{13}C NMR-spectrum (100 MHz, $CDCl_3$) of **1i**

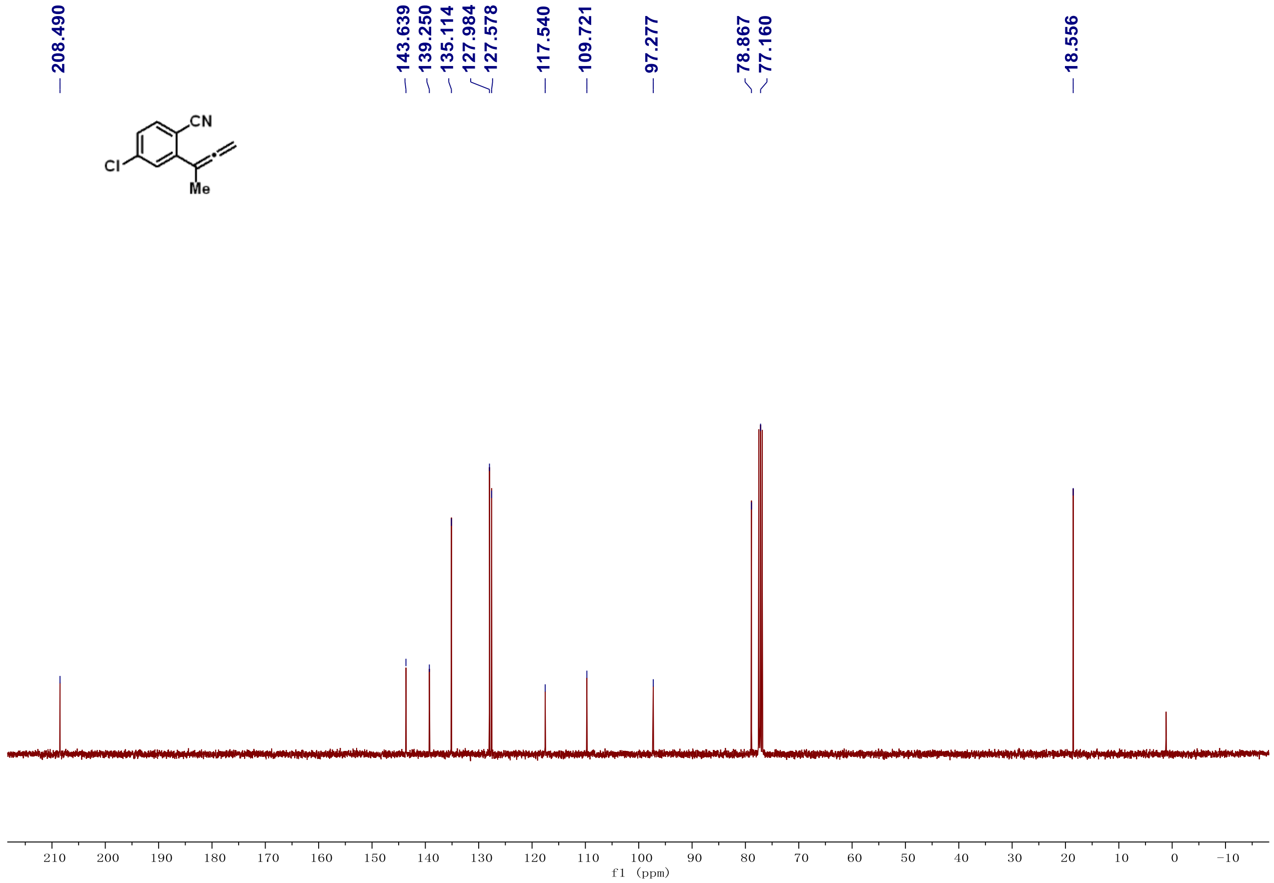

^{1}H NMR-spectrum (400 MHz, $CDCl_3$) of **1j**

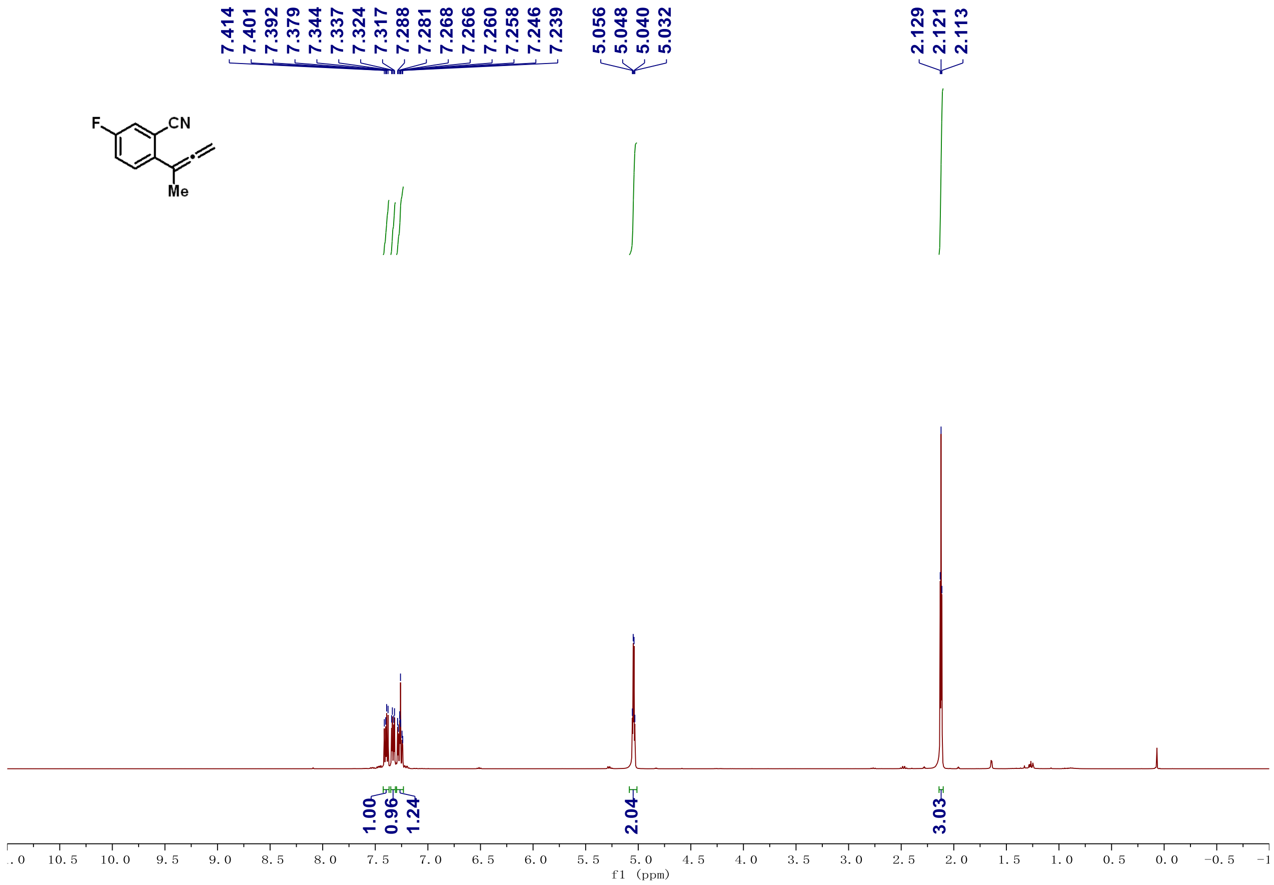

^{13}C NMR-spectrum (100 MHz, $CDCl_3$) of **1j**

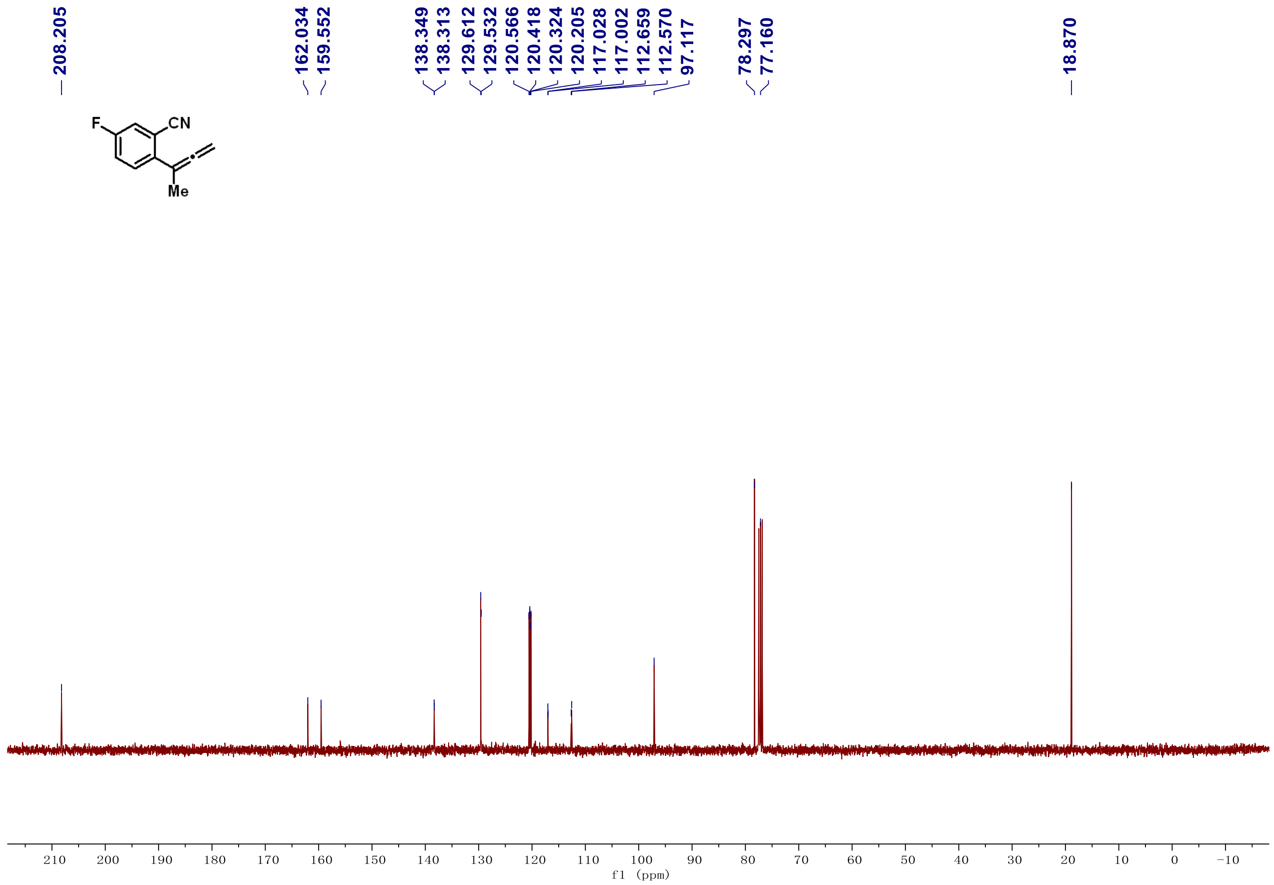

^{19}F NMR-spectrum (376 MHz, $CDCl_3$) of **1j**

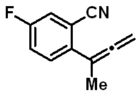

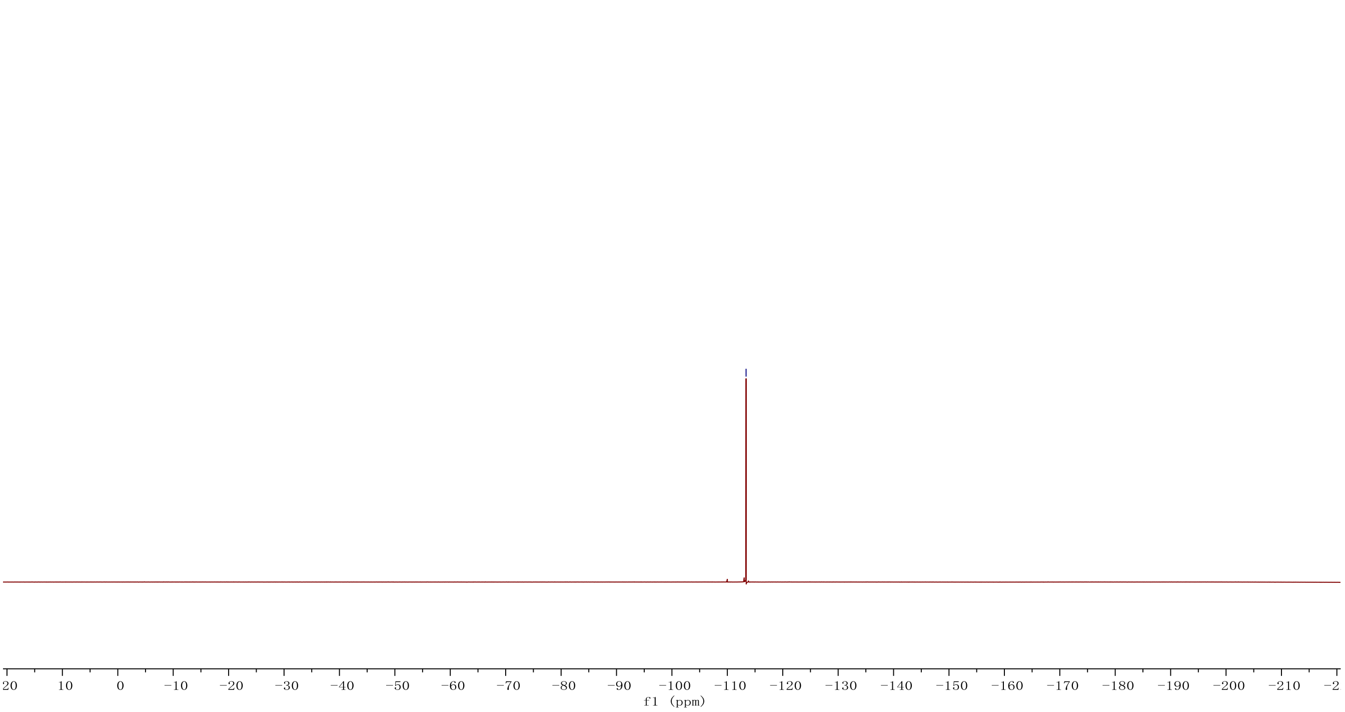

^{1}H NMR-spectrum (400 MHz, $CDCl_3$) of **1k**

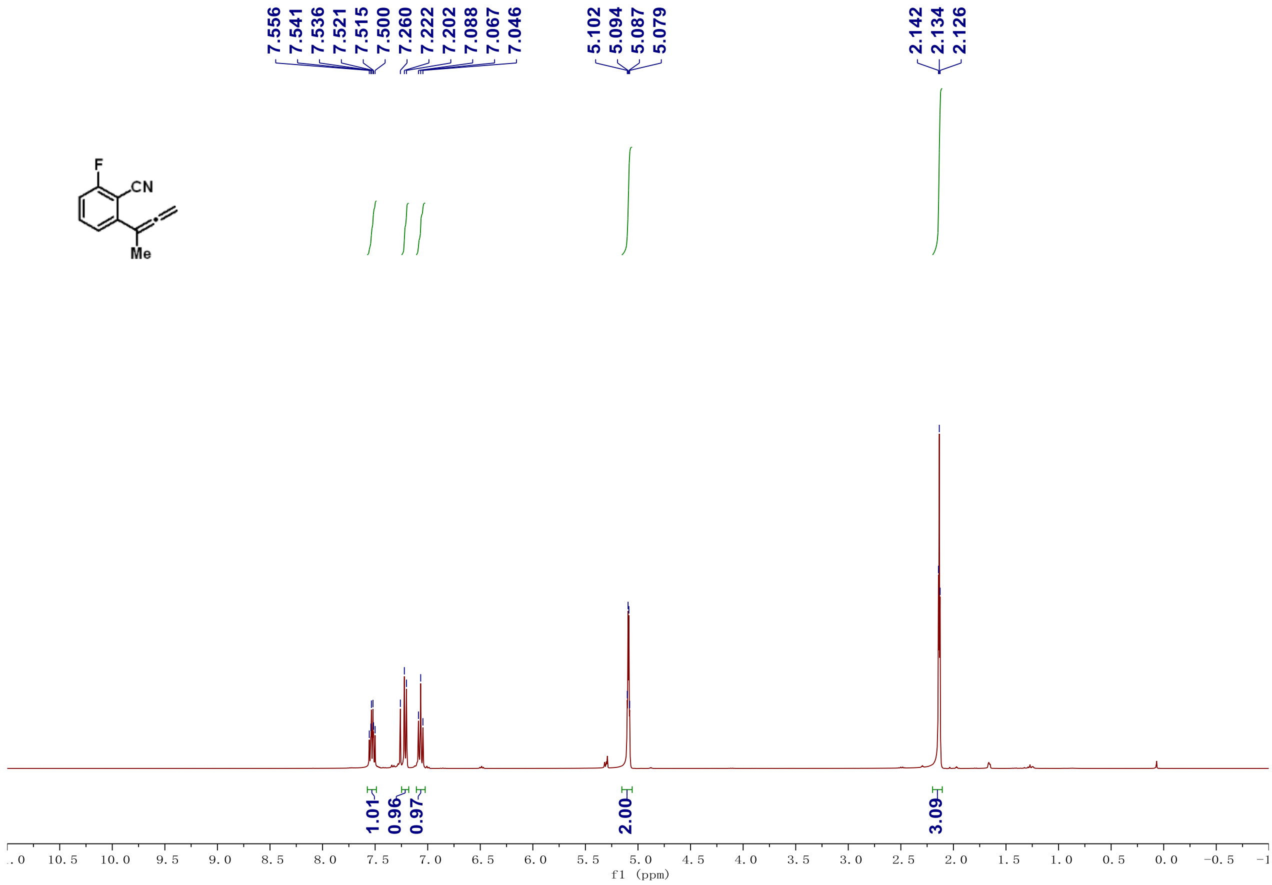

^{13}C NMR-spectrum (100 MHz, $CDCl_3$) of **1k**

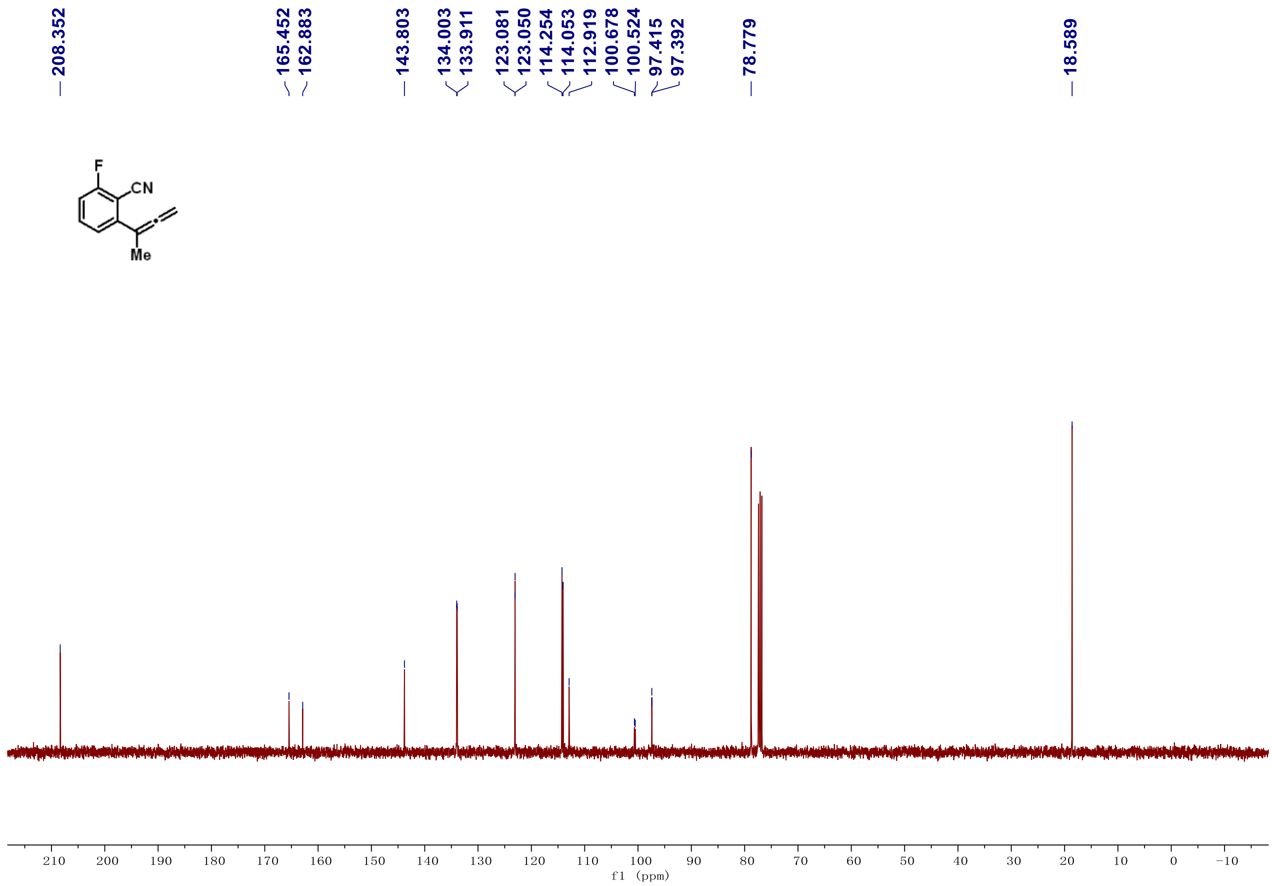

^{19}F NMR-spectrum (376 MHz, $CDCl_3$) of **1k**

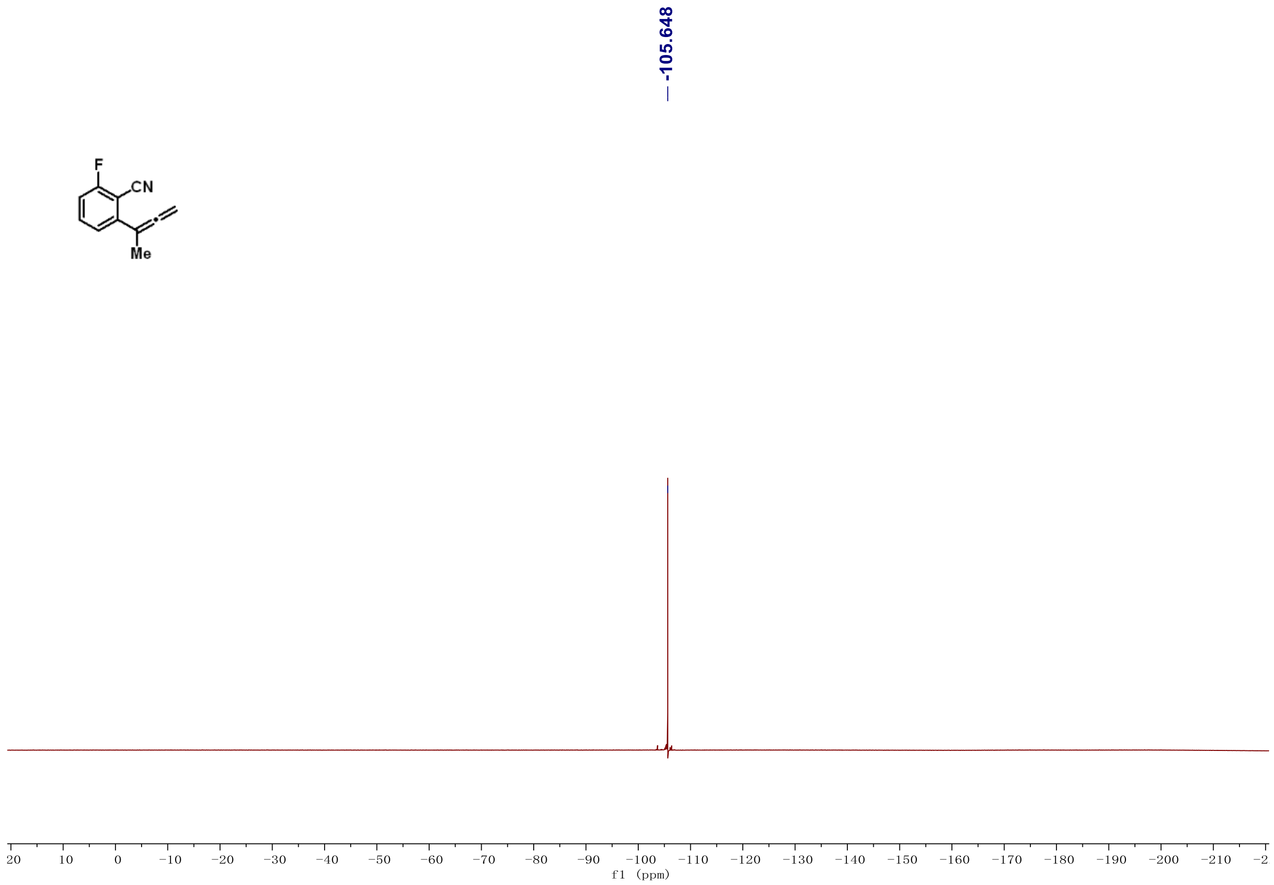

^{1}H NMR-spectrum (400 MHz, $CDCl_3$) of **1l**

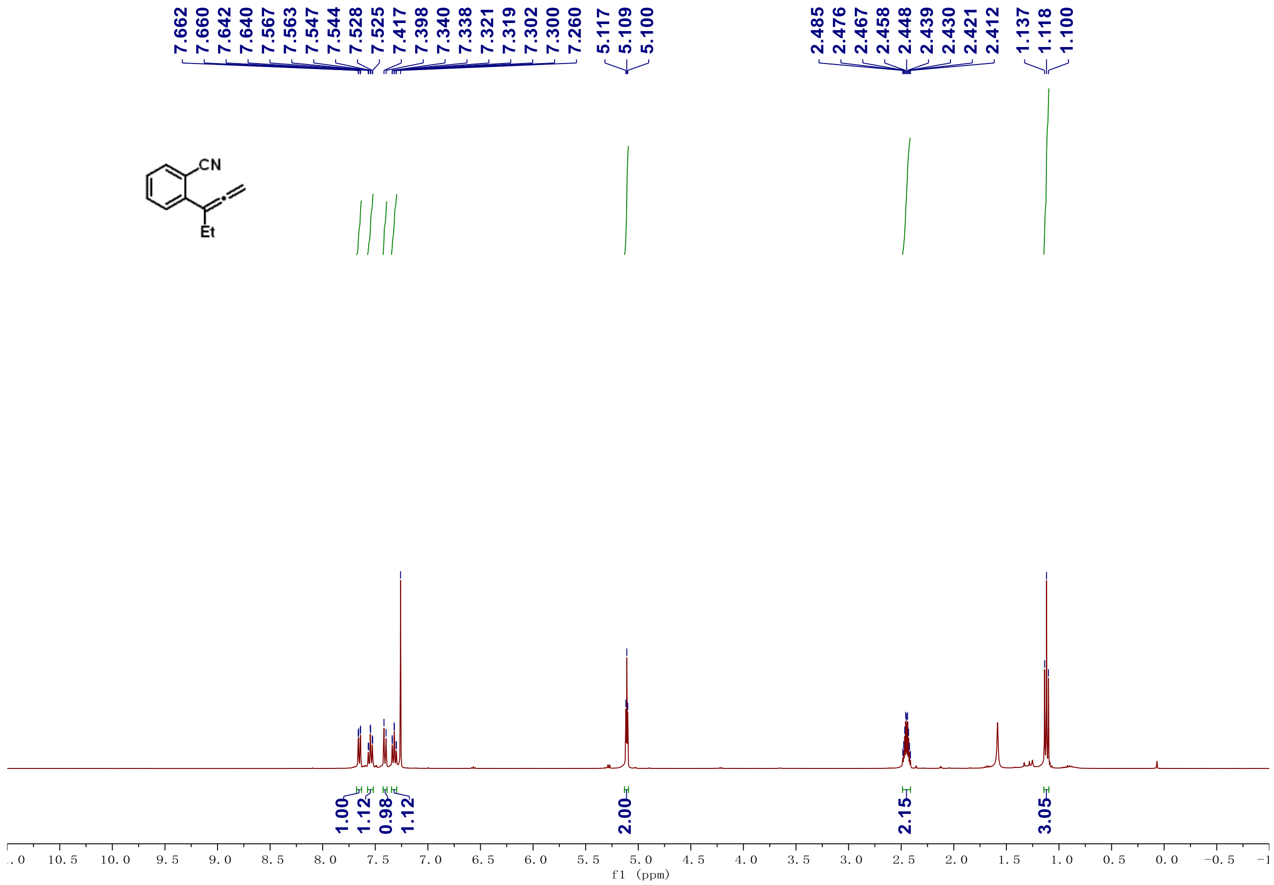

^{13}C NMR-spectrum (100 MHz, $CDCl_3$) of **1l**

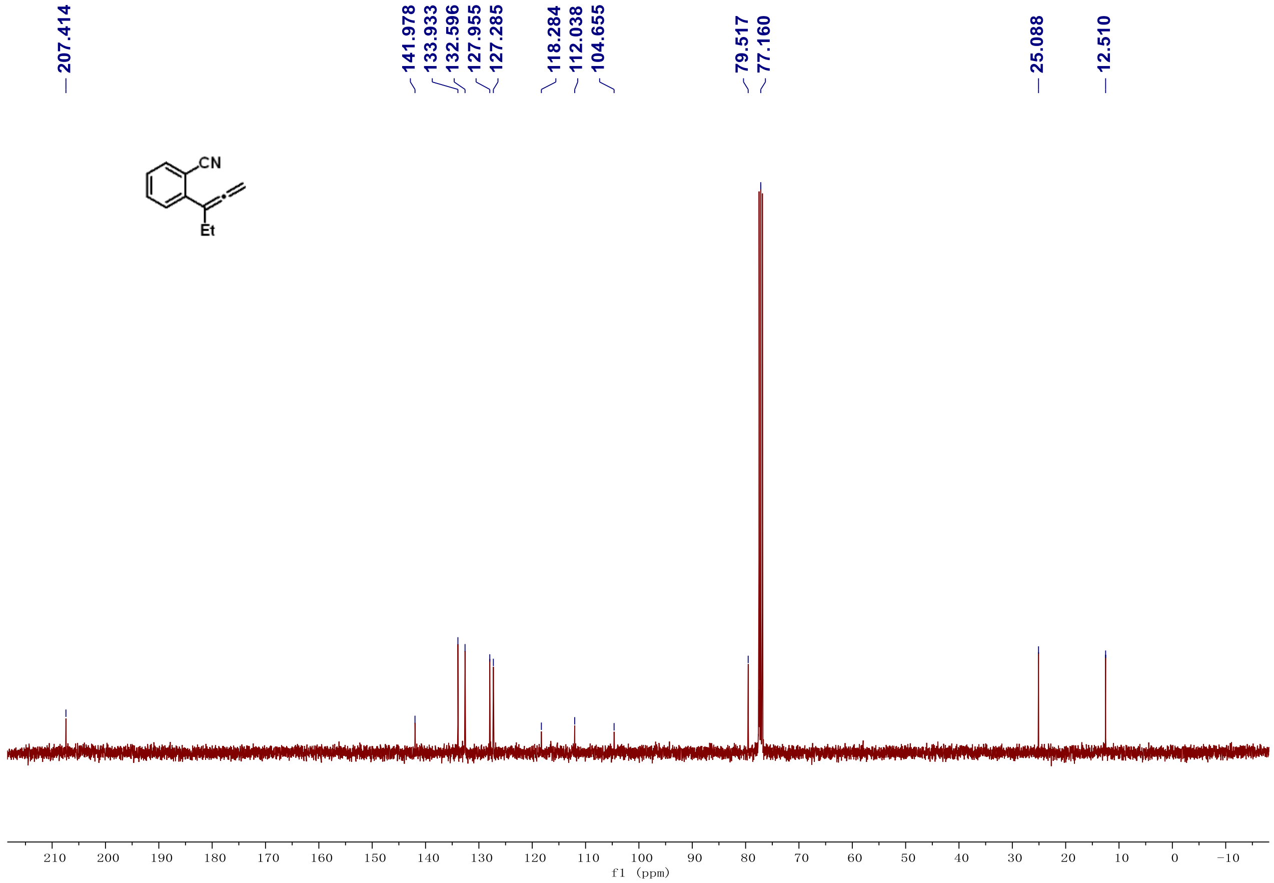

^{1}H NMR-spectrum (400 MHz, $CDCl_3$) of **1m**

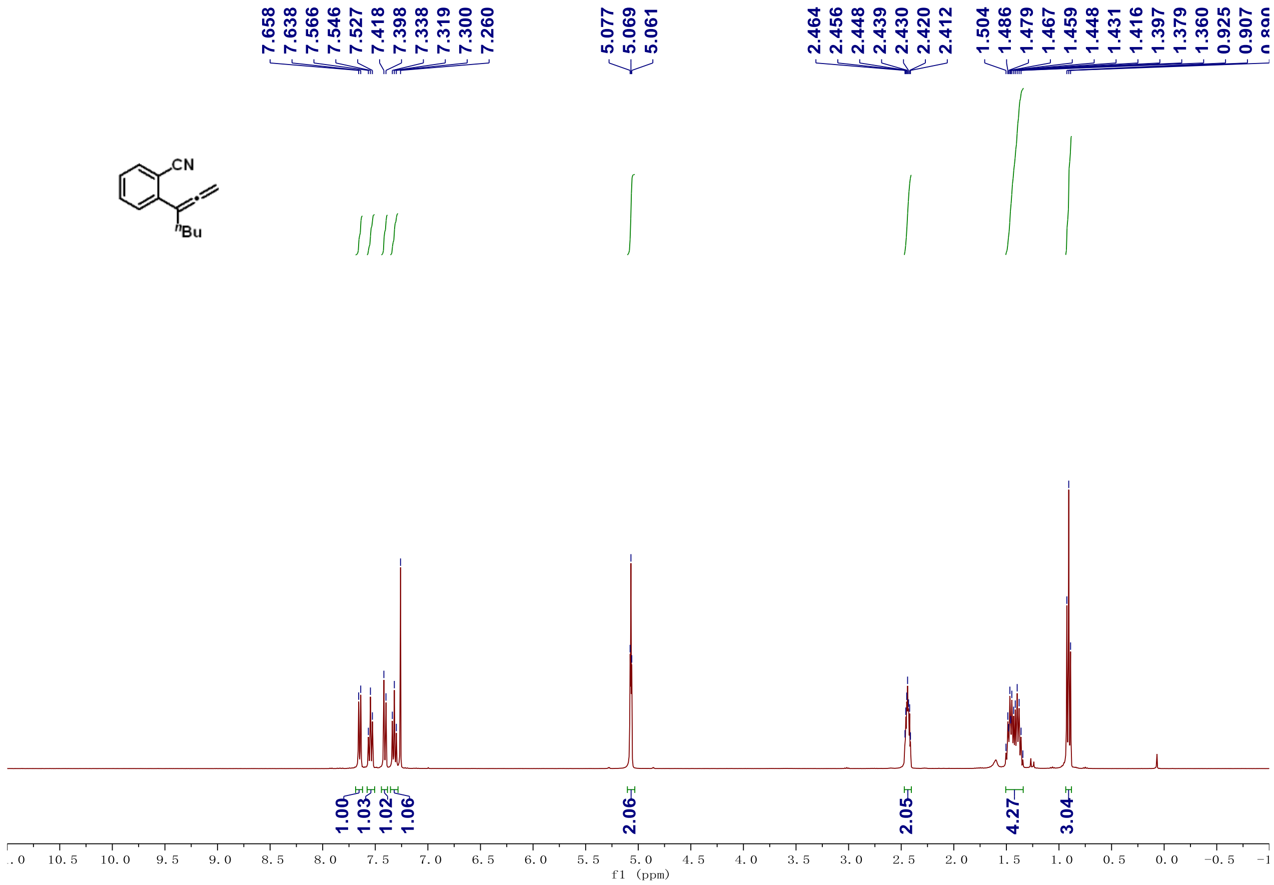

^{13}C NMR-spectrum (100 MHz, $CDCl_3$) of **1m**

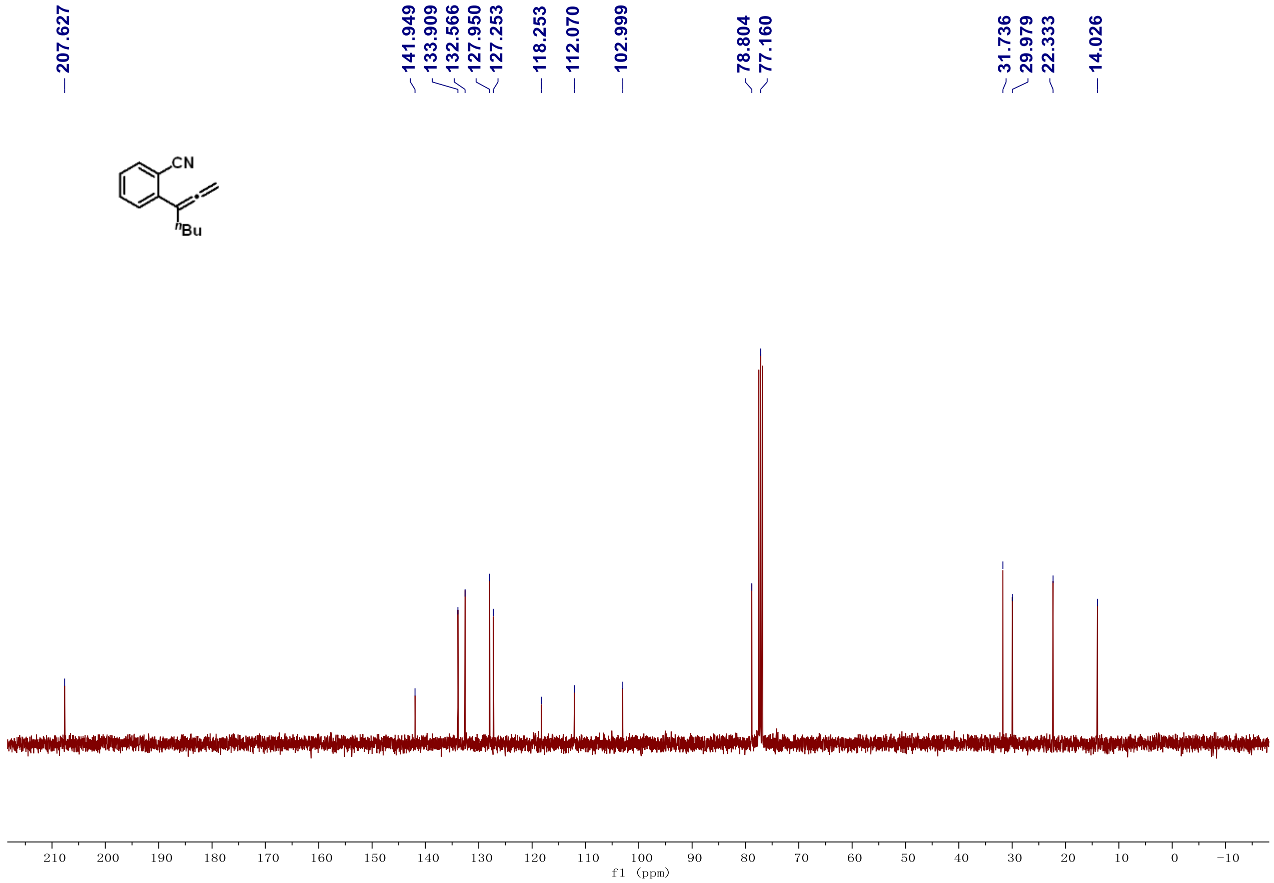

^{1}H NMR-spectrum (400 MHz, $CDCl_3$) of **3a**

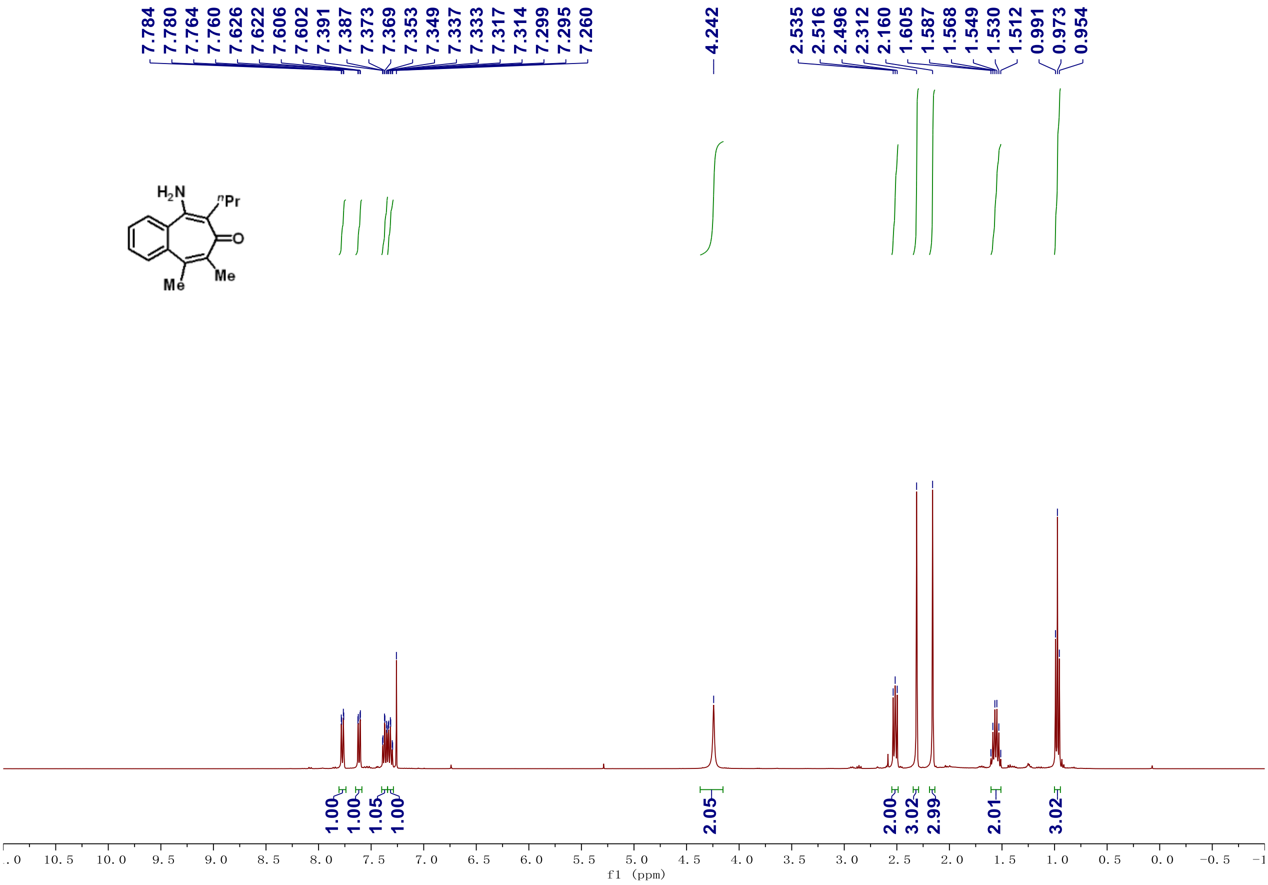

^{13}C NMR-spectrum (100 MHz, $CDCl_3$) of **3a**

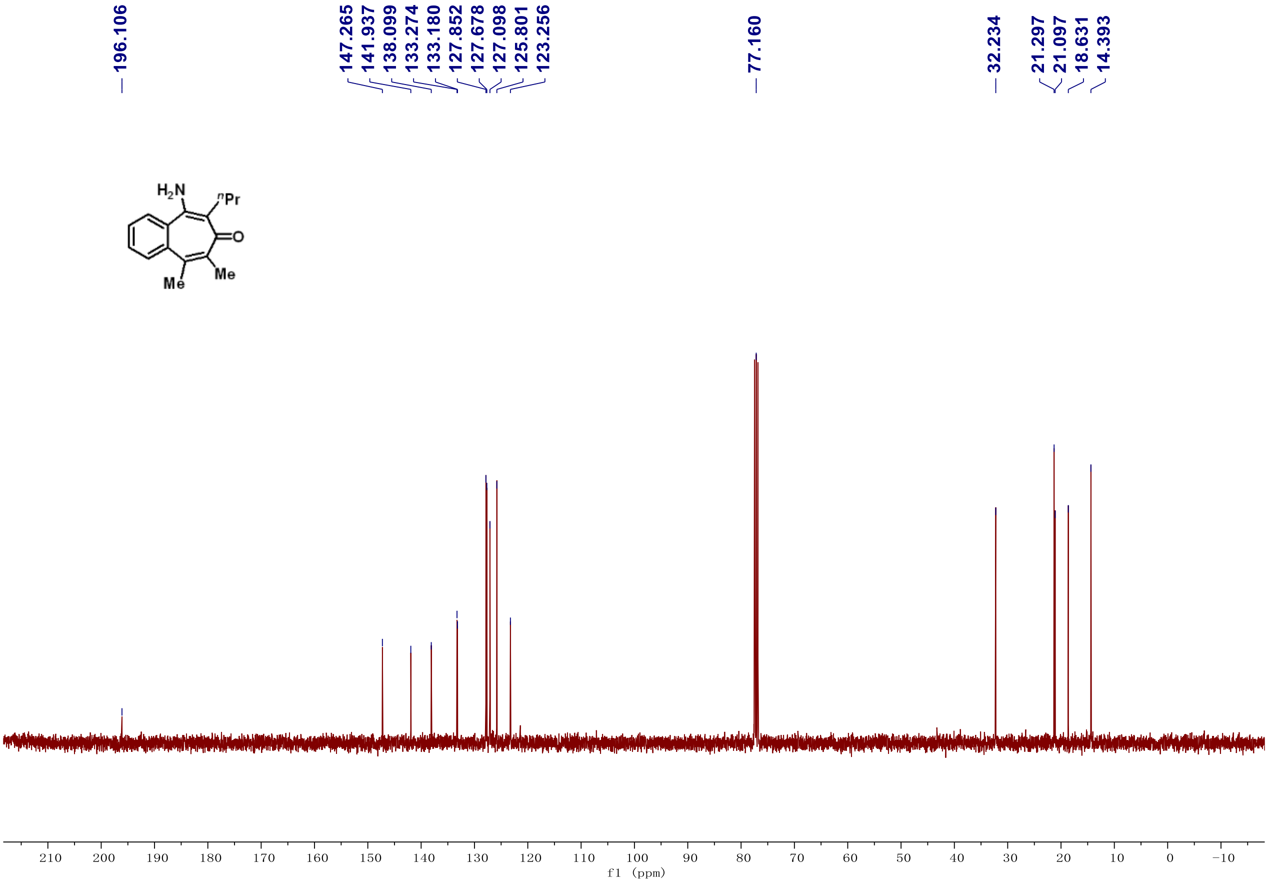

^{1}H NMR-spectrum (400 MHz, $CDCl_3$) of **3b**

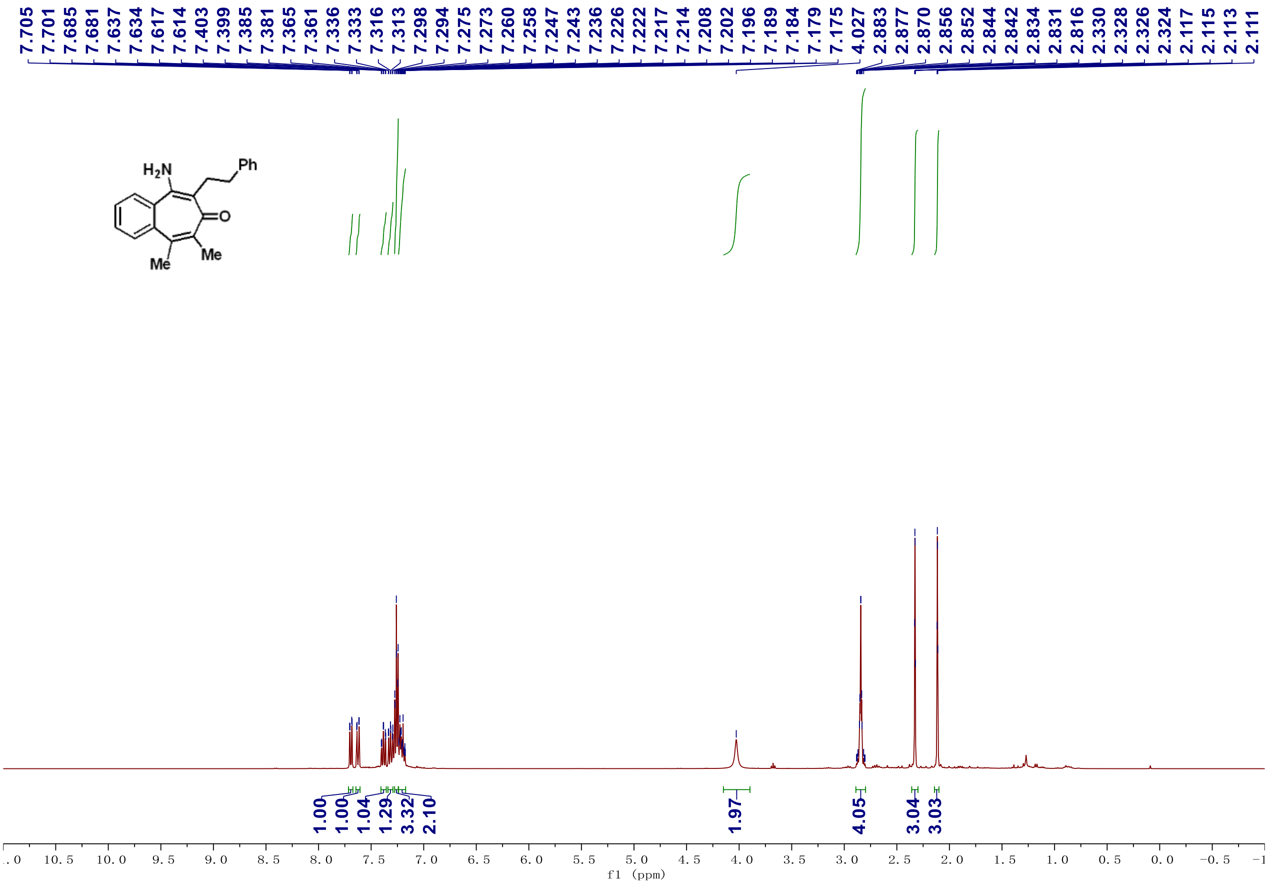

^{13}C NMR-spectrum (100 MHz, $CDCl_3$) of **3b**

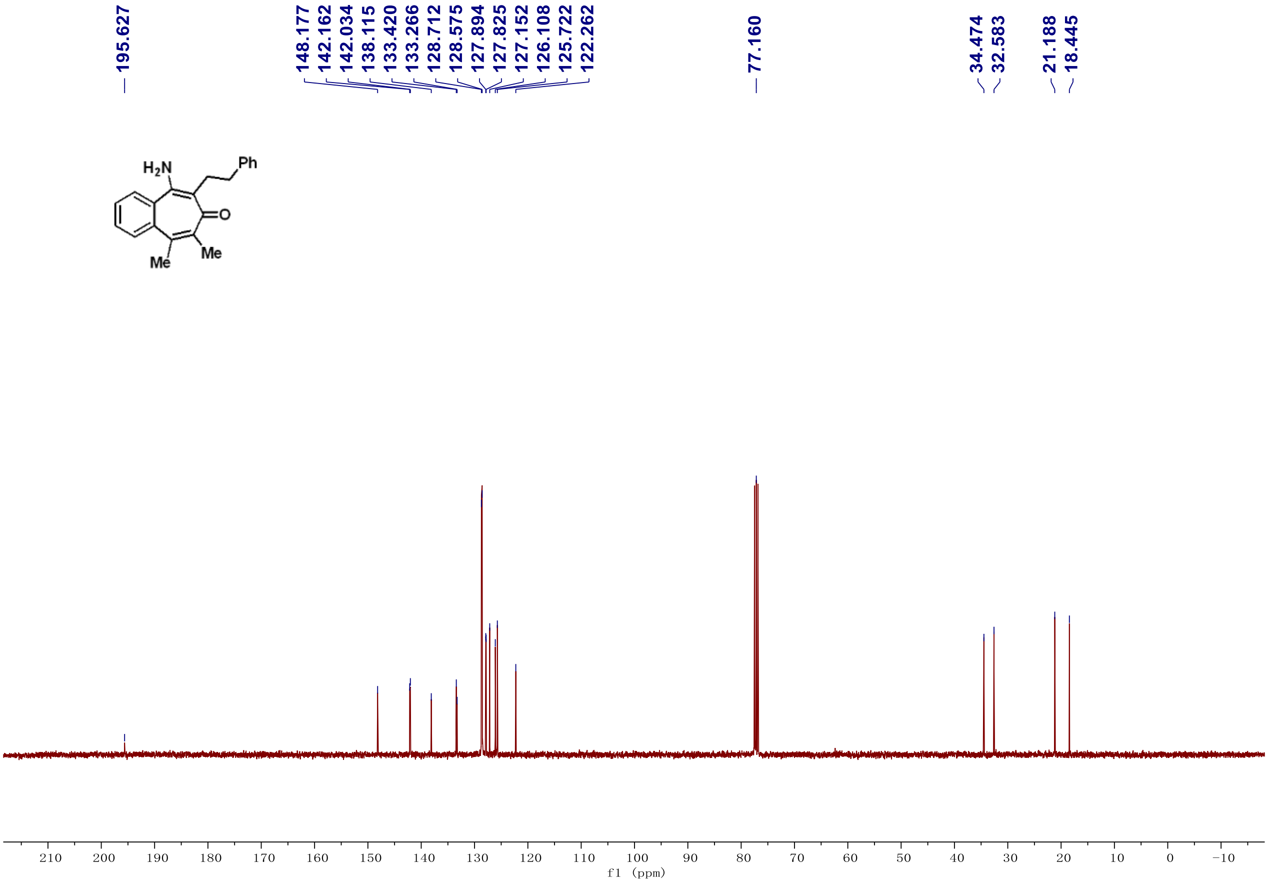

^{1}H NMR-spectrum (400 MHz, $CDCl_3$) of **3c**

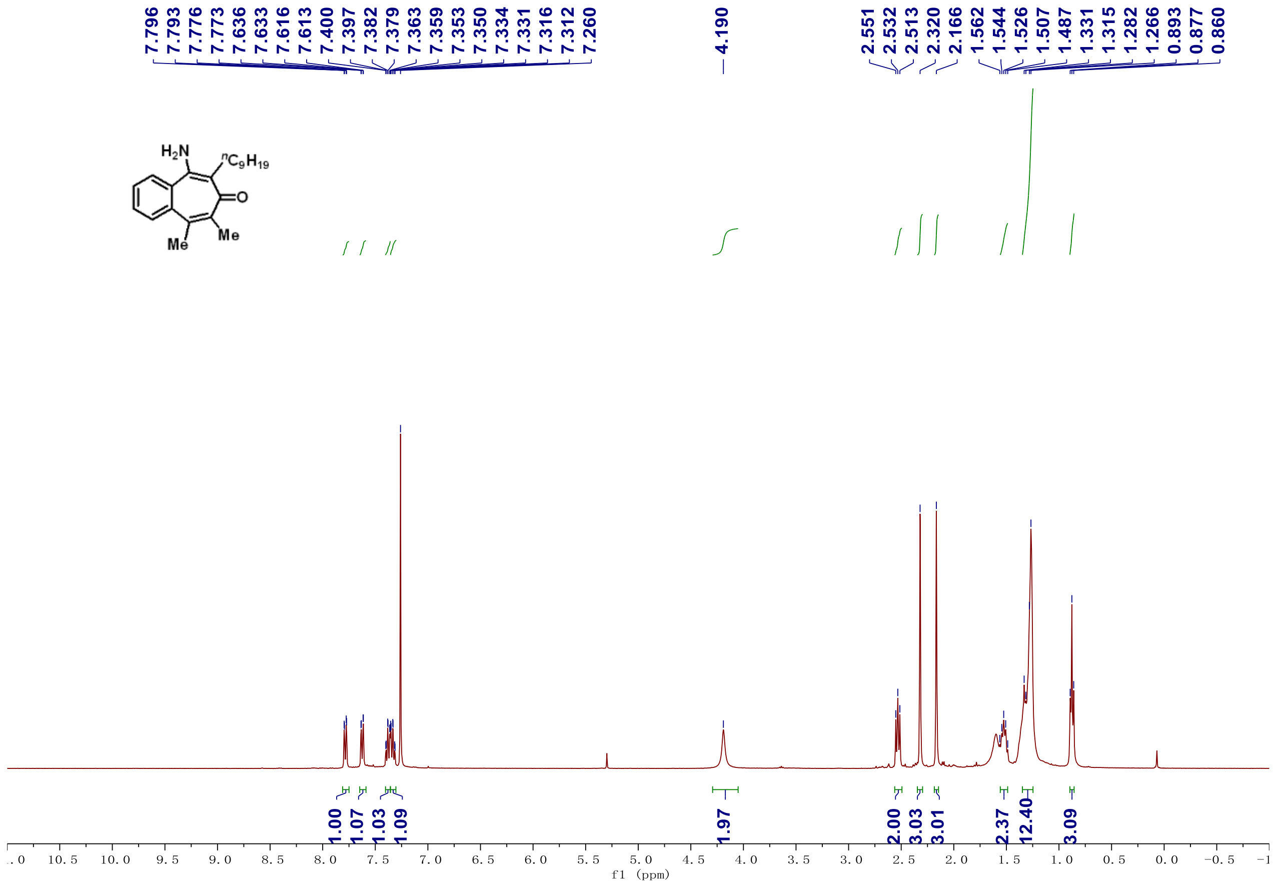

^{13}C NMR-spectrum (100 MHz, $CDCl_3$) of **3c**

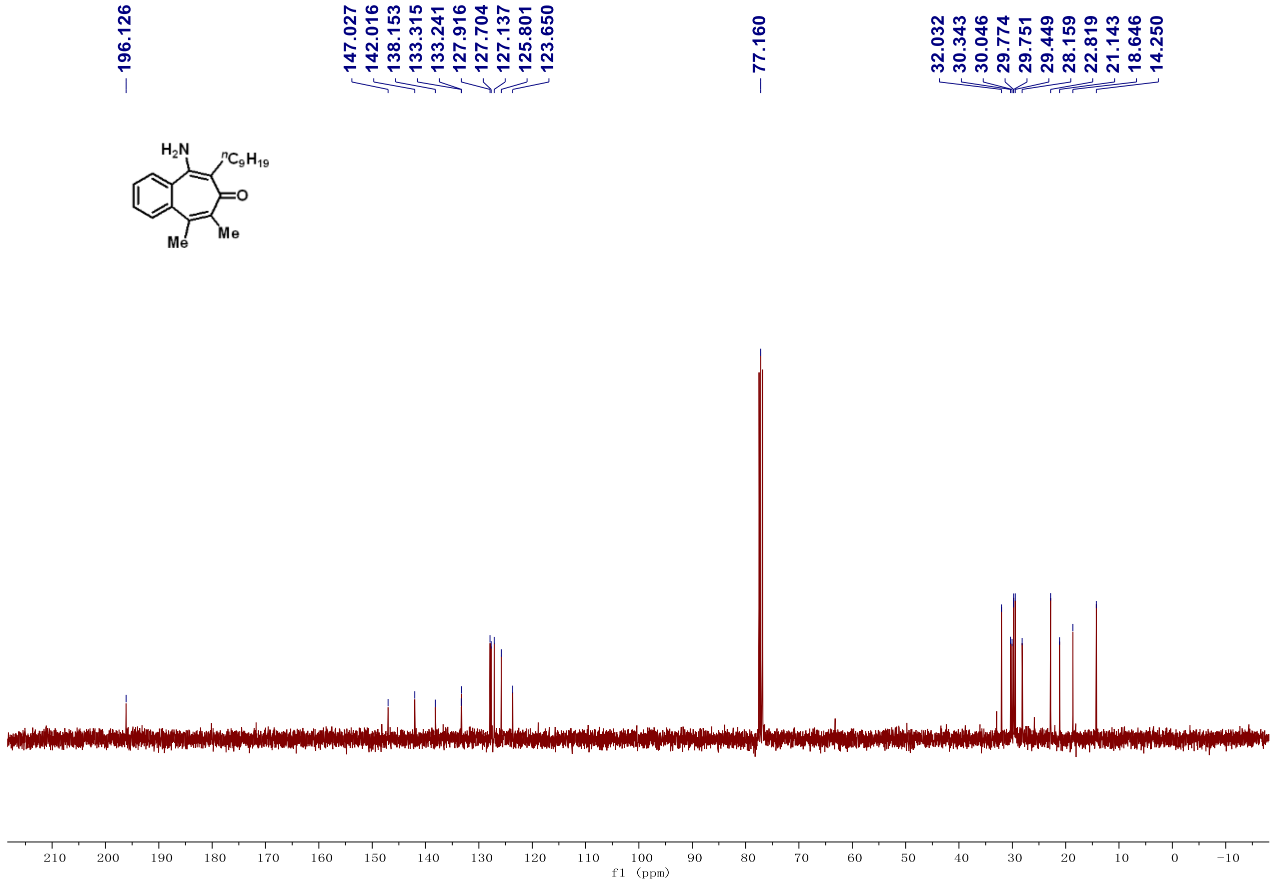

^{1}H NMR-spectrum (400 MHz, $CDCl_3$) of **3d**

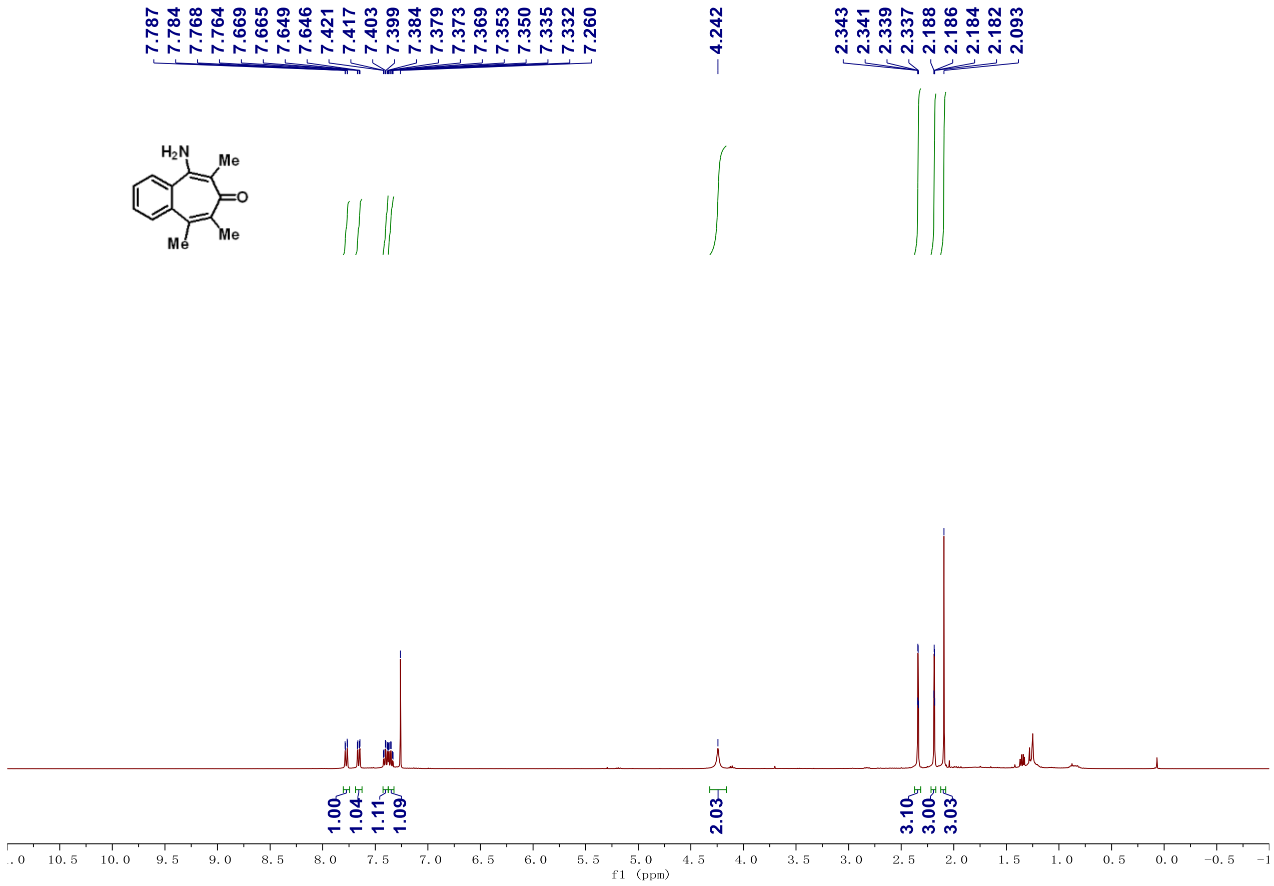

^{13}C NMR-spectrum (100 MHz, $CDCl_3$) of **3d**

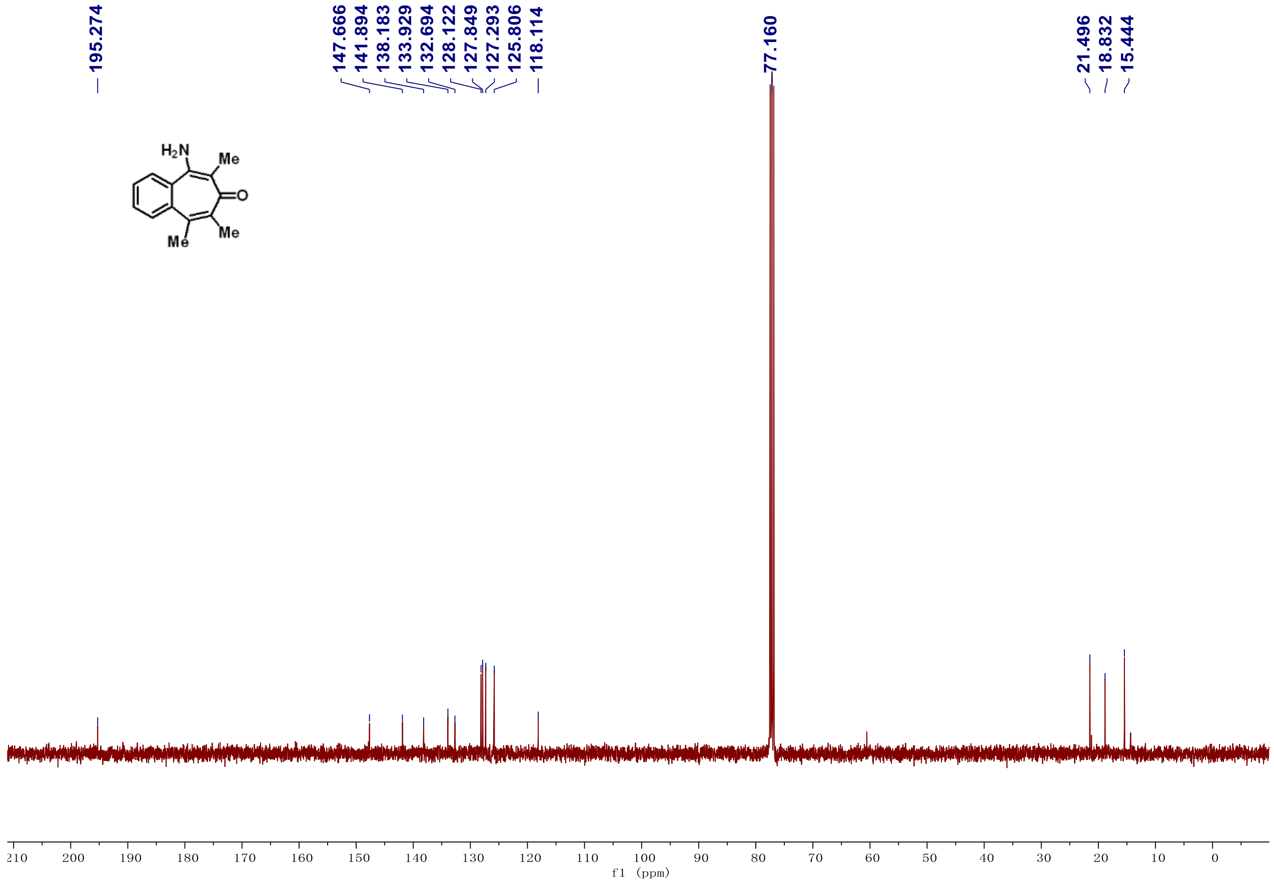

^{1}H NMR-spectrum (400 MHz, $CDCl_3$) of **3e**

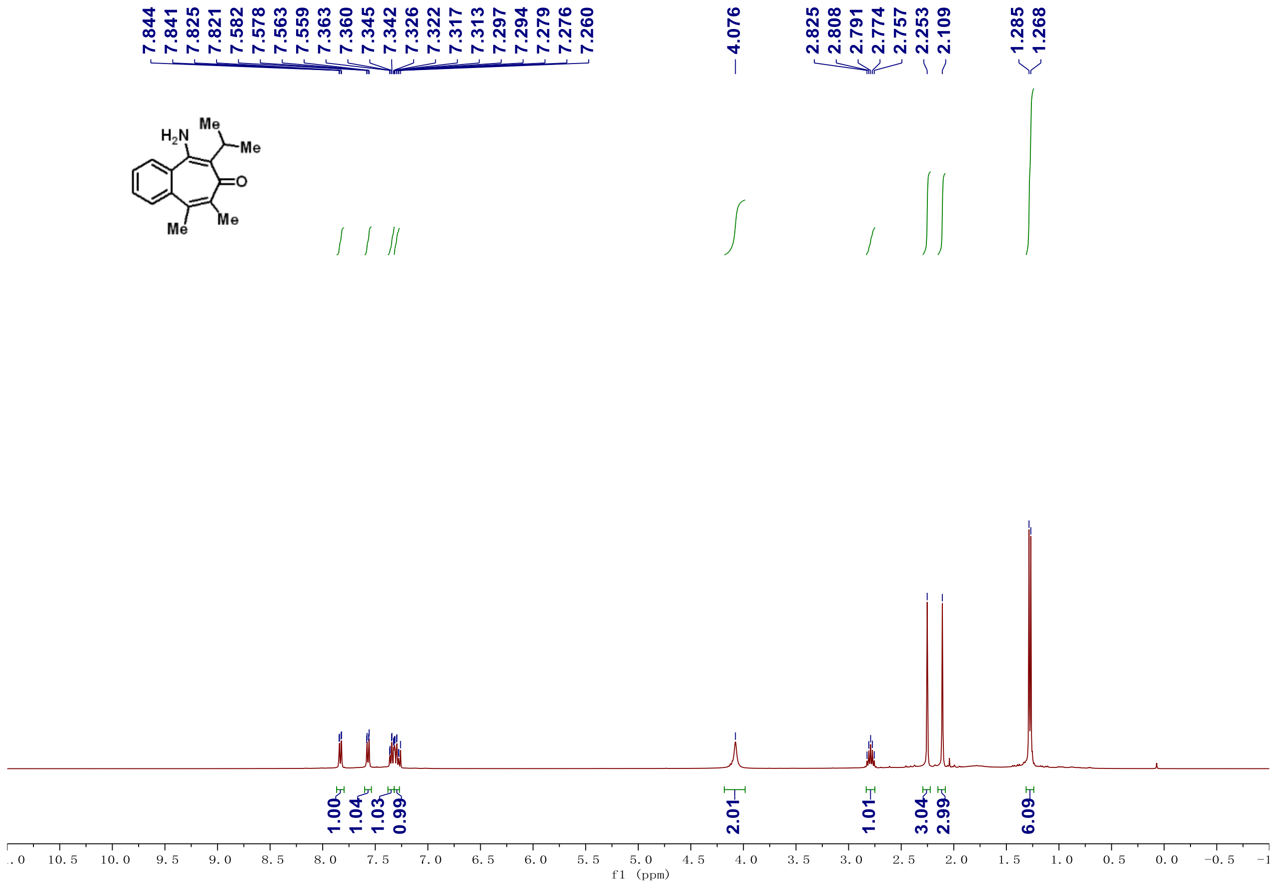

^{13}C NMR-spectrum (100 MHz, $CDCl_3$) of **3e**

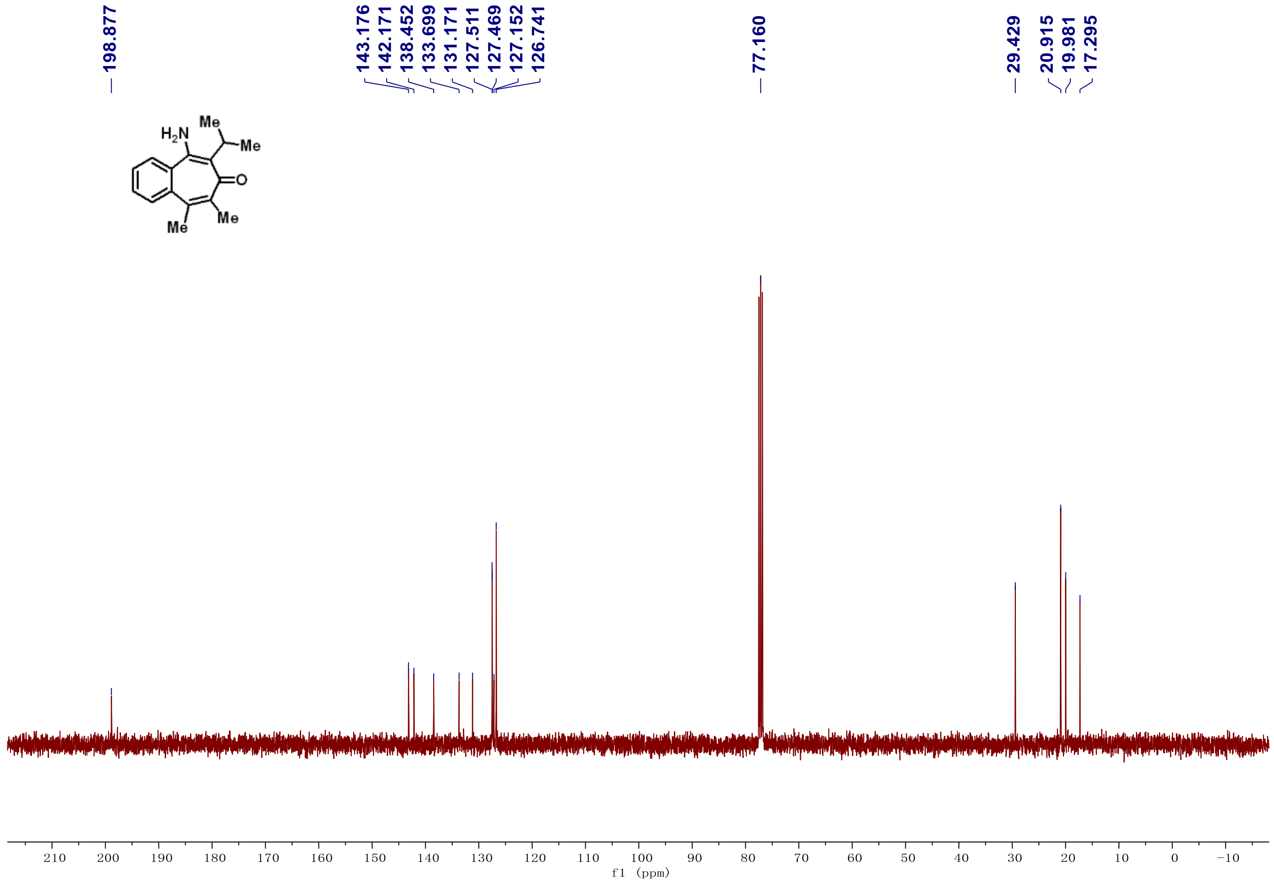

^{1}H NMR-spectrum (400 MHz, $CDCl_3$) of **3f**

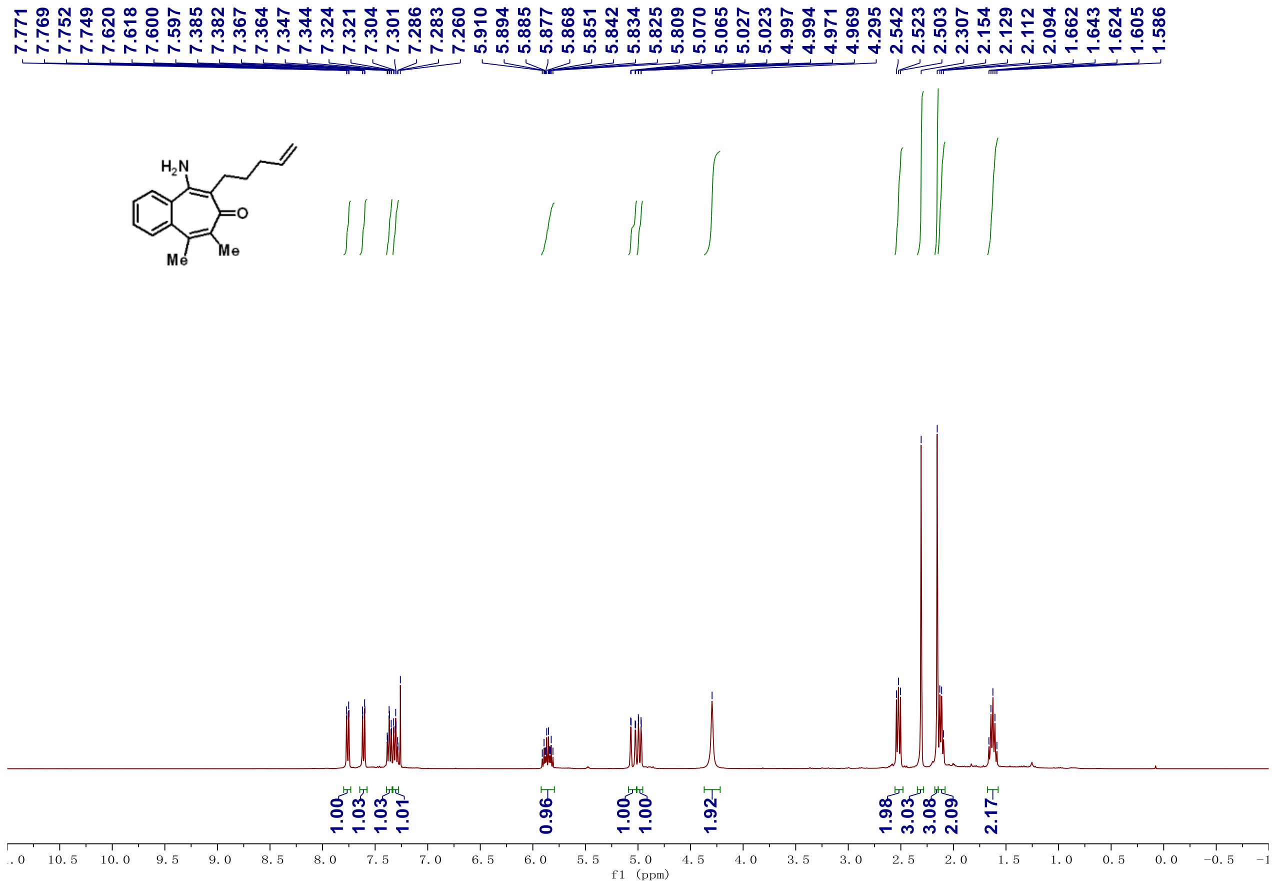

^{13}C NMR-spectrum (100 MHz, $CDCl_3$) of **3f**

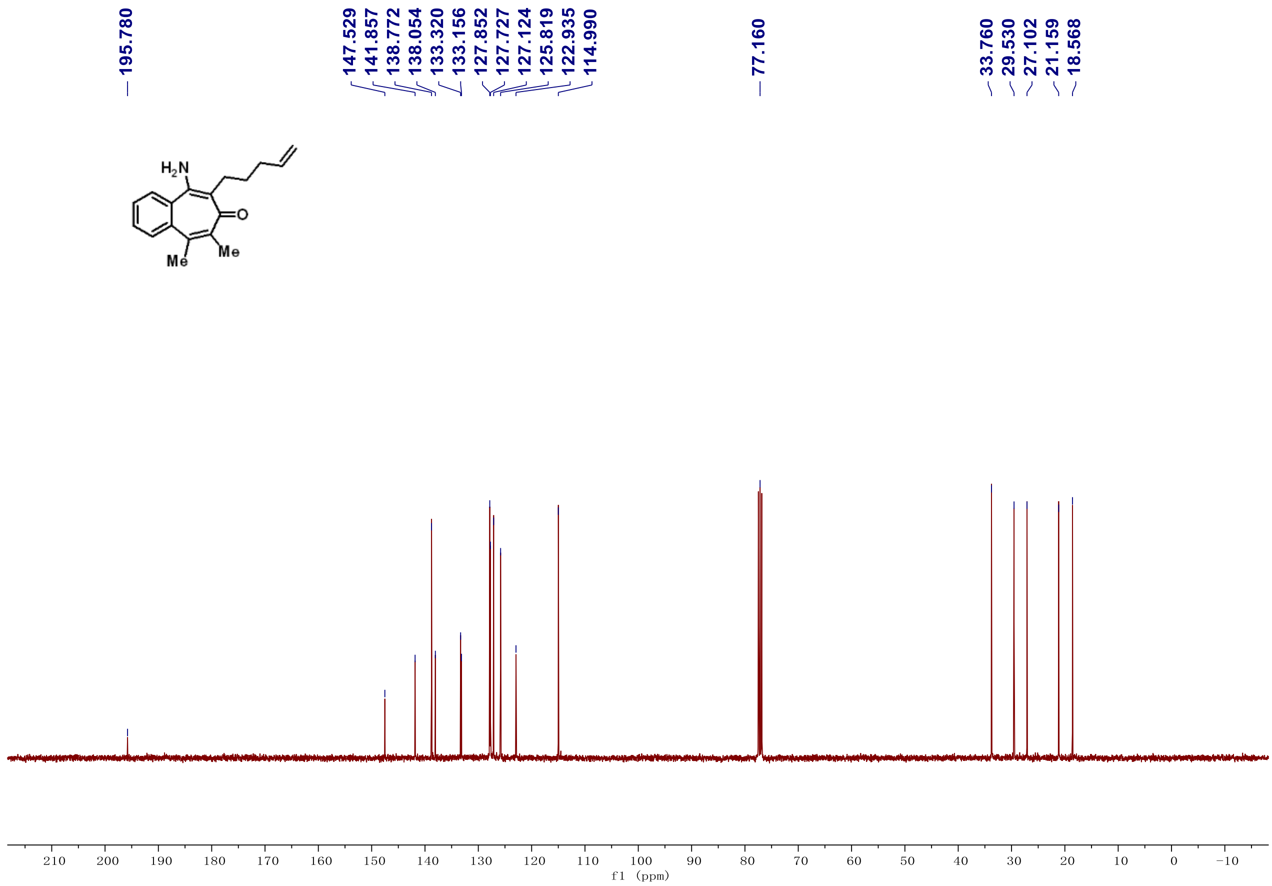

[1]**H NMR**-spectrum (400 MHz, $CDCl_3$) of **3g**

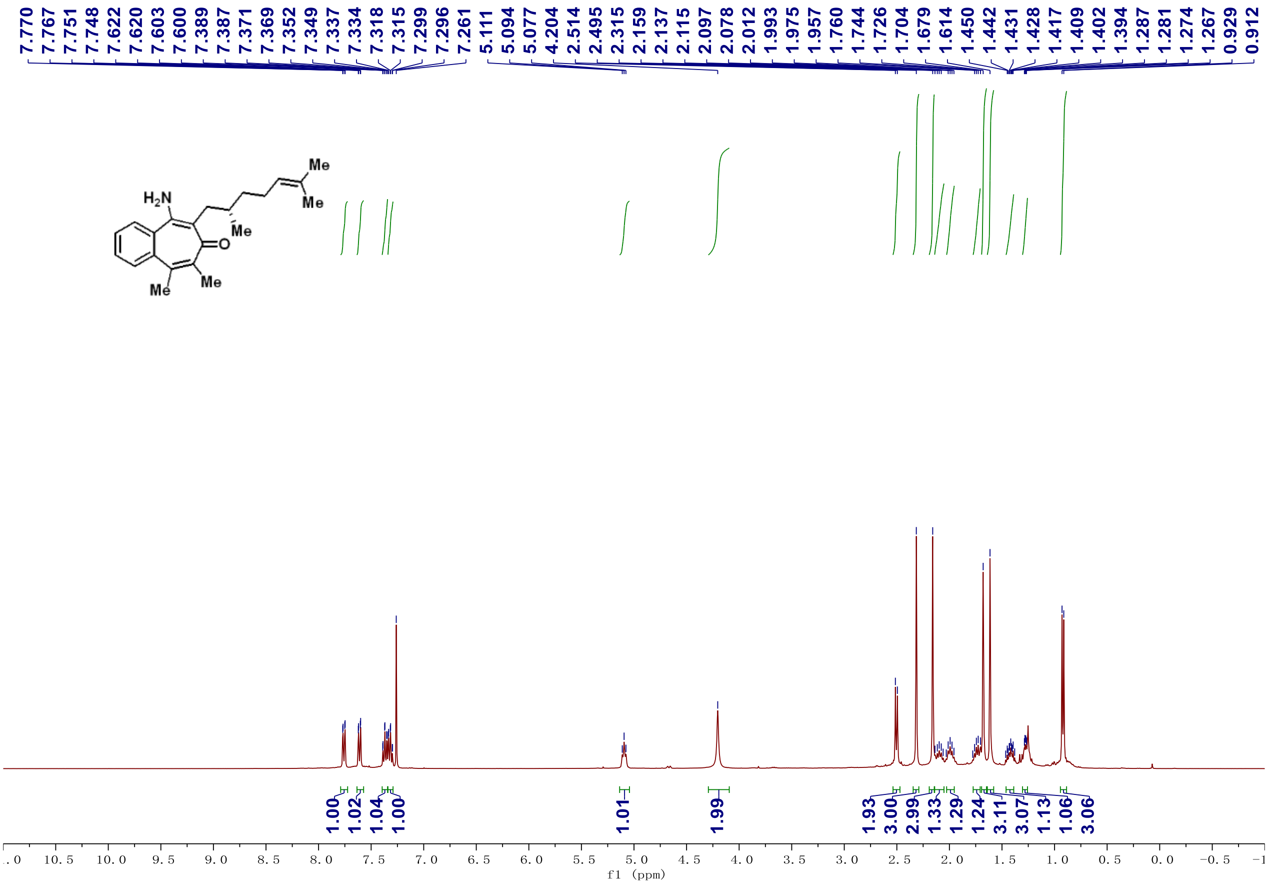

[13]**C NMR**-spectrum (100 MHz, $CDCl_3$) of **3g**

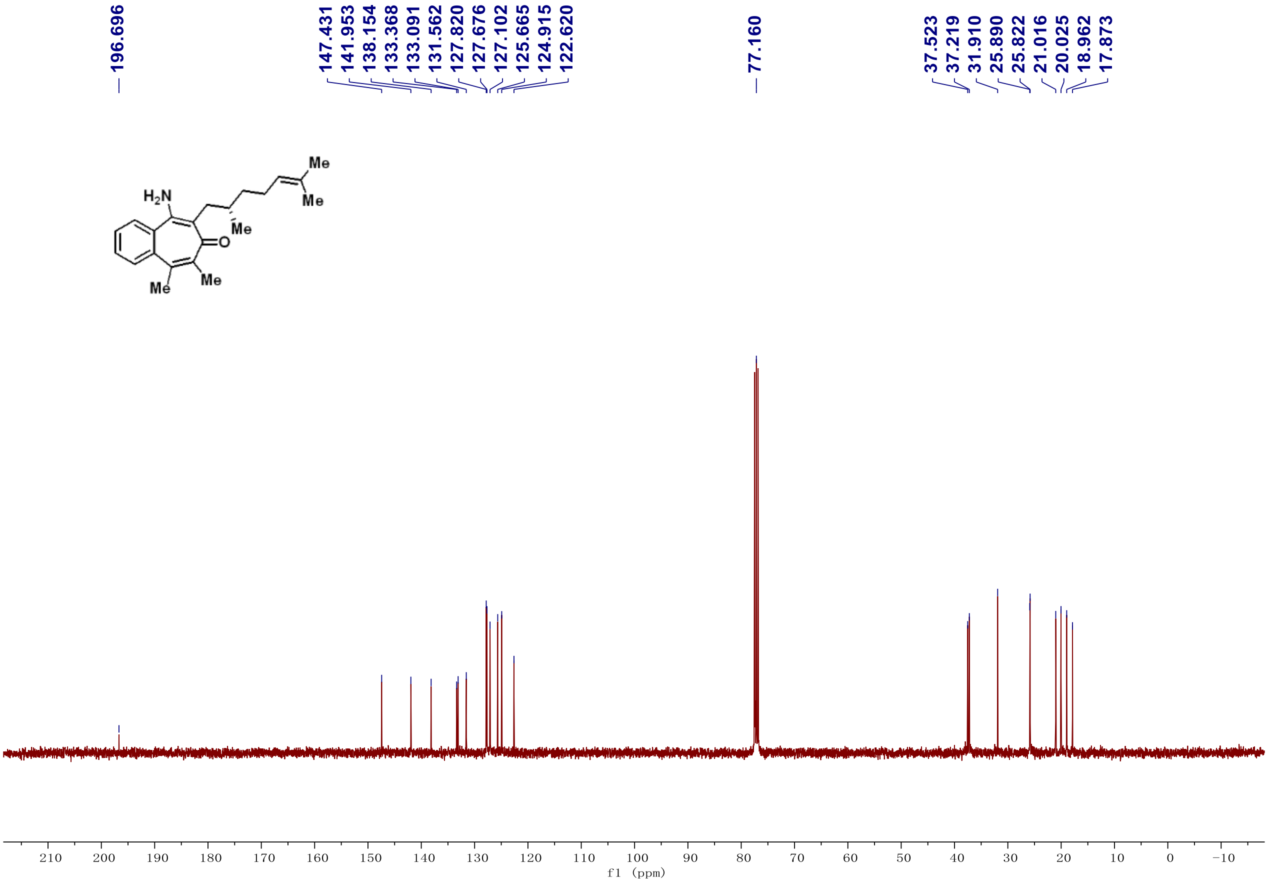

^{1}H NMR-spectrum (400 MHz, $CDCl_3$) of **3h**

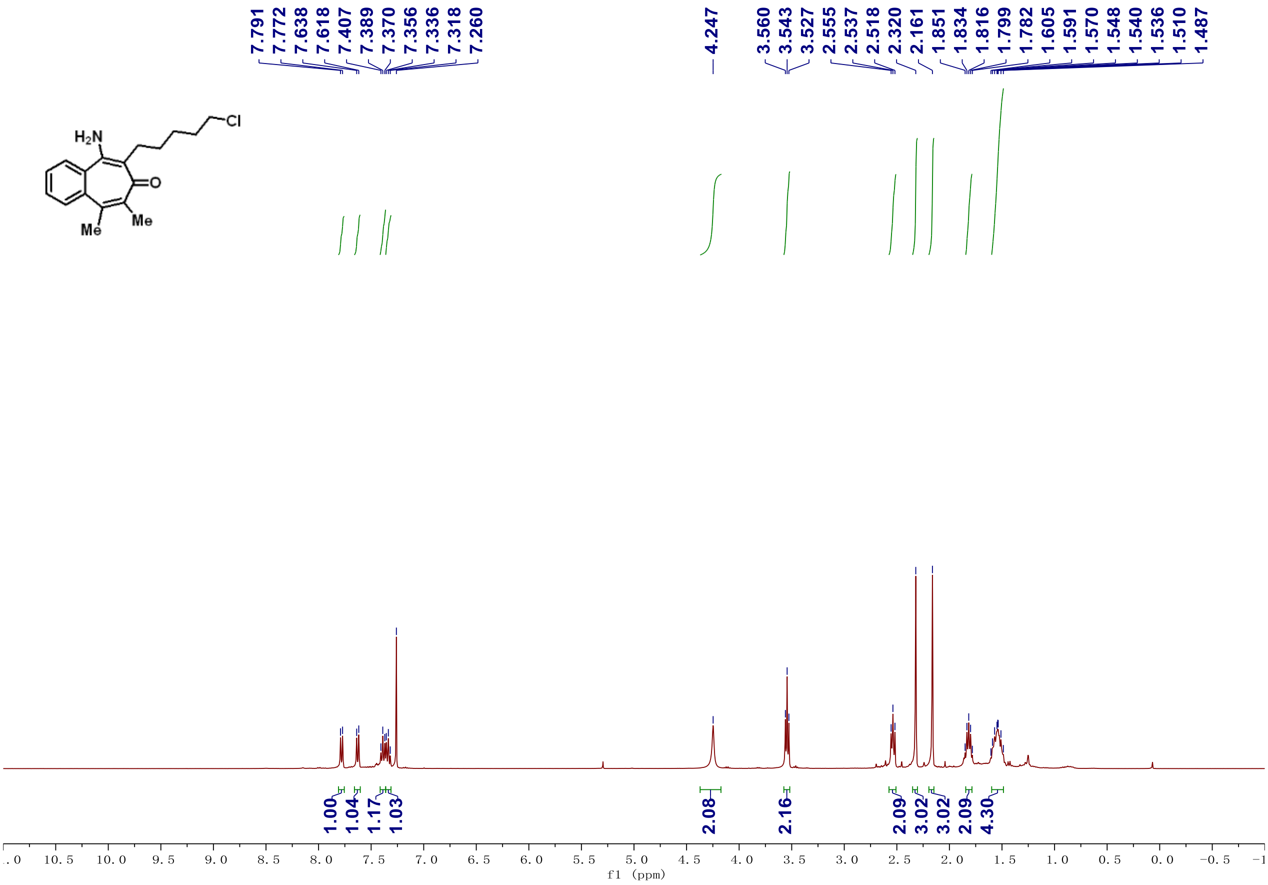

^{13}C NMR-spectrum (100 MHz, $CDCl_3$) of **3h**

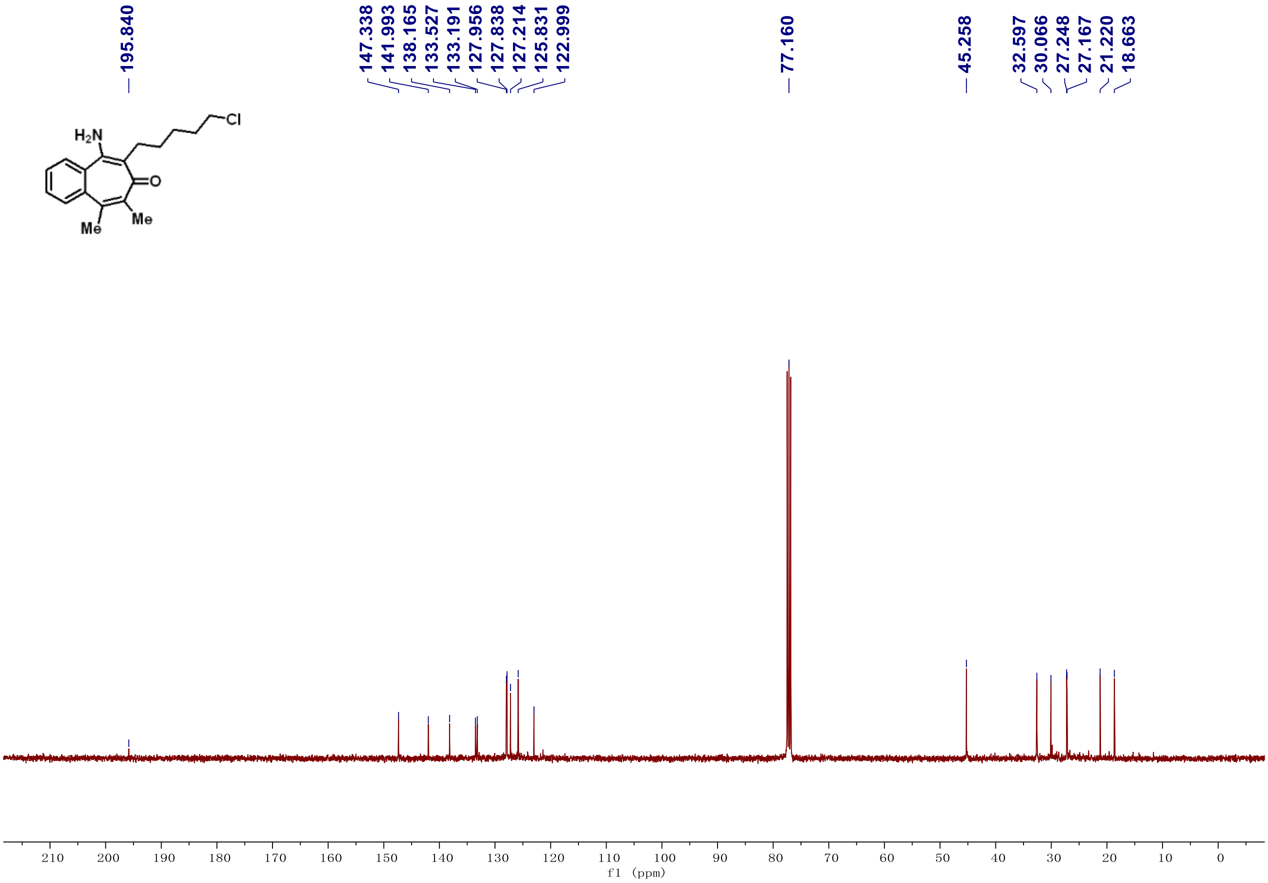

^{1}H **NMR**-spectrum (400 MHz, $CDCl_3$) of **3i**

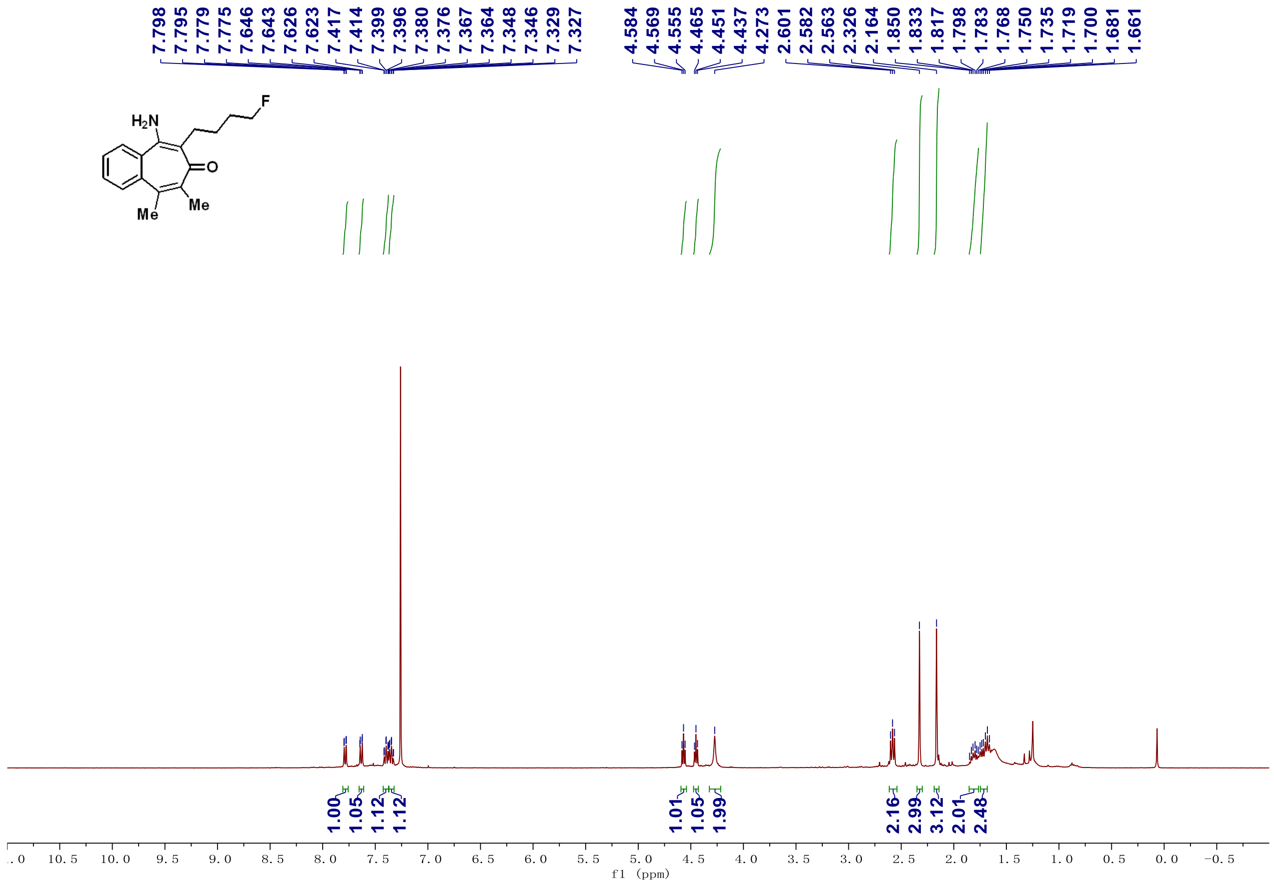

^{13}C **NMR**-spectrum (100 MHz, $CDCl_3$) of **3i**

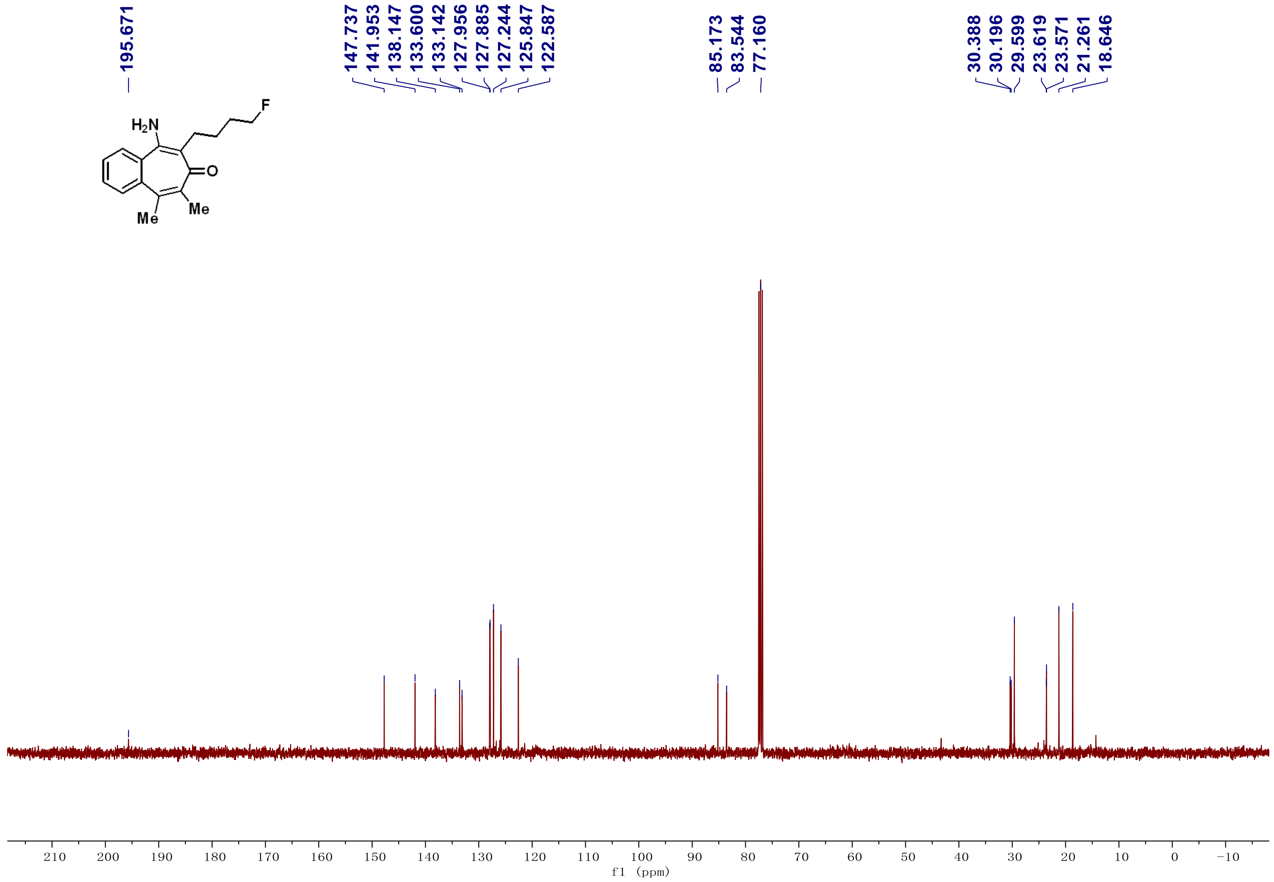

^{19}**F NMR**-spectrum (376 MHz, $CDCl_3$) of **3i**

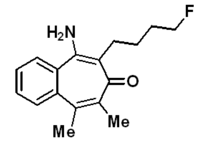

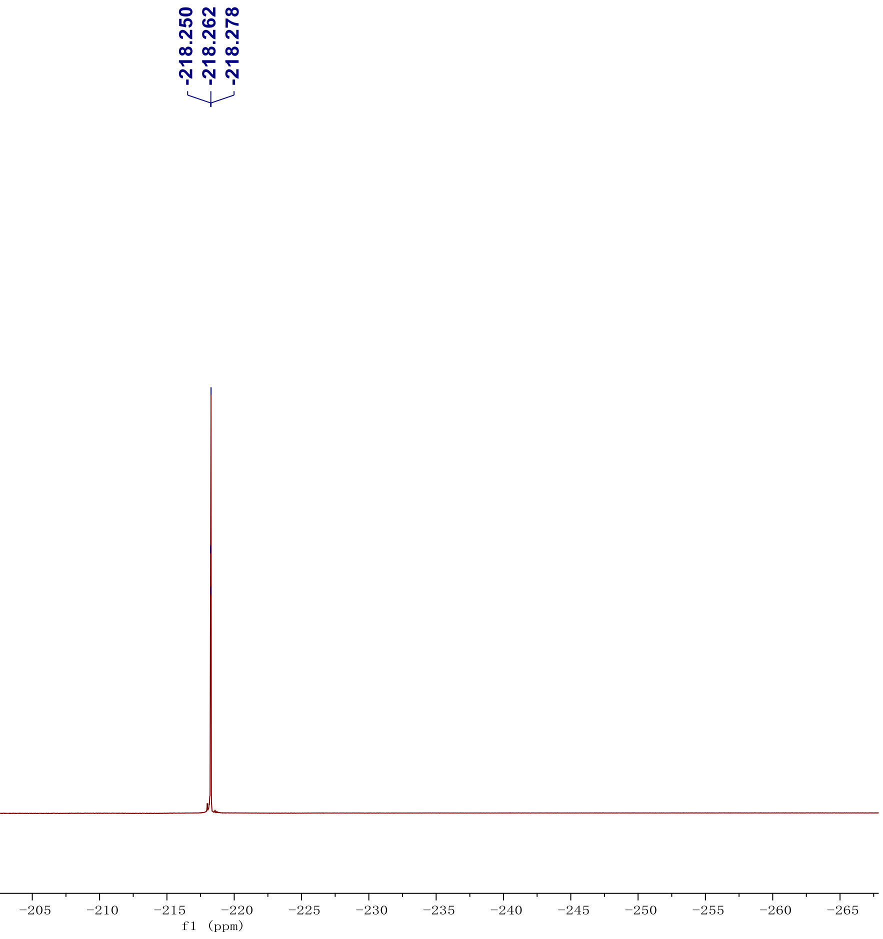

^{1}H NMR-spectrum (400 MHz, $CDCl_3$) of **3j**

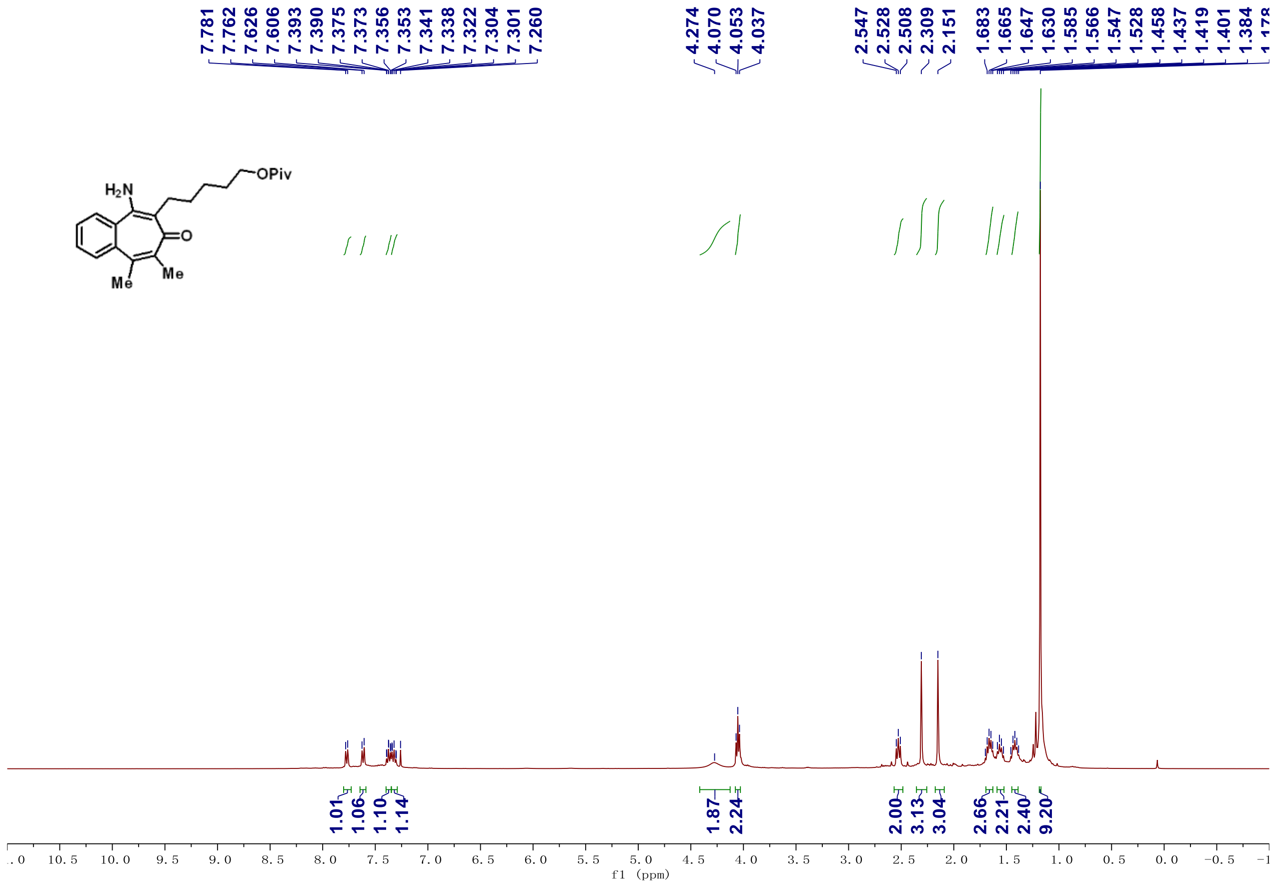

^{13}C NMR-spectrum (100 MHz, $CDCl_3$) of **3j**

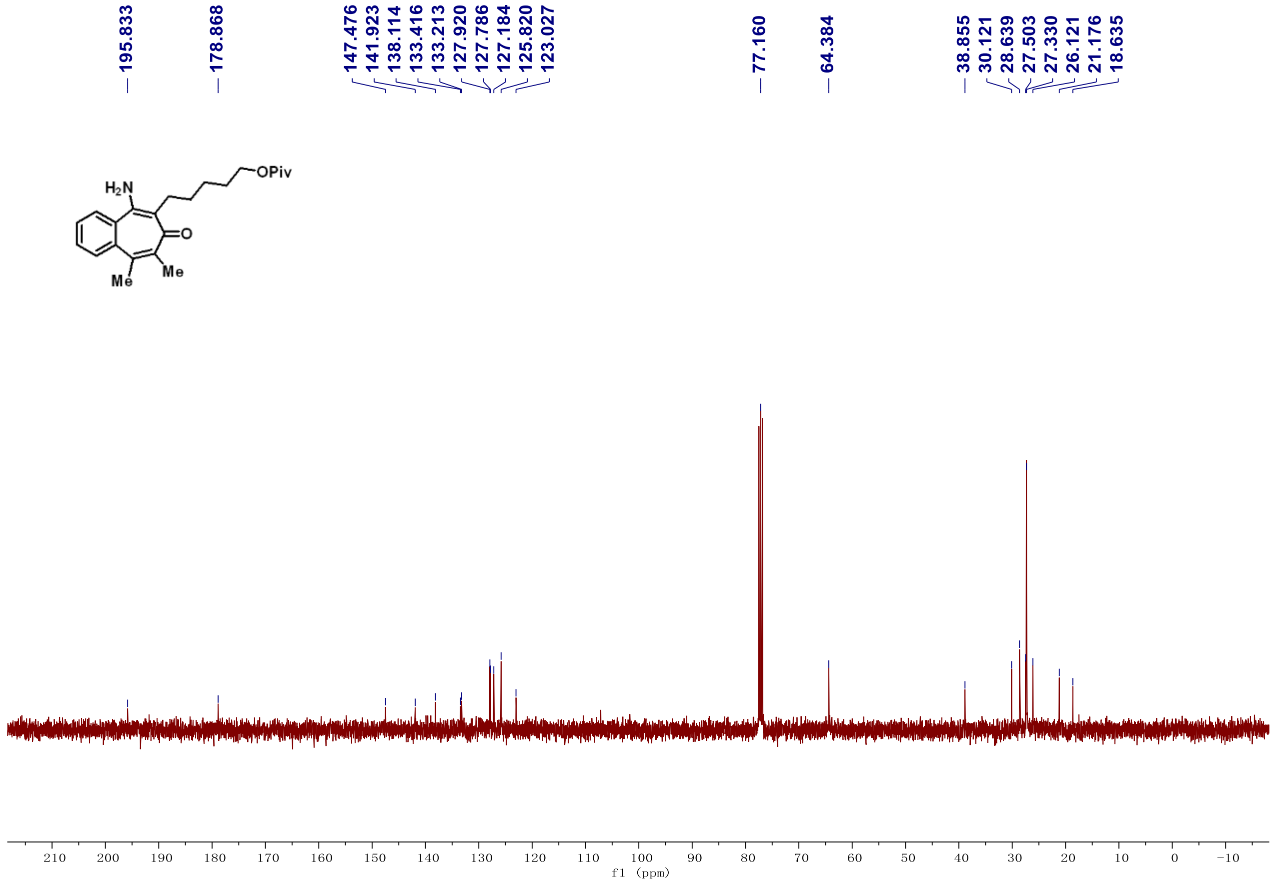

^{1}H NMR-spectrum (400 MHz, $CDCl_3$) of **3k**

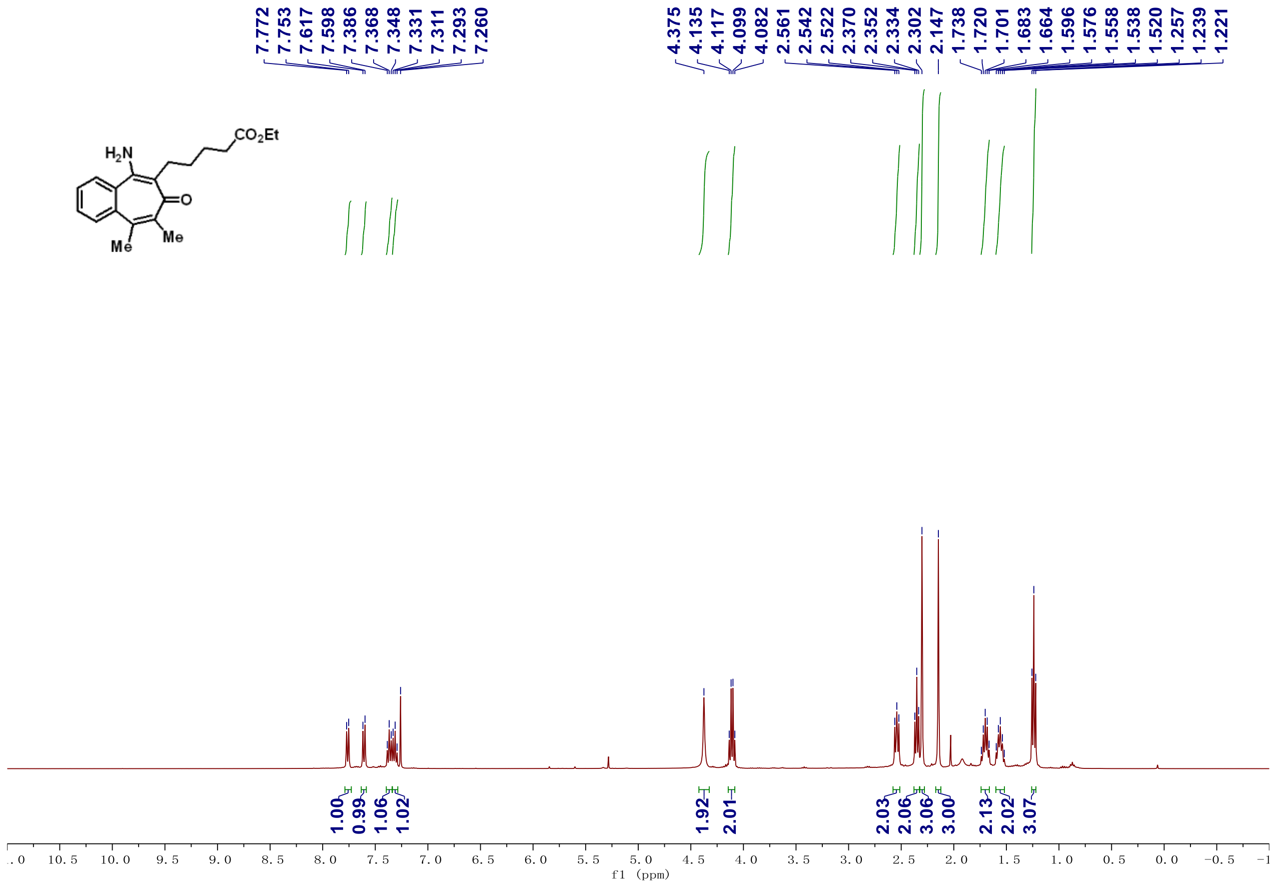

^{13}C NMR-spectrum (100 MHz, $CDCl_3$) of **3k**

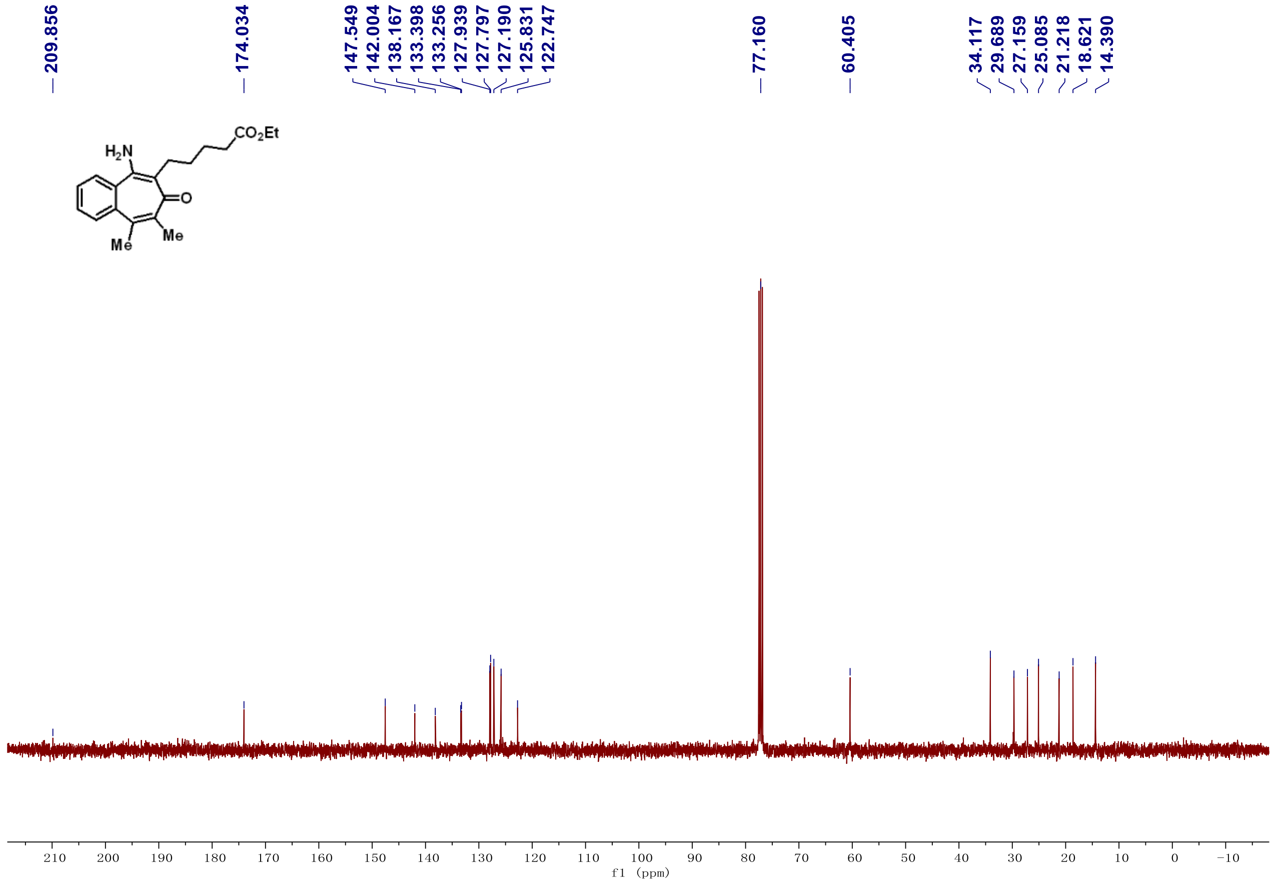

^{1}H NMR-spectrum (400 MHz, $CDCl_3$) of **3l**

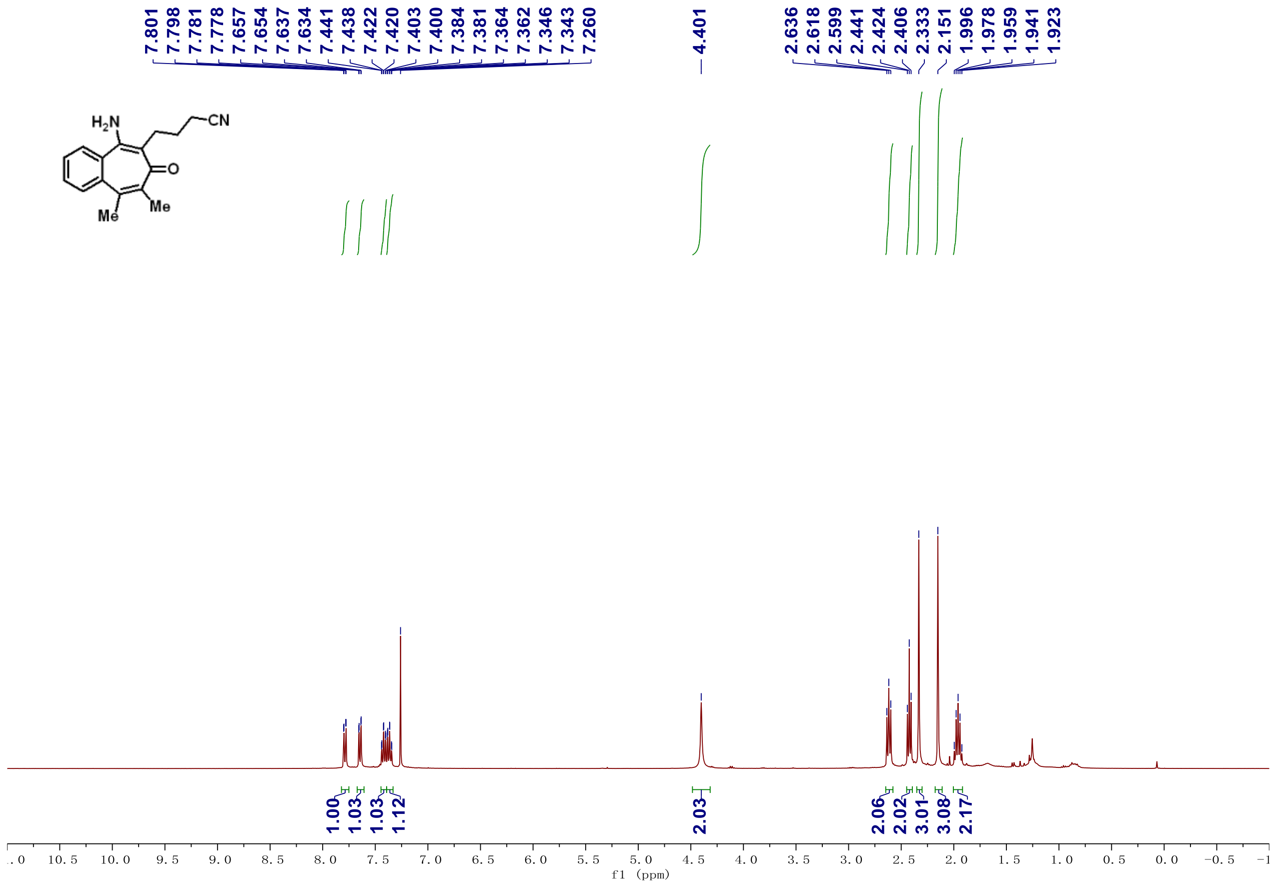

^{13}C NMR-spectrum (100 MHz, $CDCl_3$) of **3l**

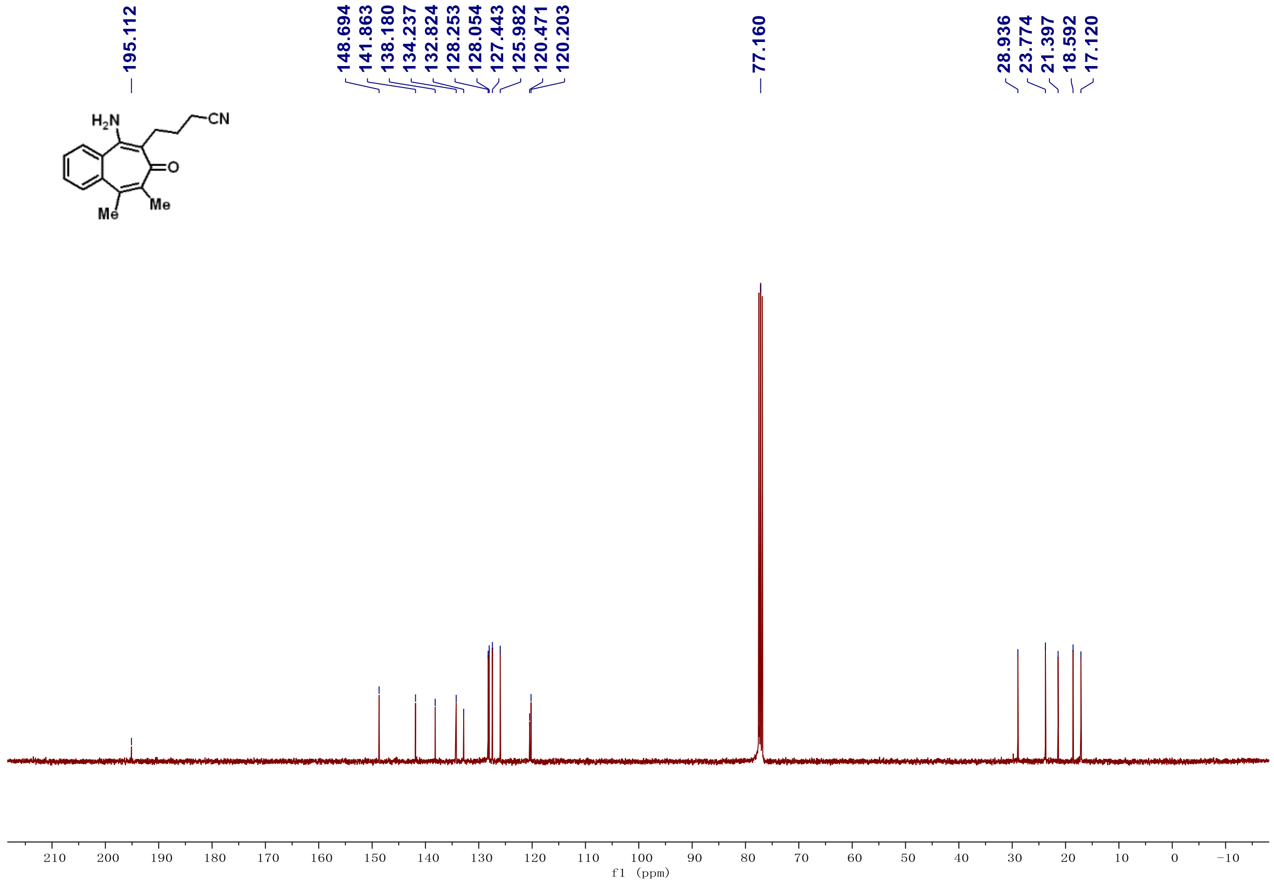

^{1}H NMR-spectrum (400 MHz, $CDCl_3$) of **3m**

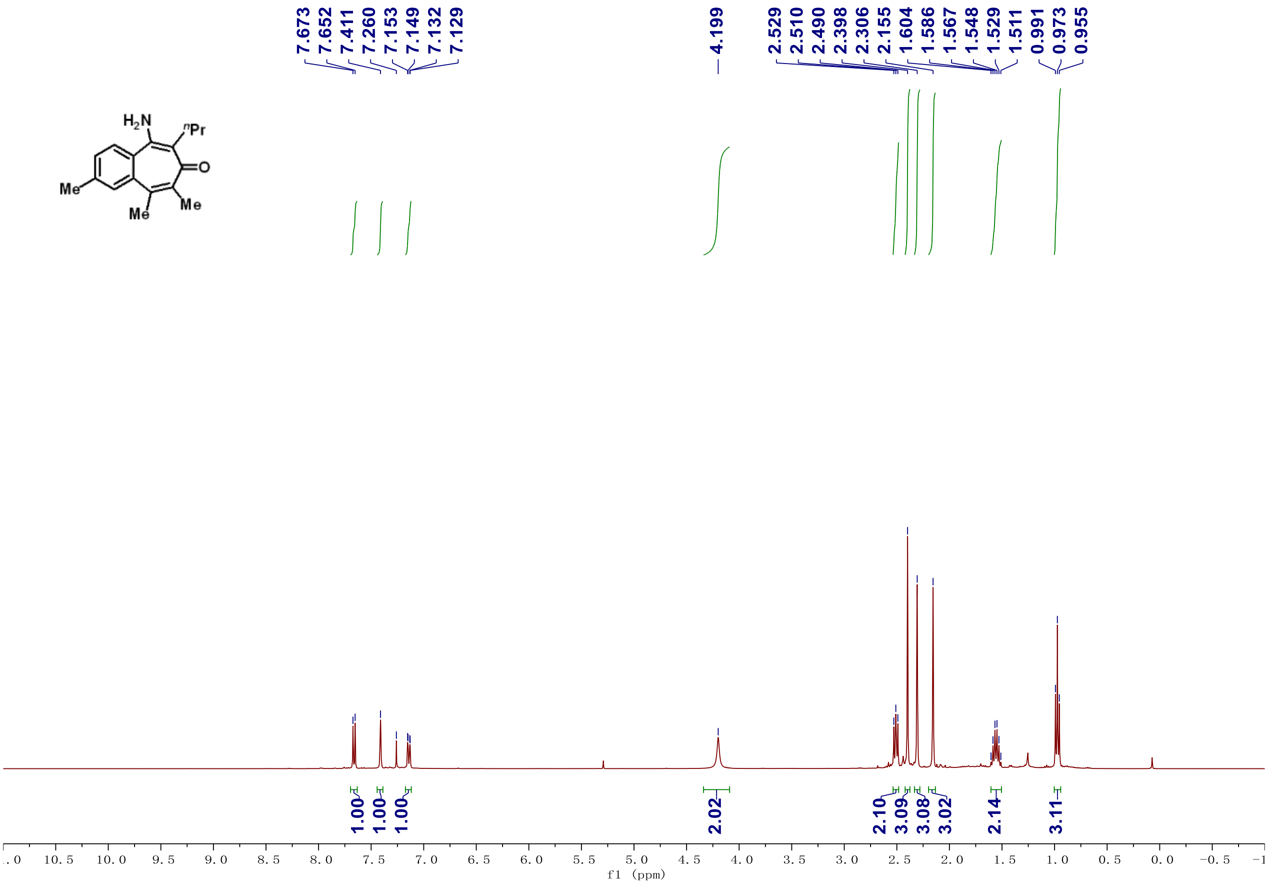

^{13}C NMR-spectrum (100 MHz, $CDCl_3$) of **3m**

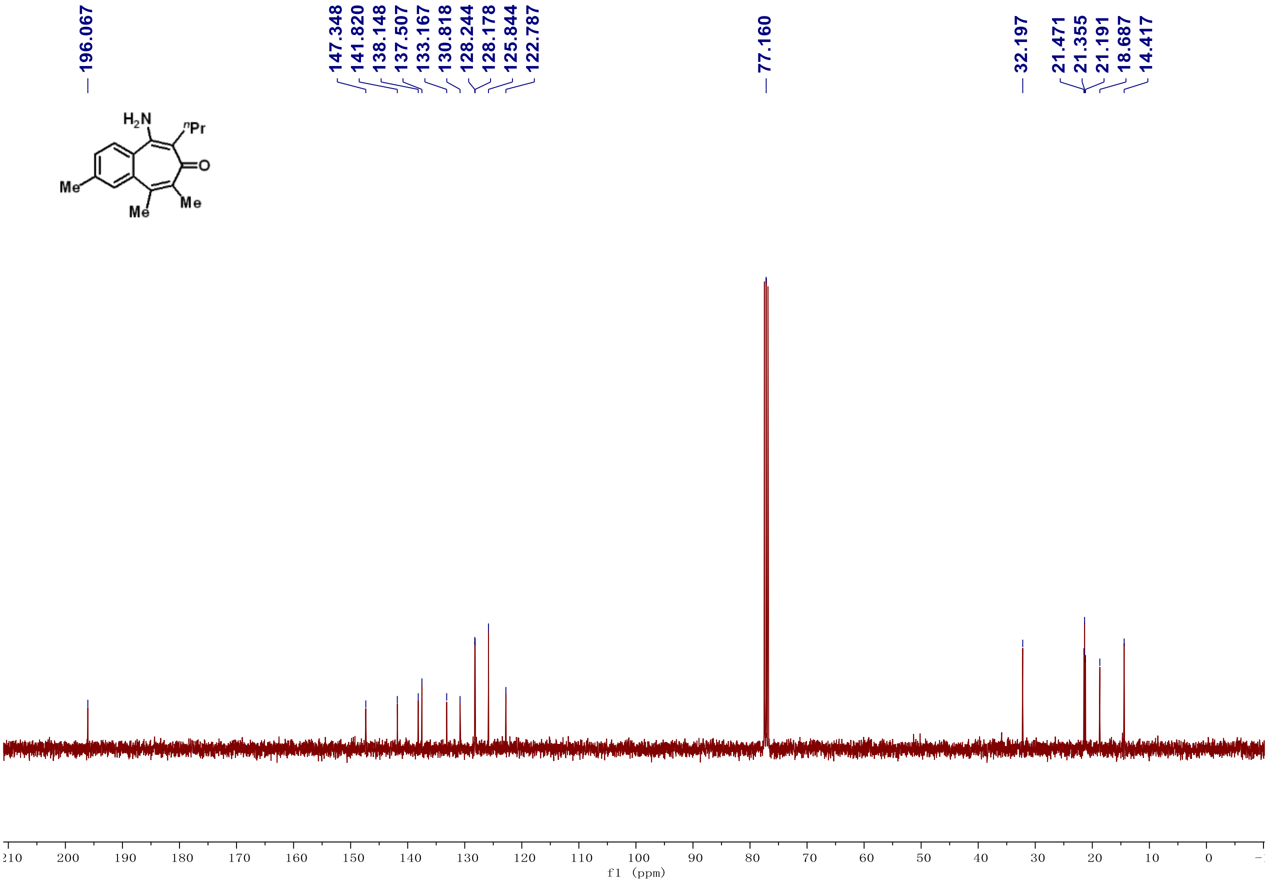

^{1}H NMR-spectrum (400 MHz, $CDCl_3$) of **3n**

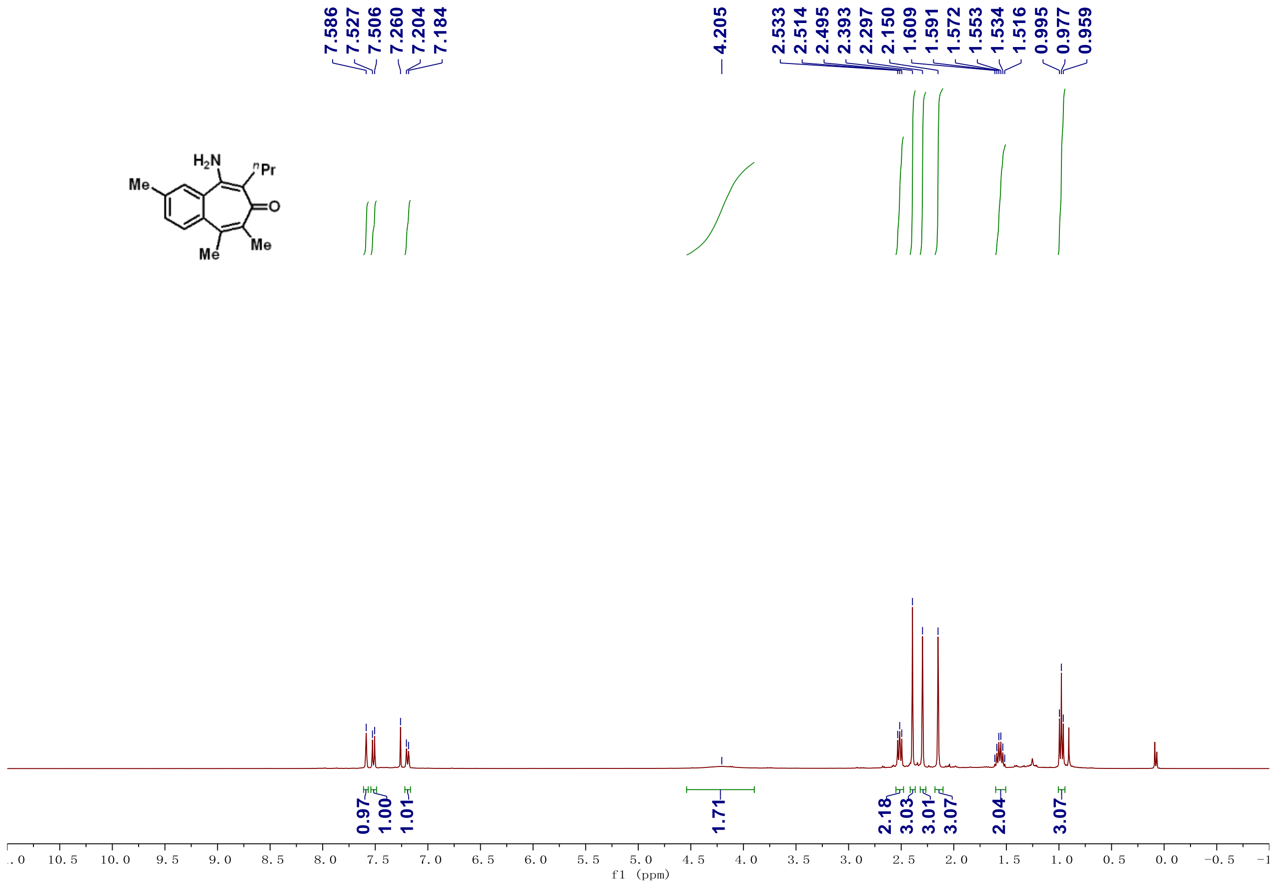

^{13}C NMR-spectrum (100 MHz, $CDCl_3$) of **3n**

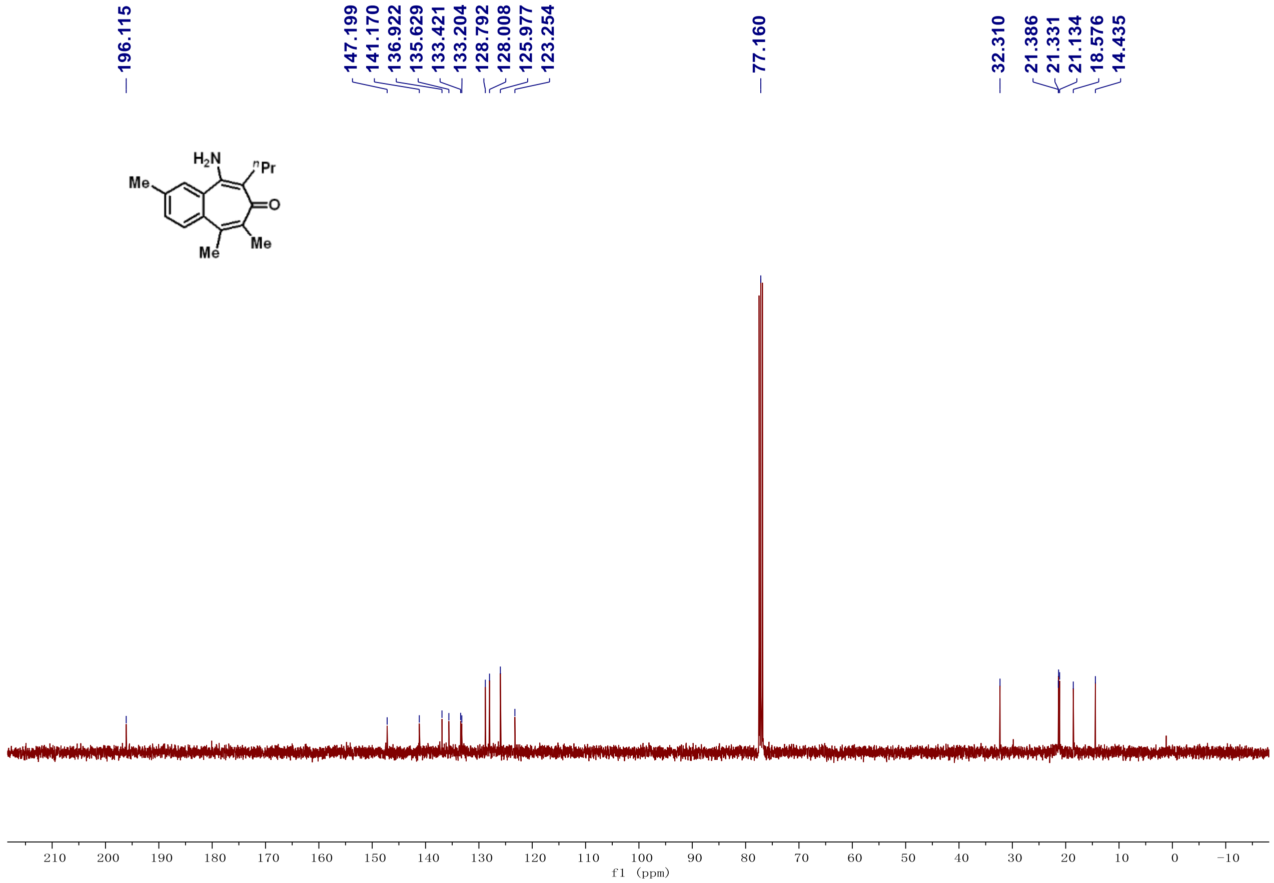

^{1}H NMR-spectrum (400 MHz, $CDCl_3$) of **3o**

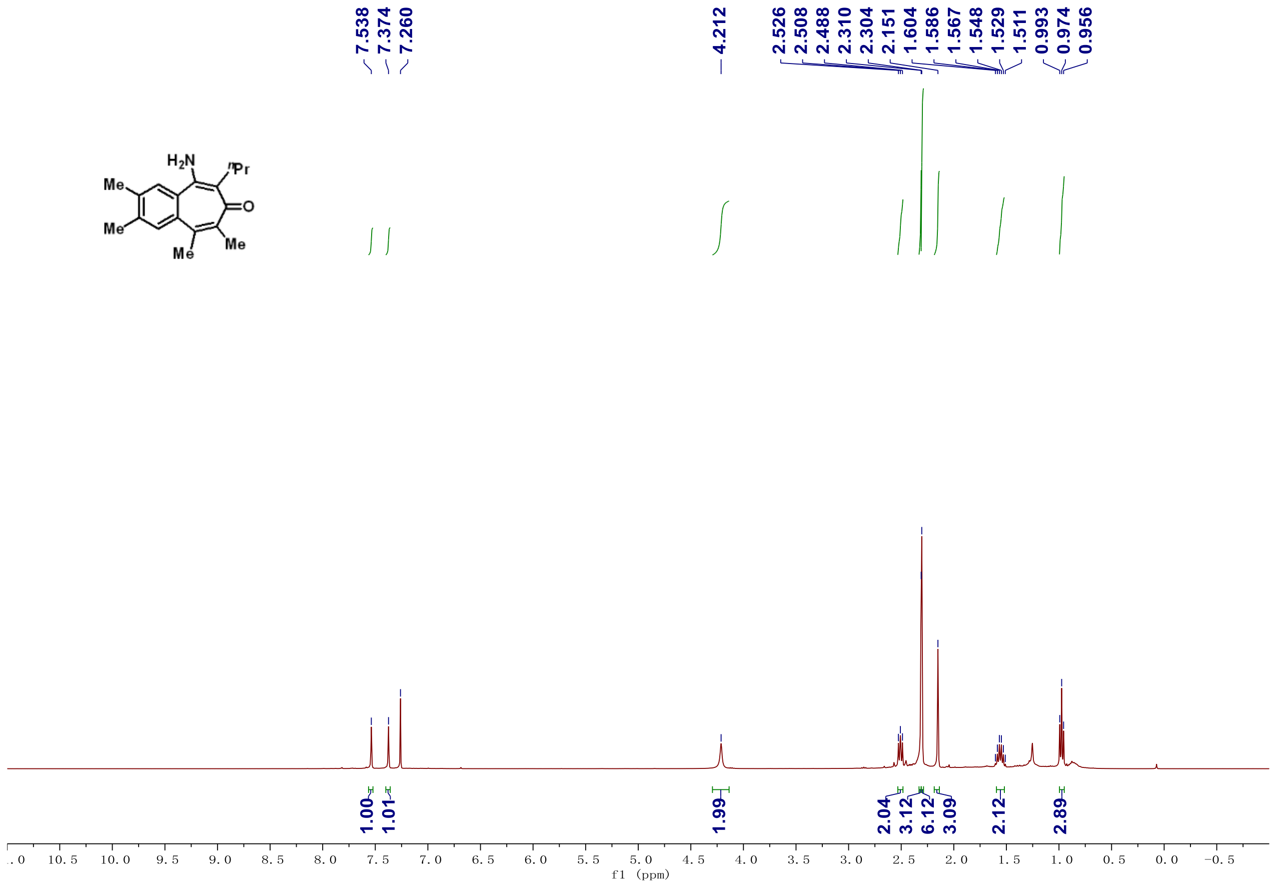

^{13}C NMR-spectrum (100 MHz, $CDCl_3$) of **3o**

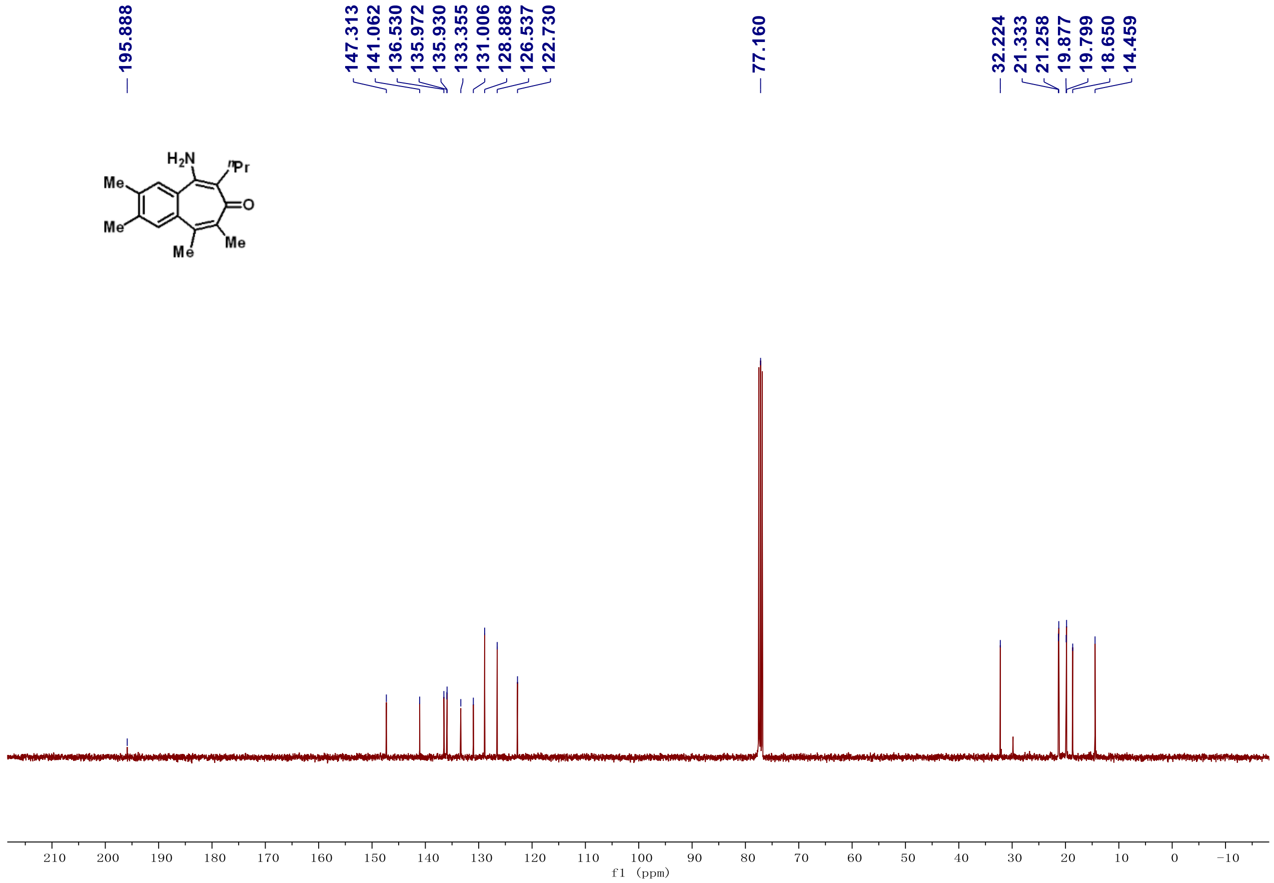

^{1}H NMR-spectrum (400 MHz, $CDCl_3$) of **3p**

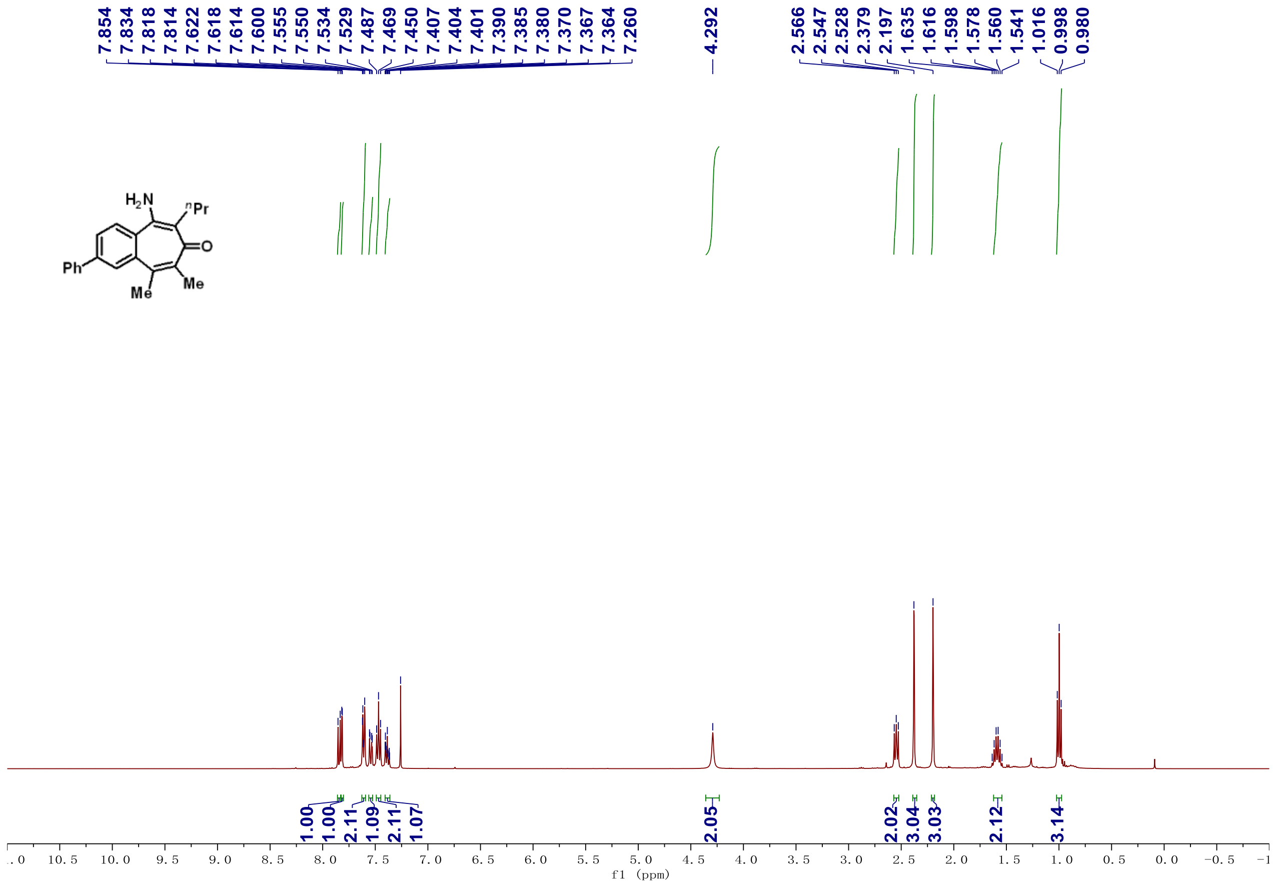

^{13}C NMR-spectrum (100 MHz, $CDCl_3$) of **3p**

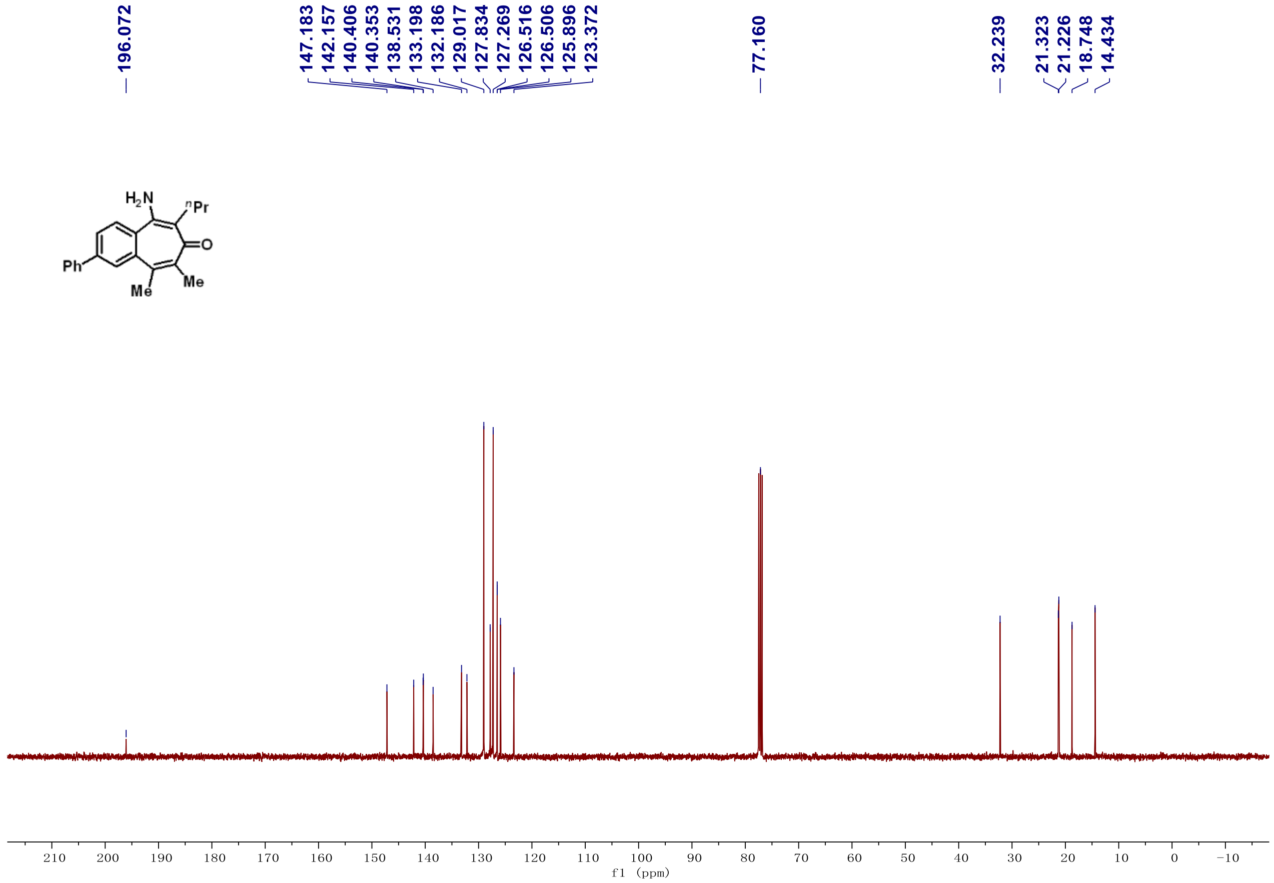

^{1}H NMR-spectrum (400 MHz, $CDCl_3$) of **3q**

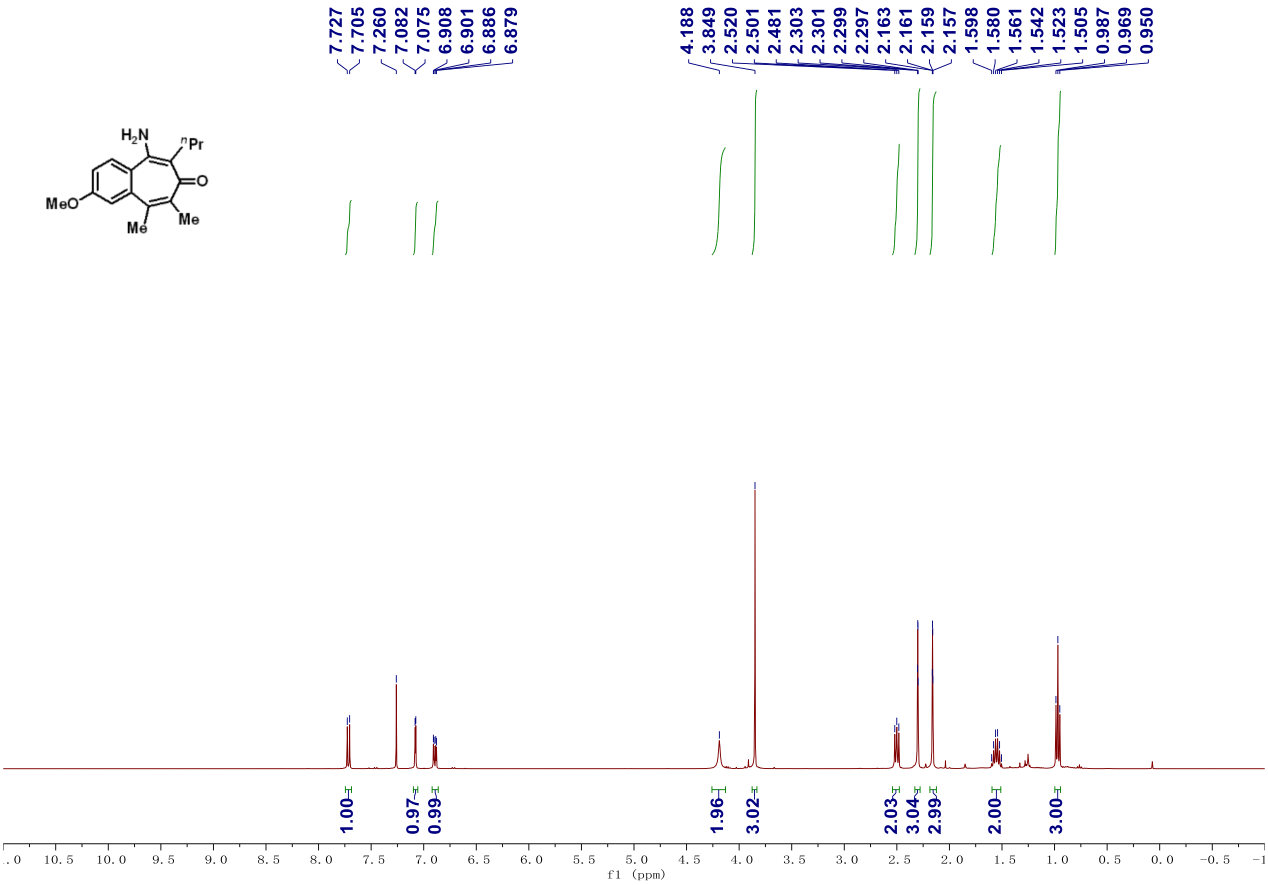

^{13}C NMR-spectrum (100 MHz, $CDCl_3$) of **3q**

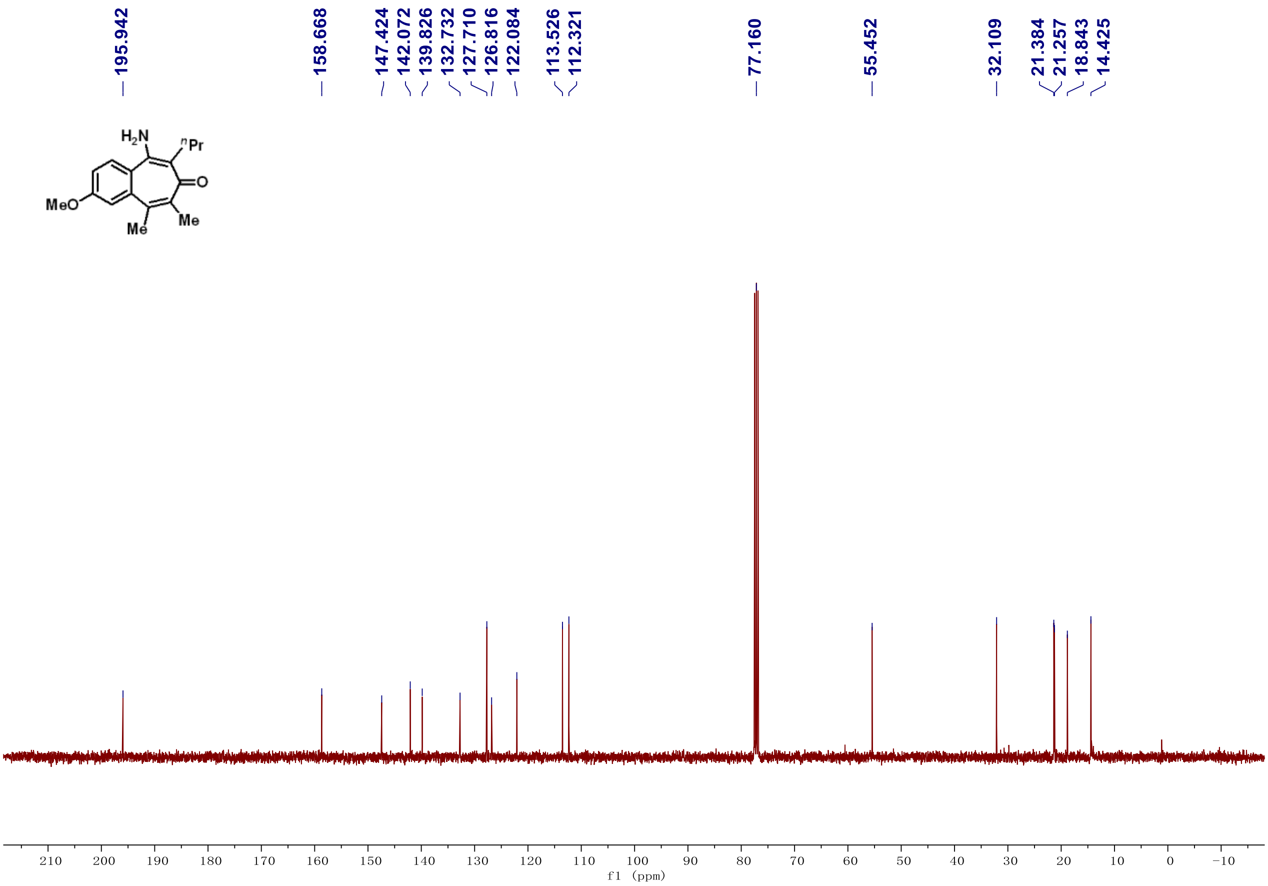

^{1}H NMR-spectrum (400 MHz, $CDCl_3$) of **3r**

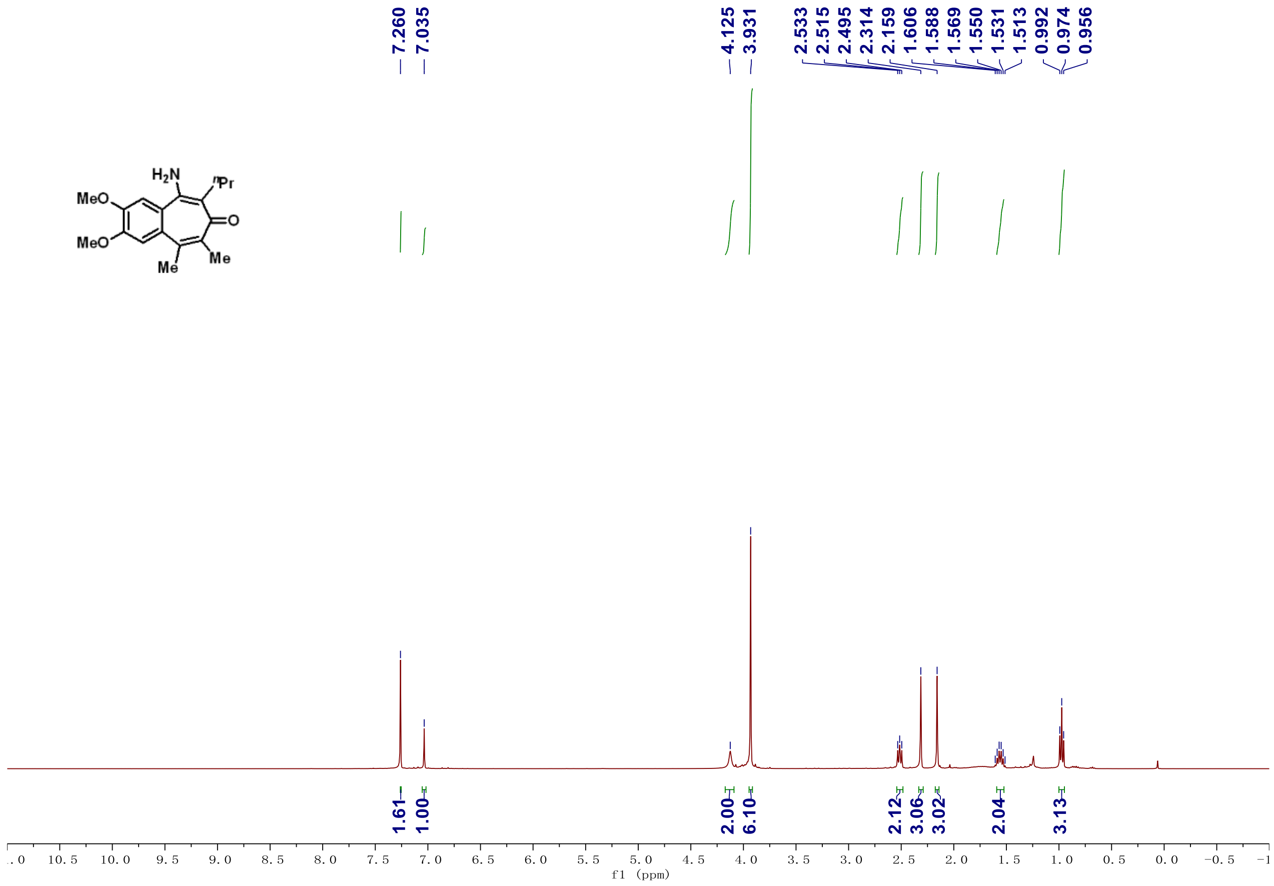

^{13}C NMR-spectrum (100 MHz, $CDCl_3$) of **3r**

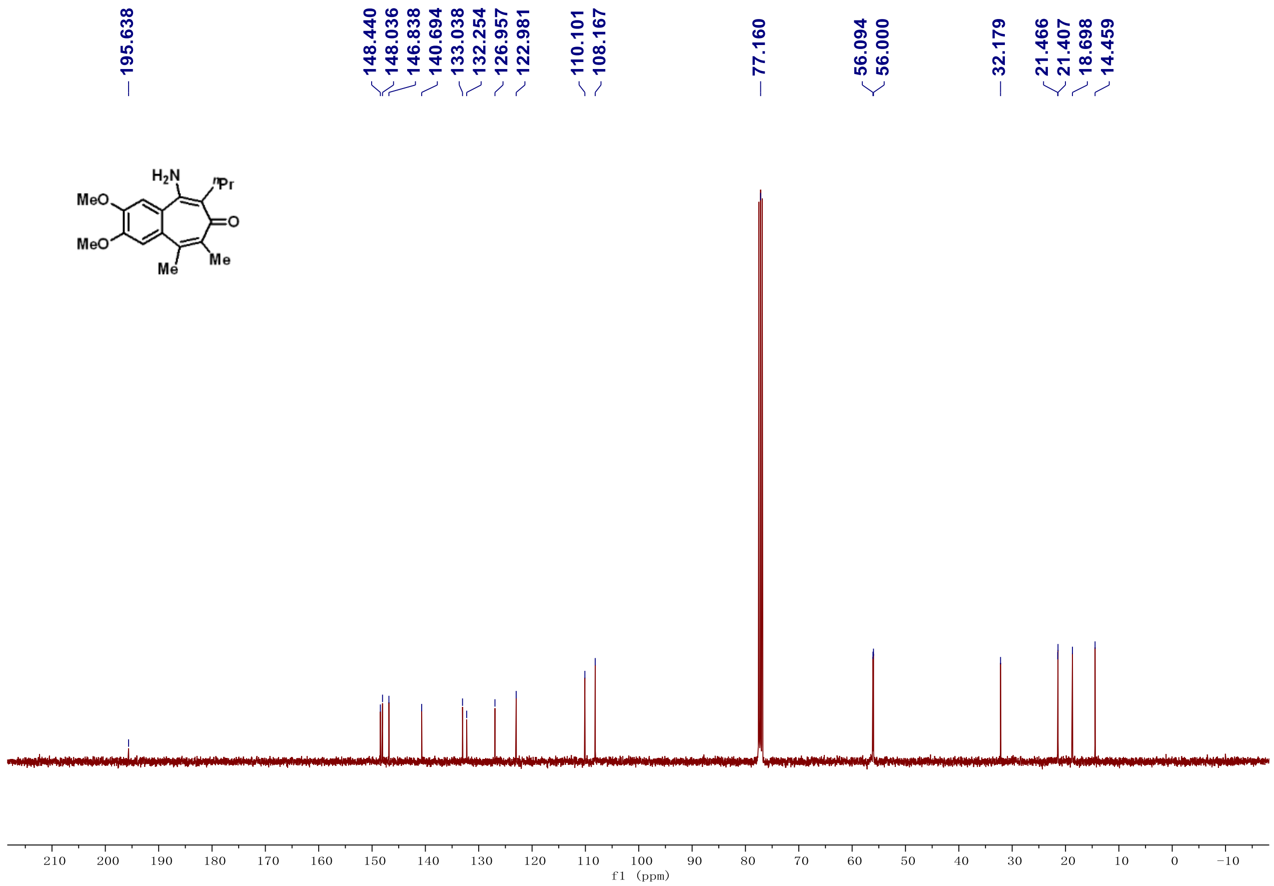

^{1}H NMR-spectrum (400 MHz, $CDCl_3$) of **3s**

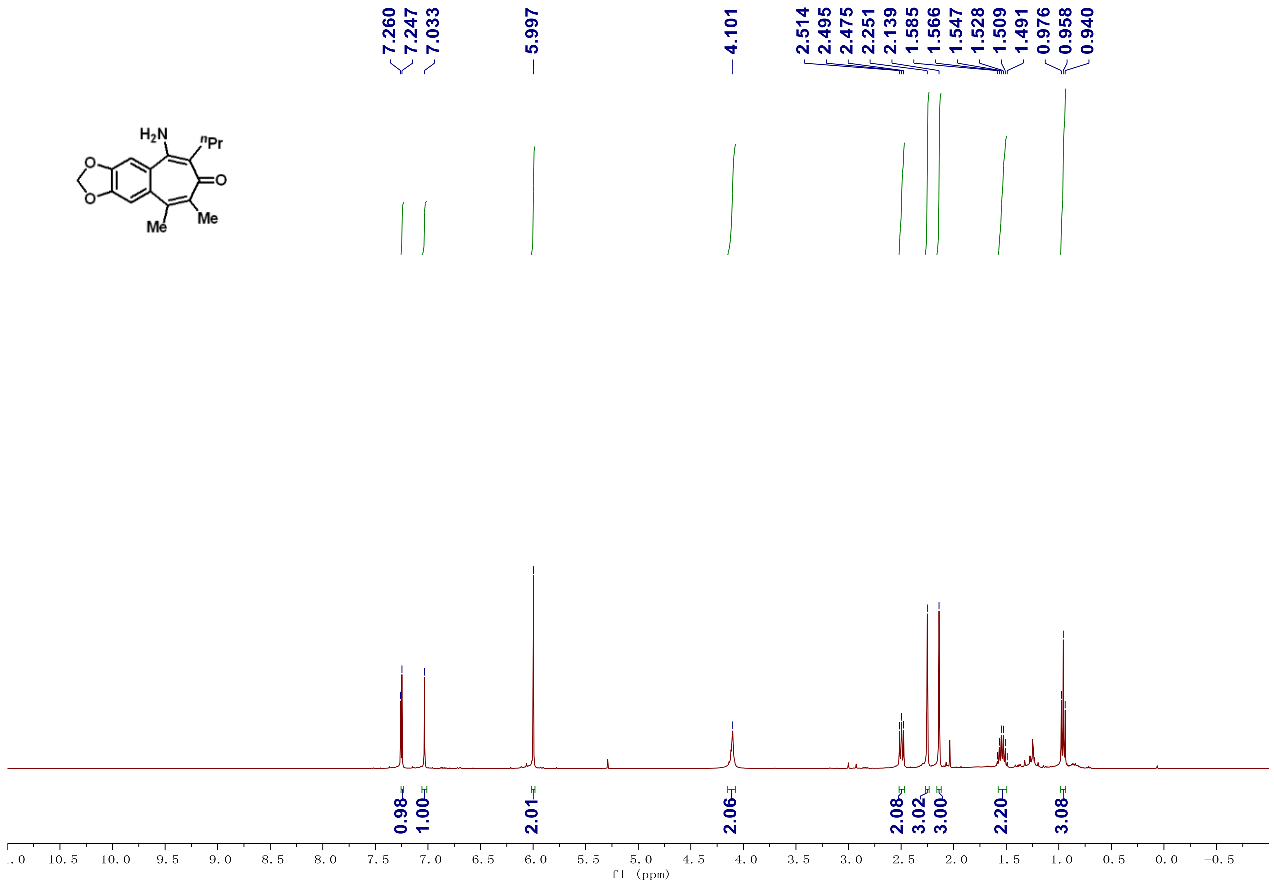

^{13}C NMR-spectrum (100 MHz, $CDCl_3$) of **3s**

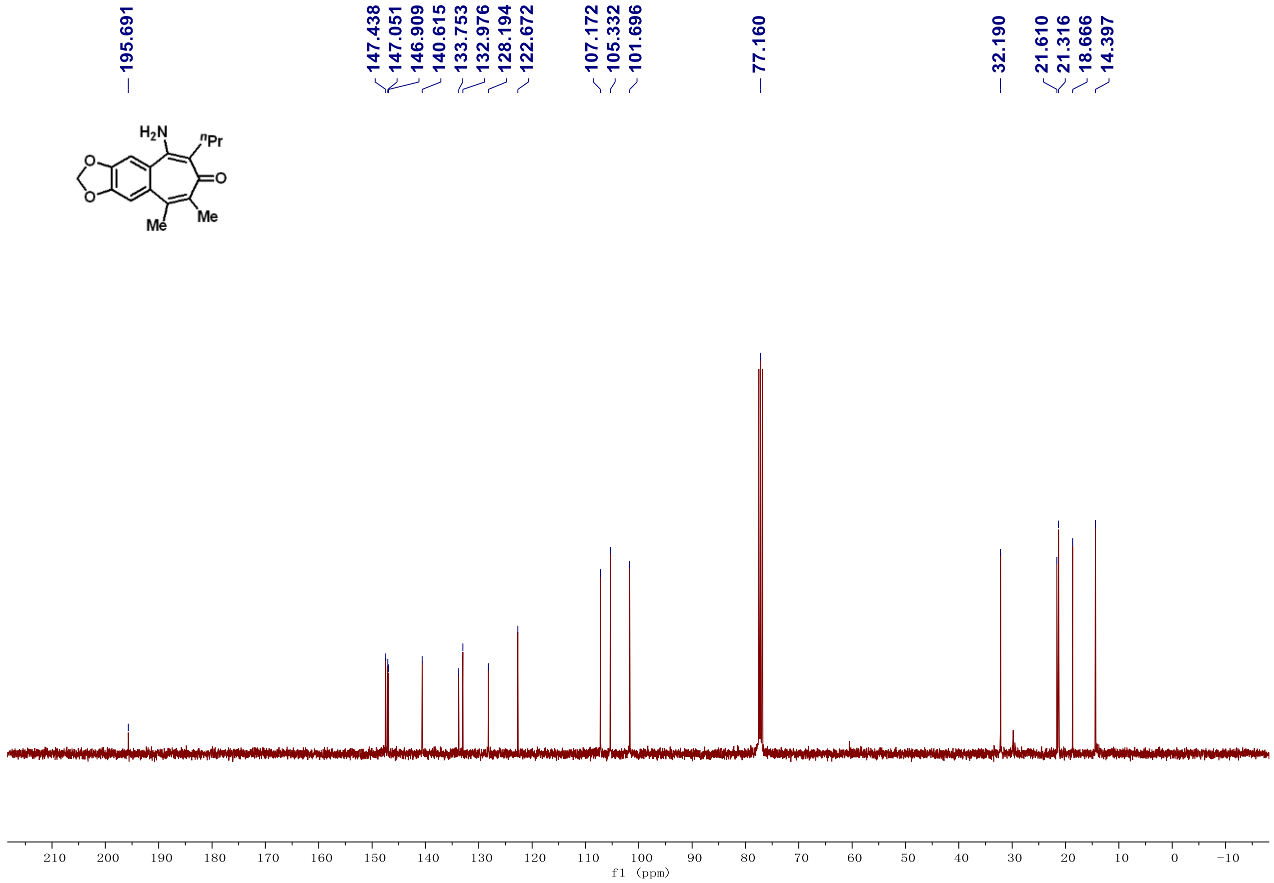

[1]**H NMR**-spectrum (400 MHz, $CDCl_3$) of **3t**

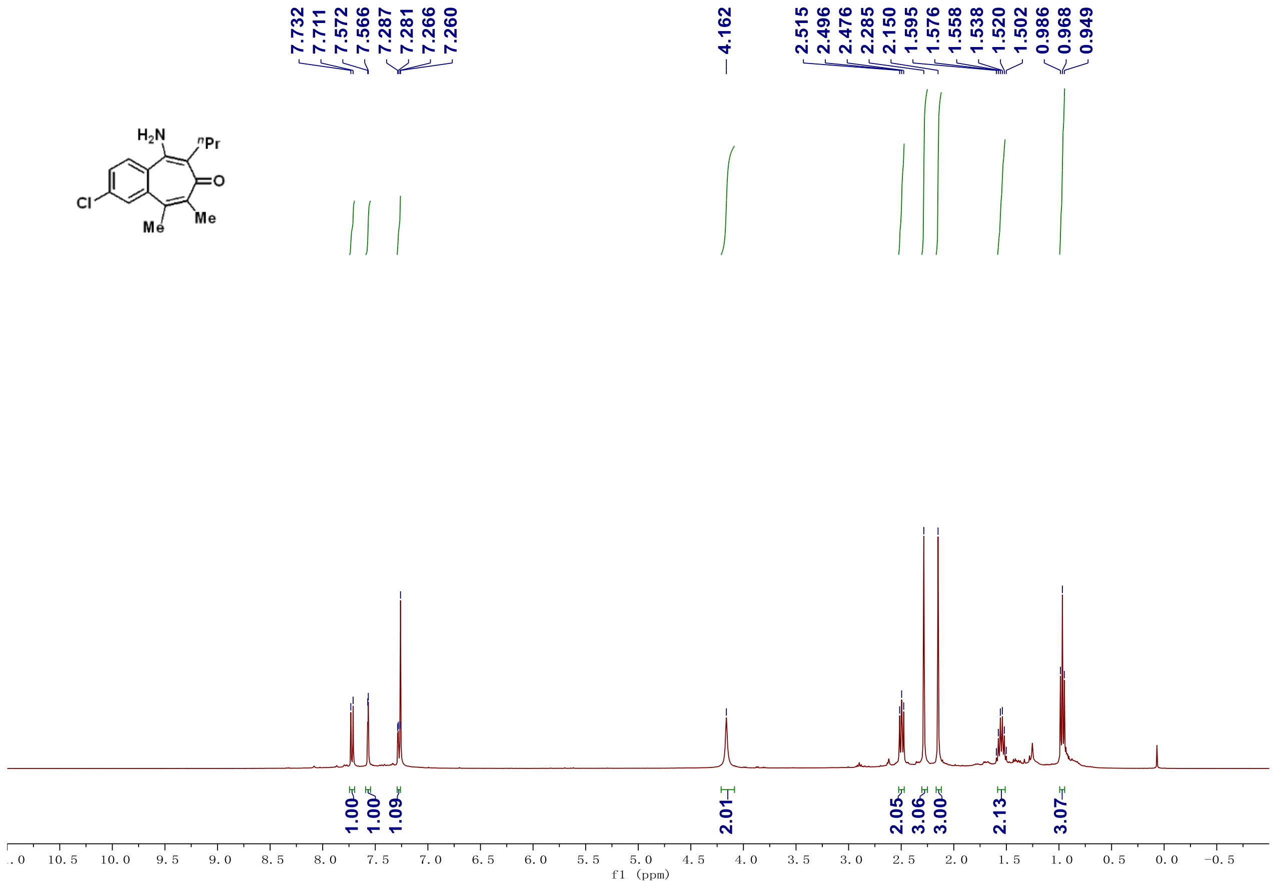

[13]**C NMR**-spectrum (100 MHz, $CDCl_3$) of **3t**

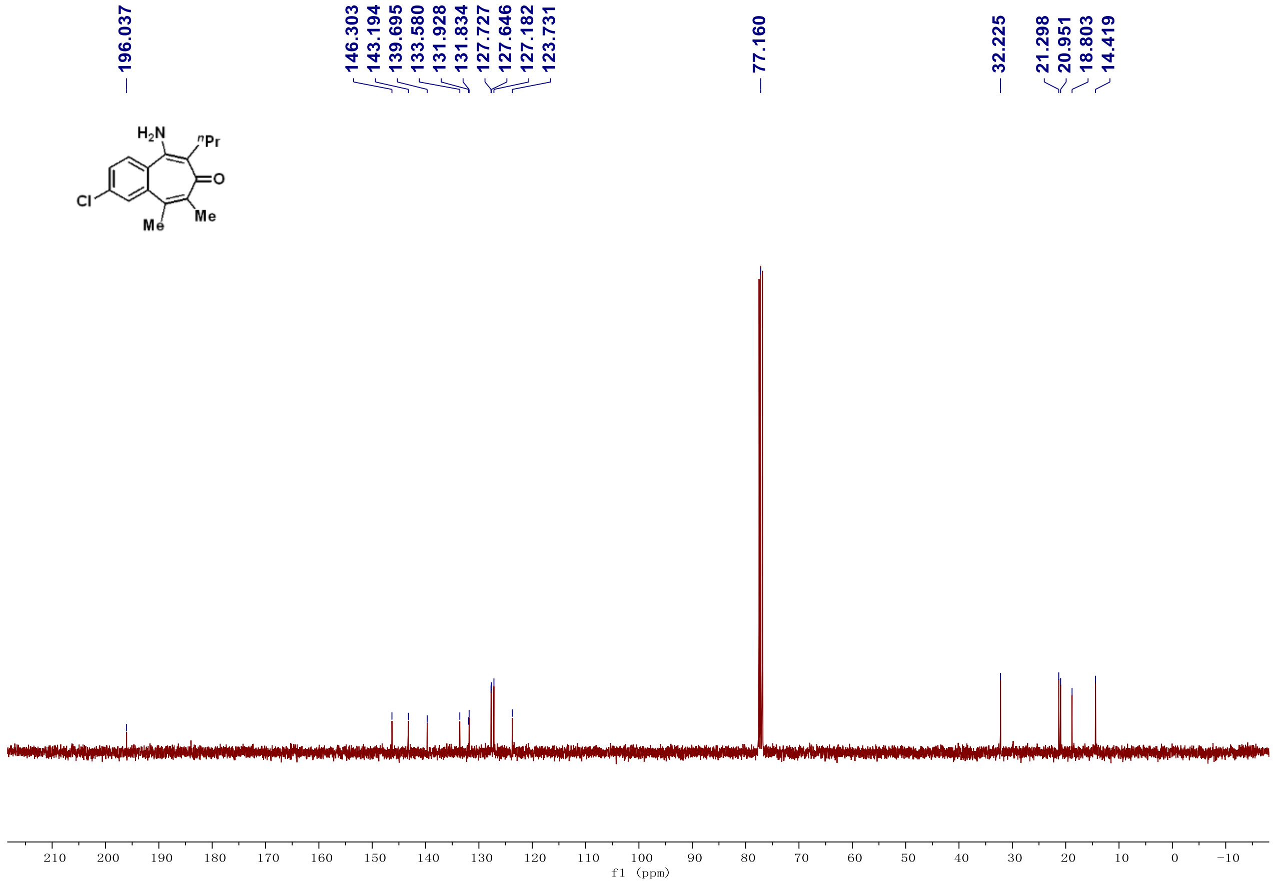

^{1}H NMR-spectrum (400 MHz, $CDCl_3$) of **3u**

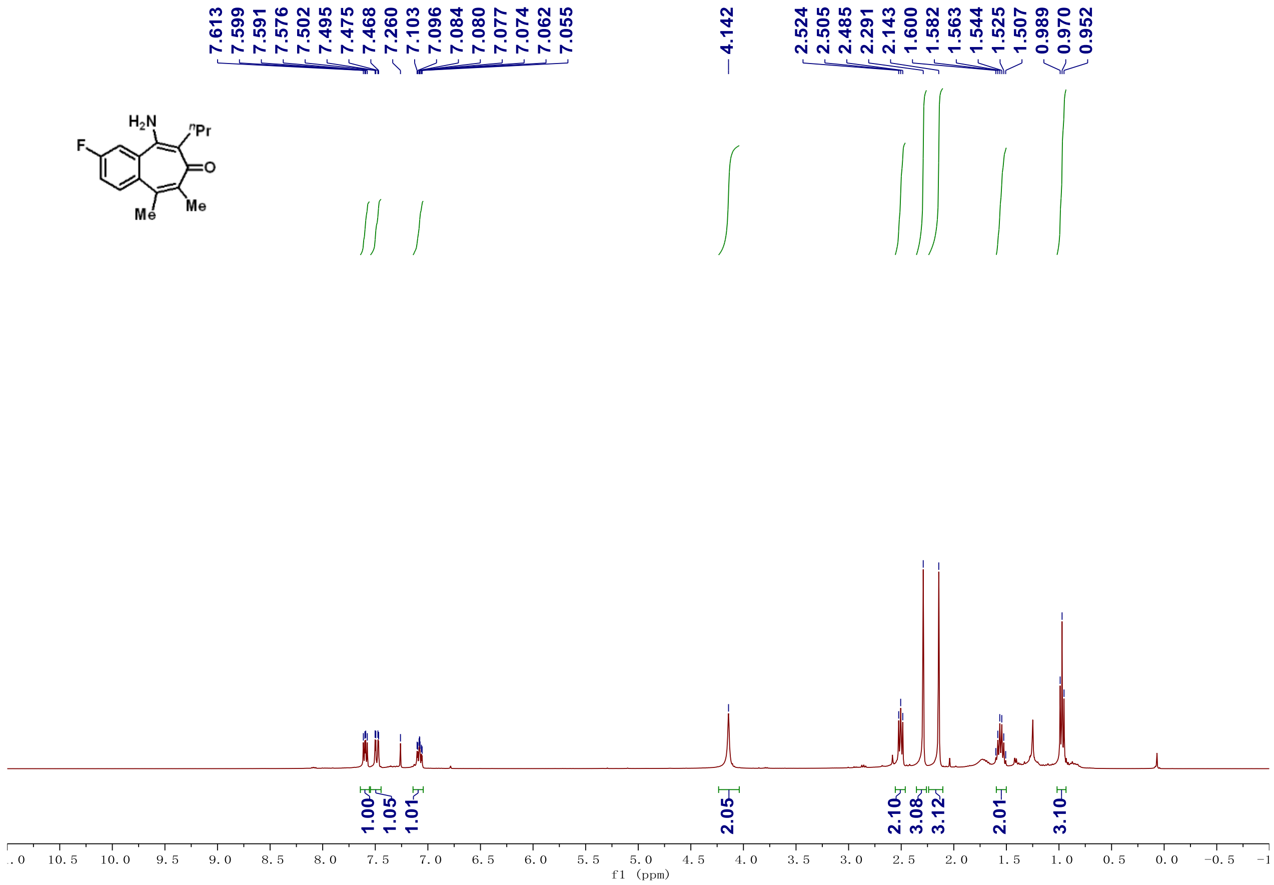

^{13}C NMR-spectrum (100 MHz, $CDCl_3$) of **3u**

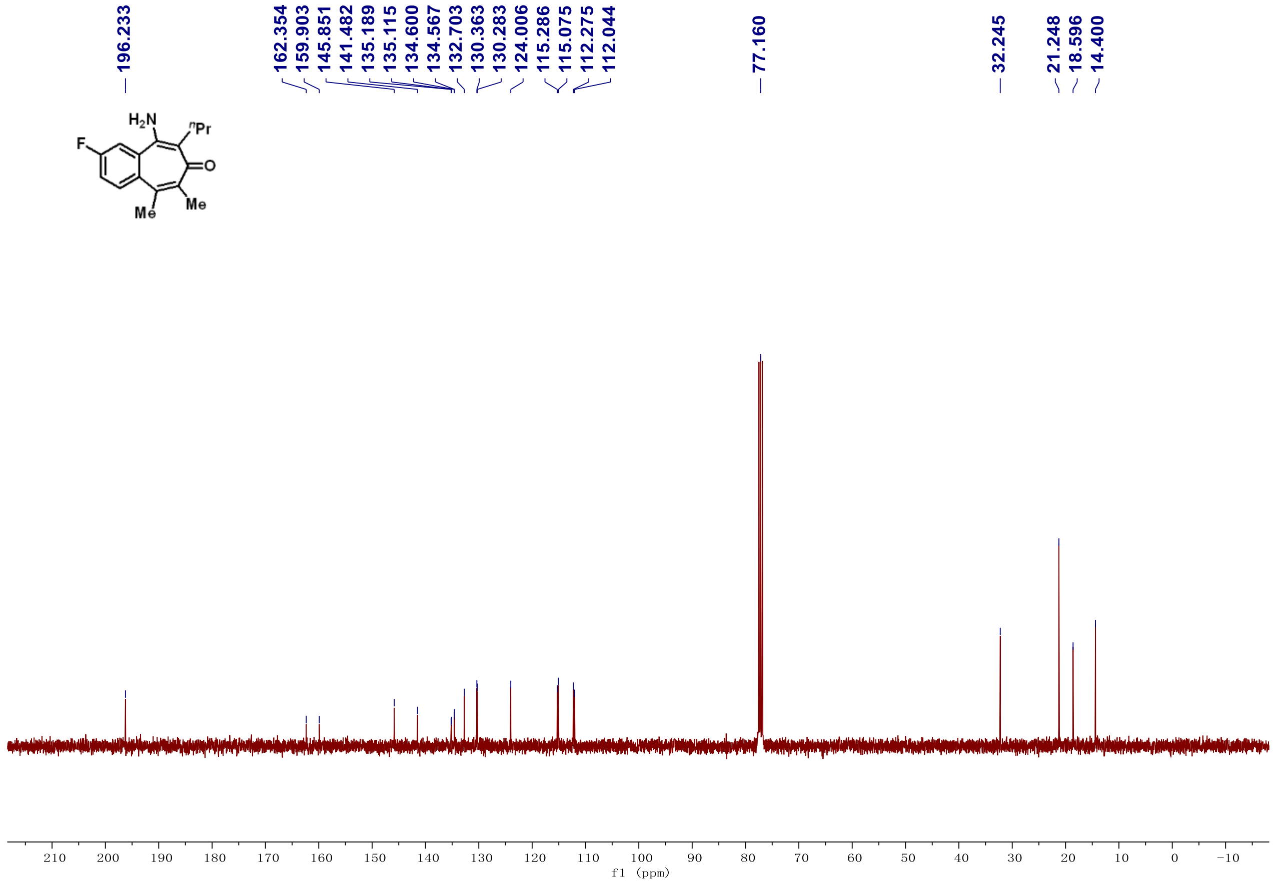

^{1}H NMR-spectrum (400 MHz, $CDCl_3$) of **3v**

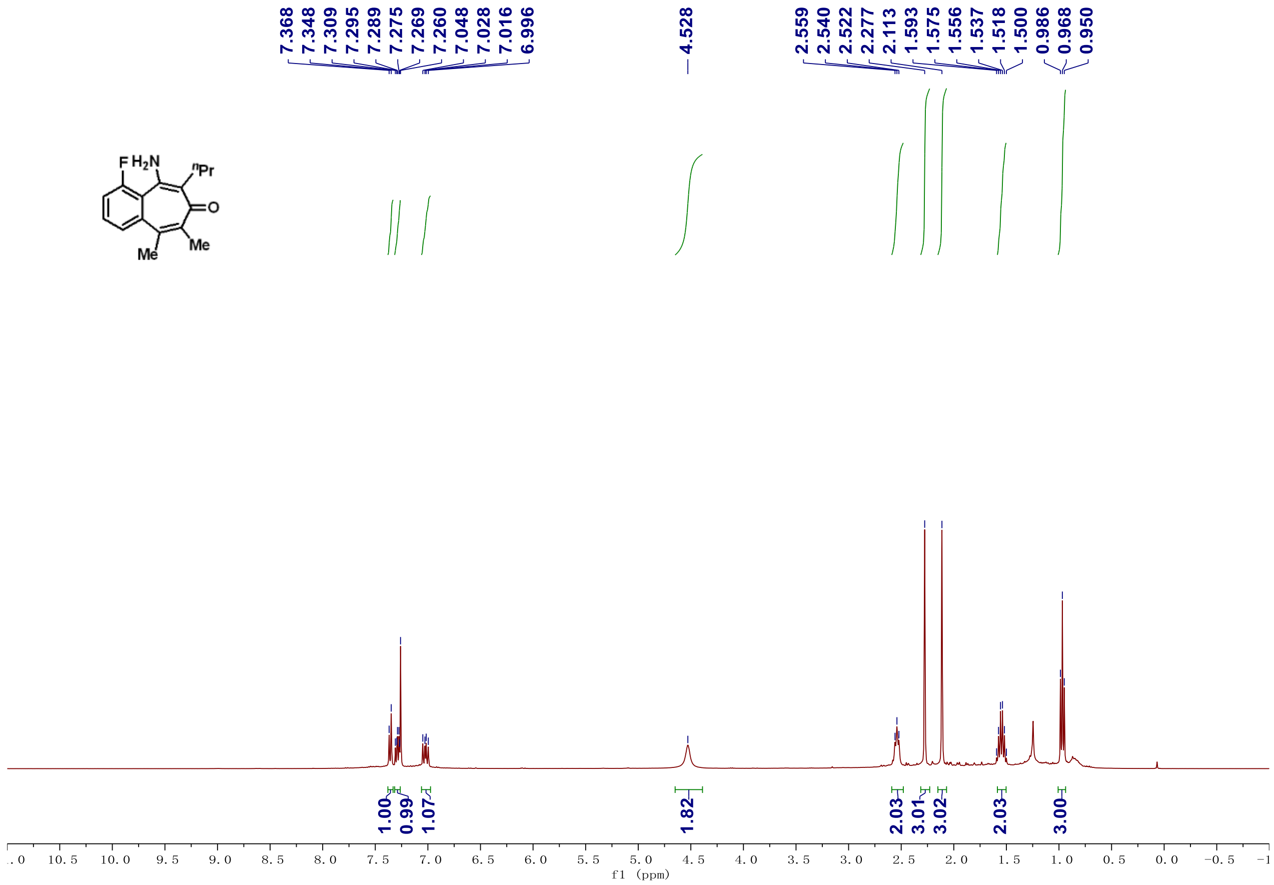

^{13}C NMR-spectrum (100 MHz, $CDCl_3$) of **3v**

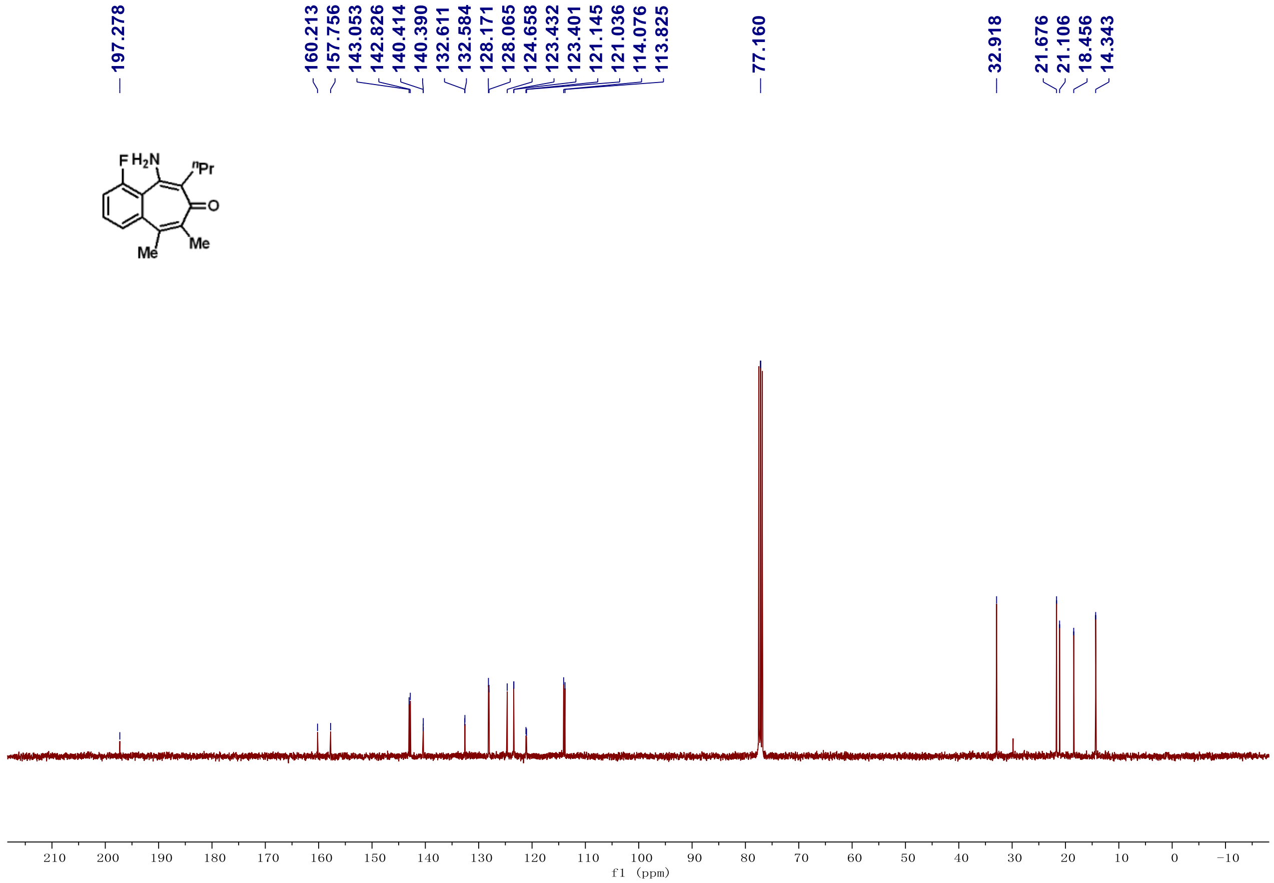

^{1}H NMR-spectrum (400 MHz, $CDCl_3$) of **3w**

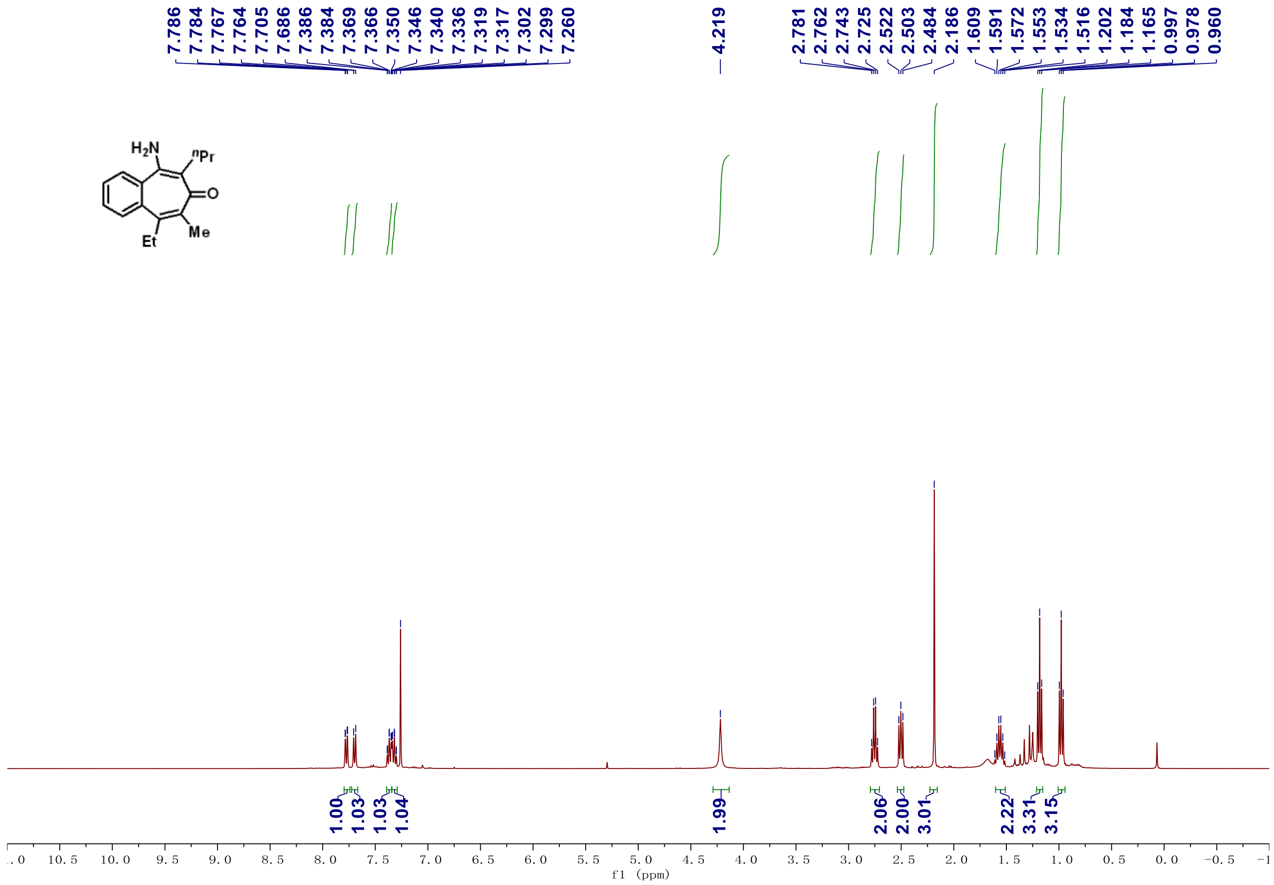

^{13}C NMR-spectrum (100 MHz, $CDCl_3$) of **3w**

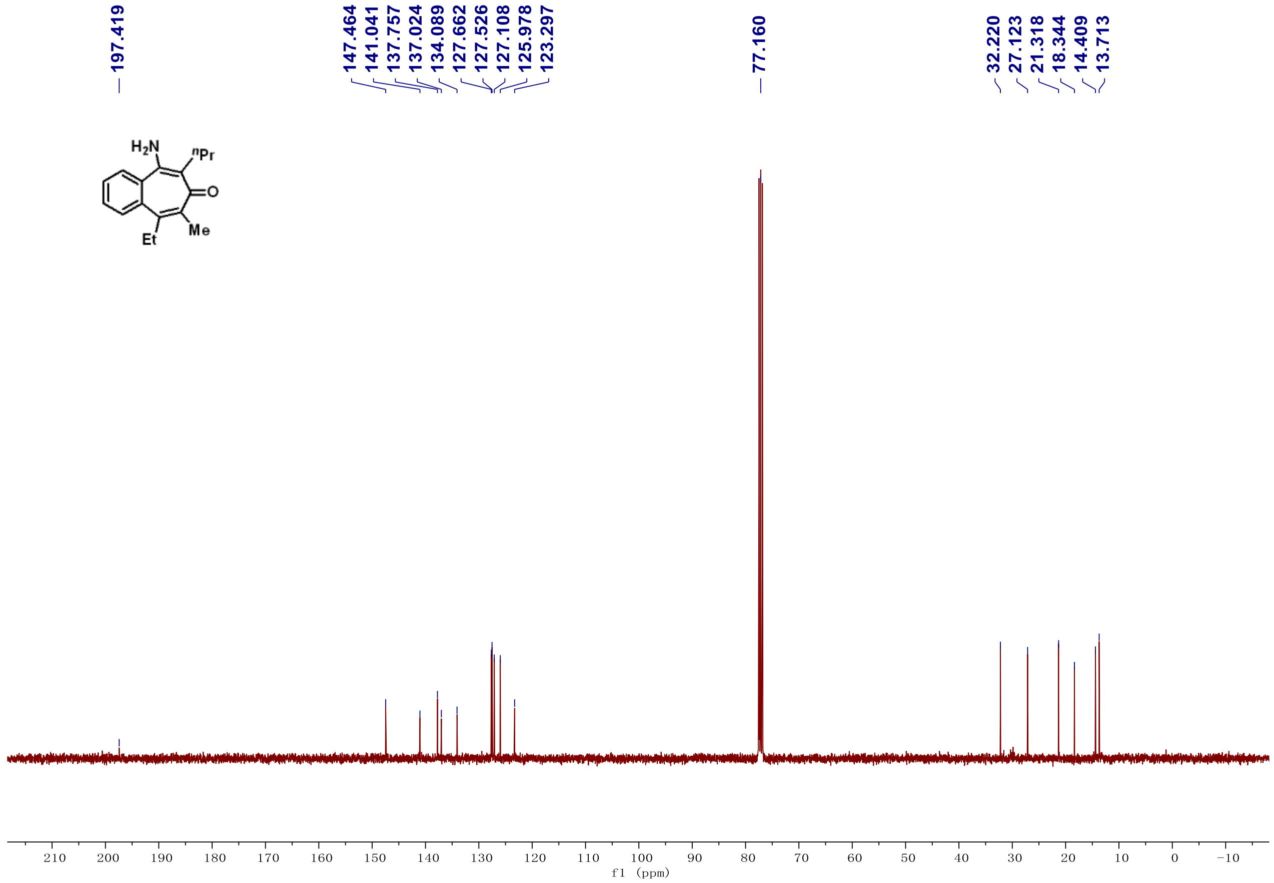

^{1}**H NMR**-spectrum (400 MHz, $CDCl_3$) of **3x**

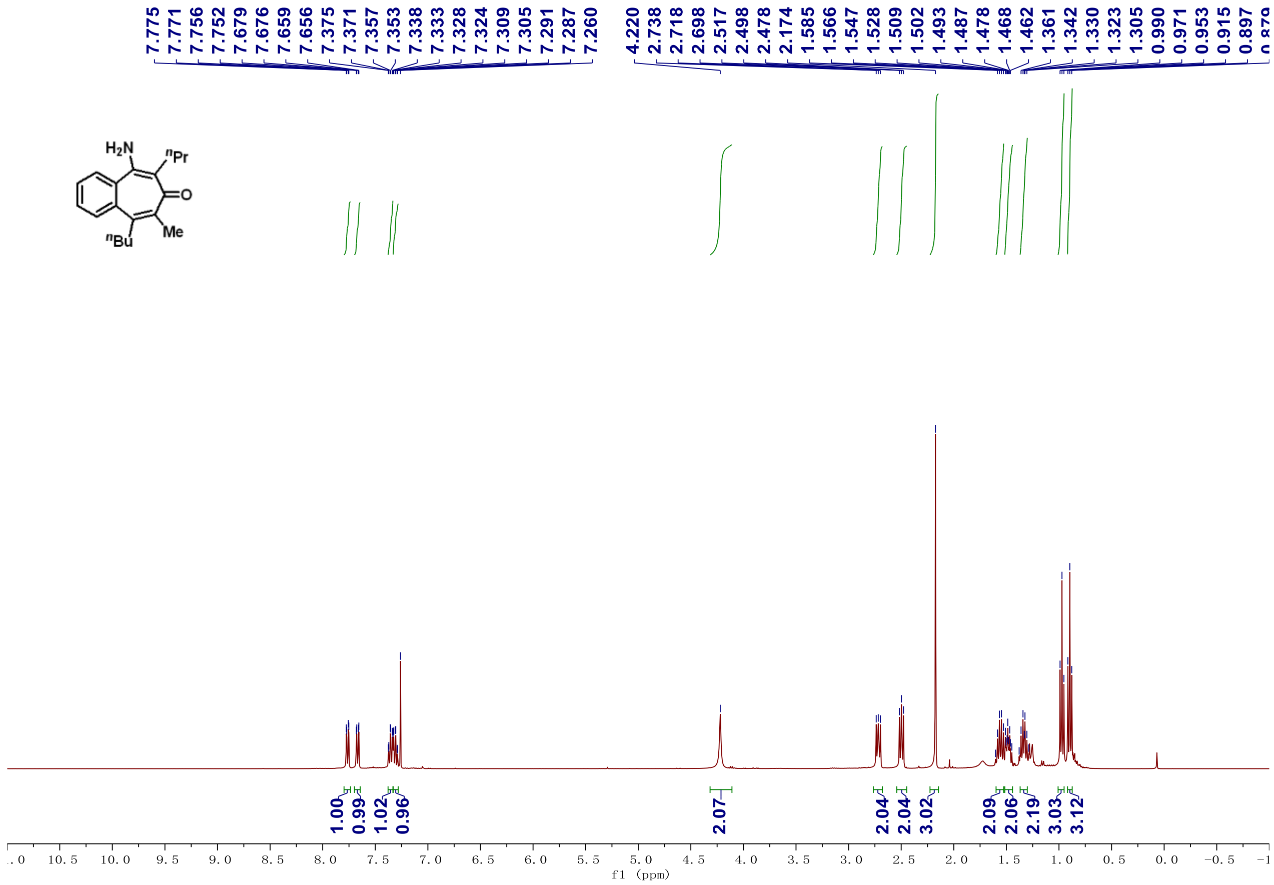

^{13}**C NMR**-spectrum (100 MHz, $CDCl_3$) of **3x**

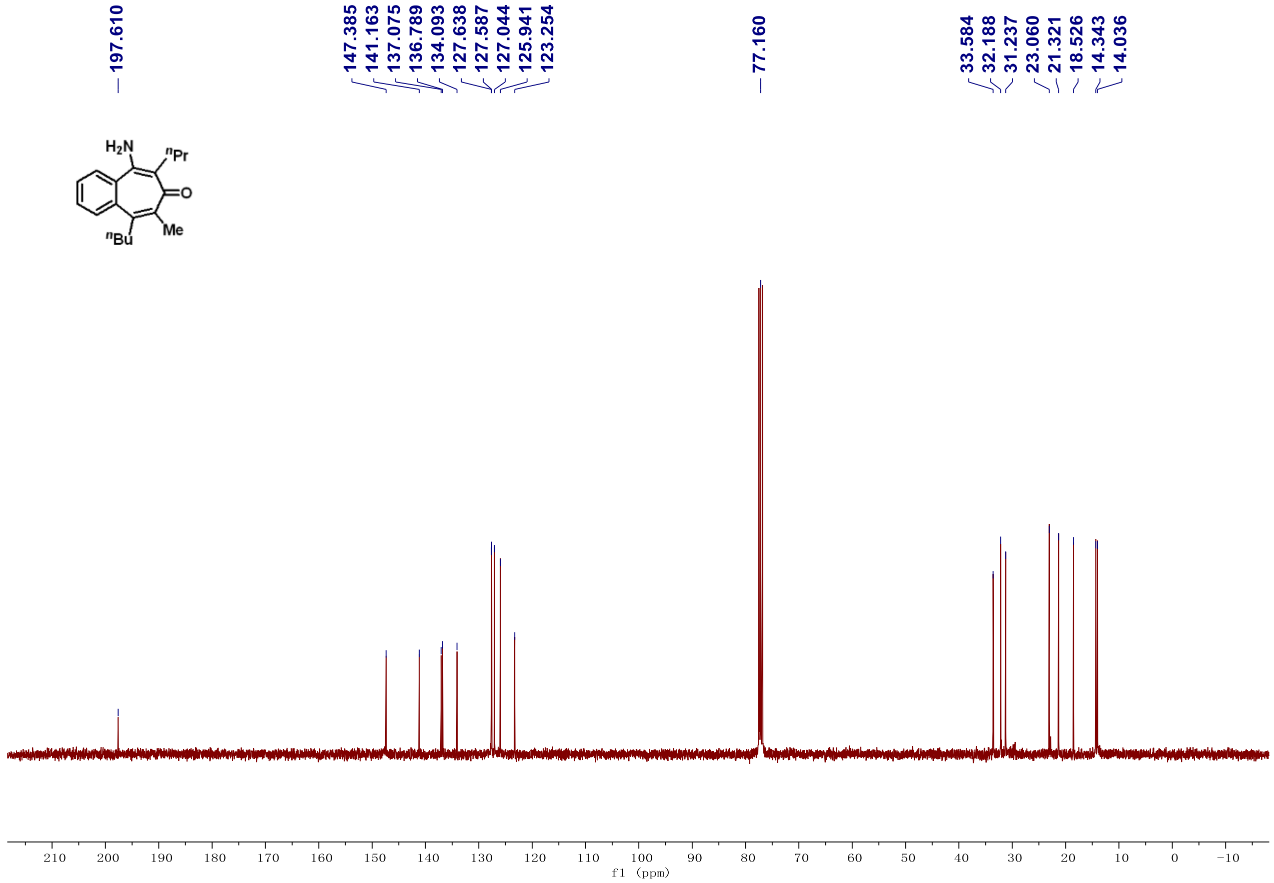

^{1}H NMR-spectrum (400 MHz, $CDCl_3$) of **5**

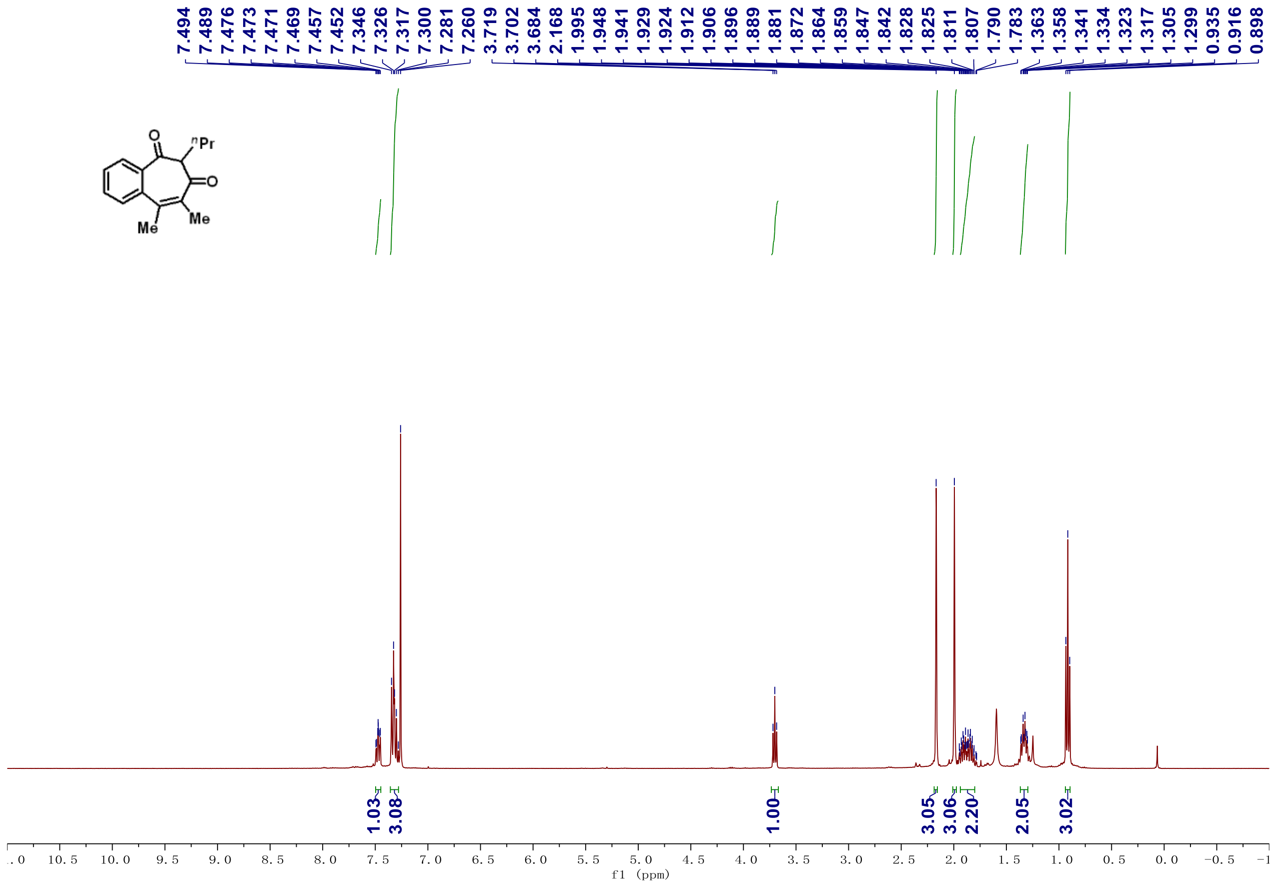

^{13}C NMR-spectrum (100 MHz, $CDCl_3$) of **5**

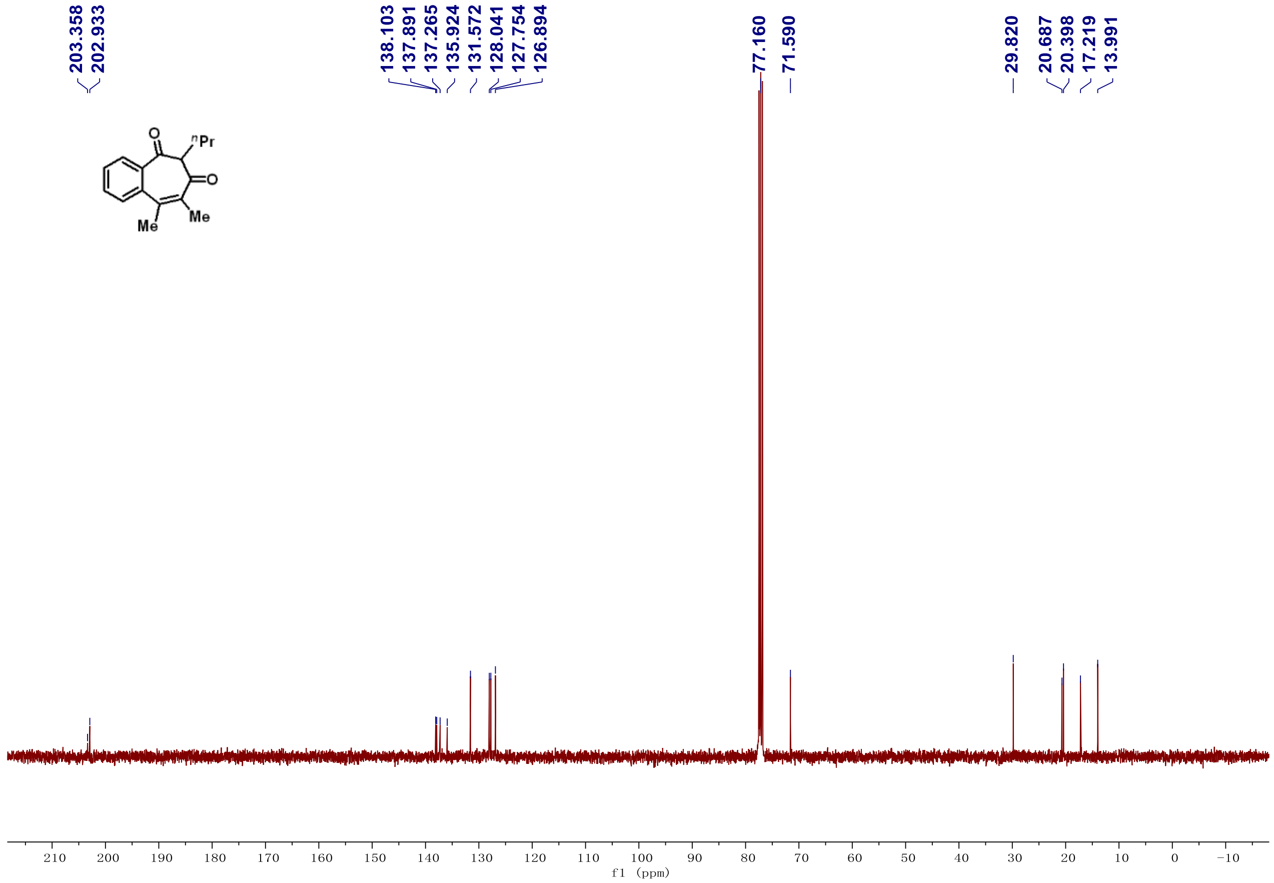

^{1}H NMR-spectrum (400 MHz, $CDCl_3$) of **6**

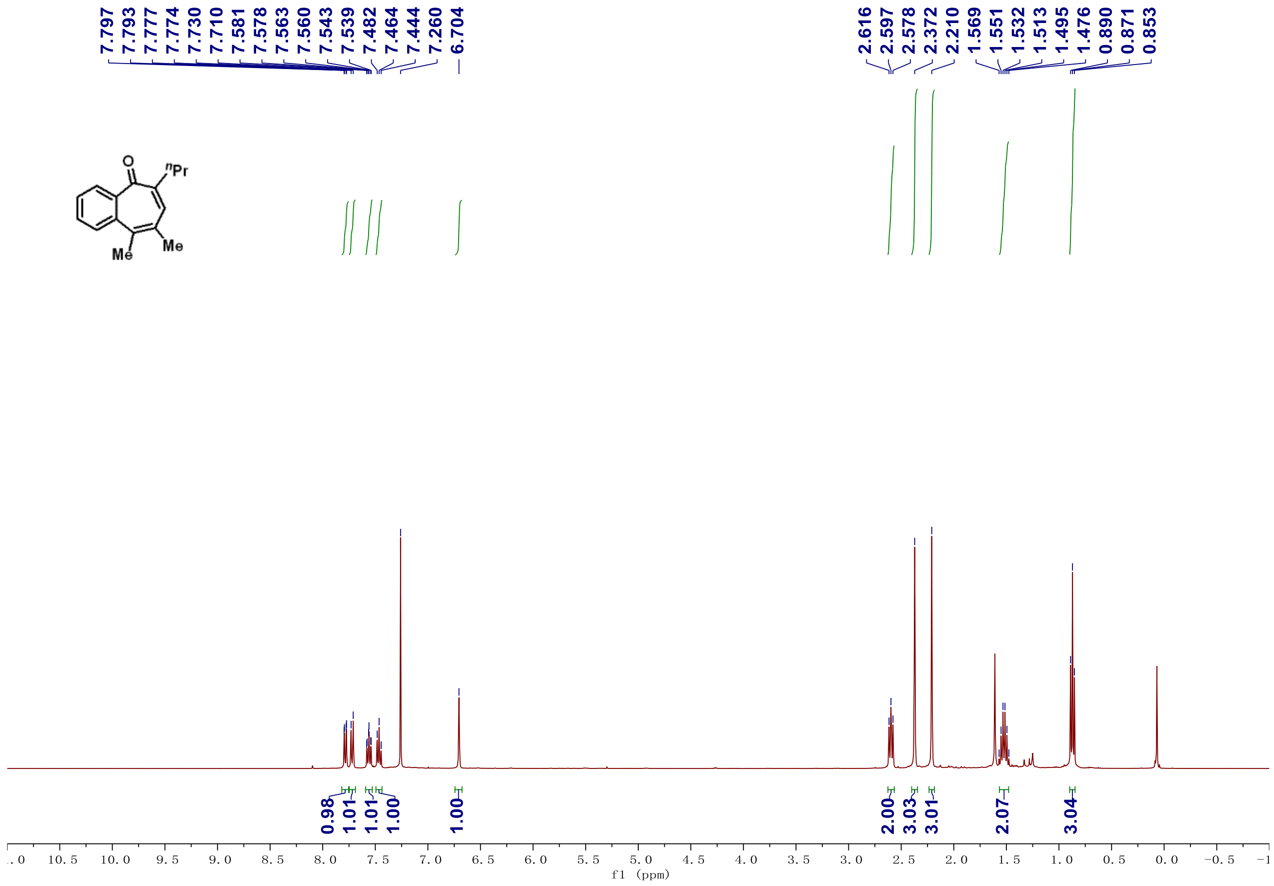

^{13}C NMR-spectrum (100 MHz, $CDCl_3$) of **6**

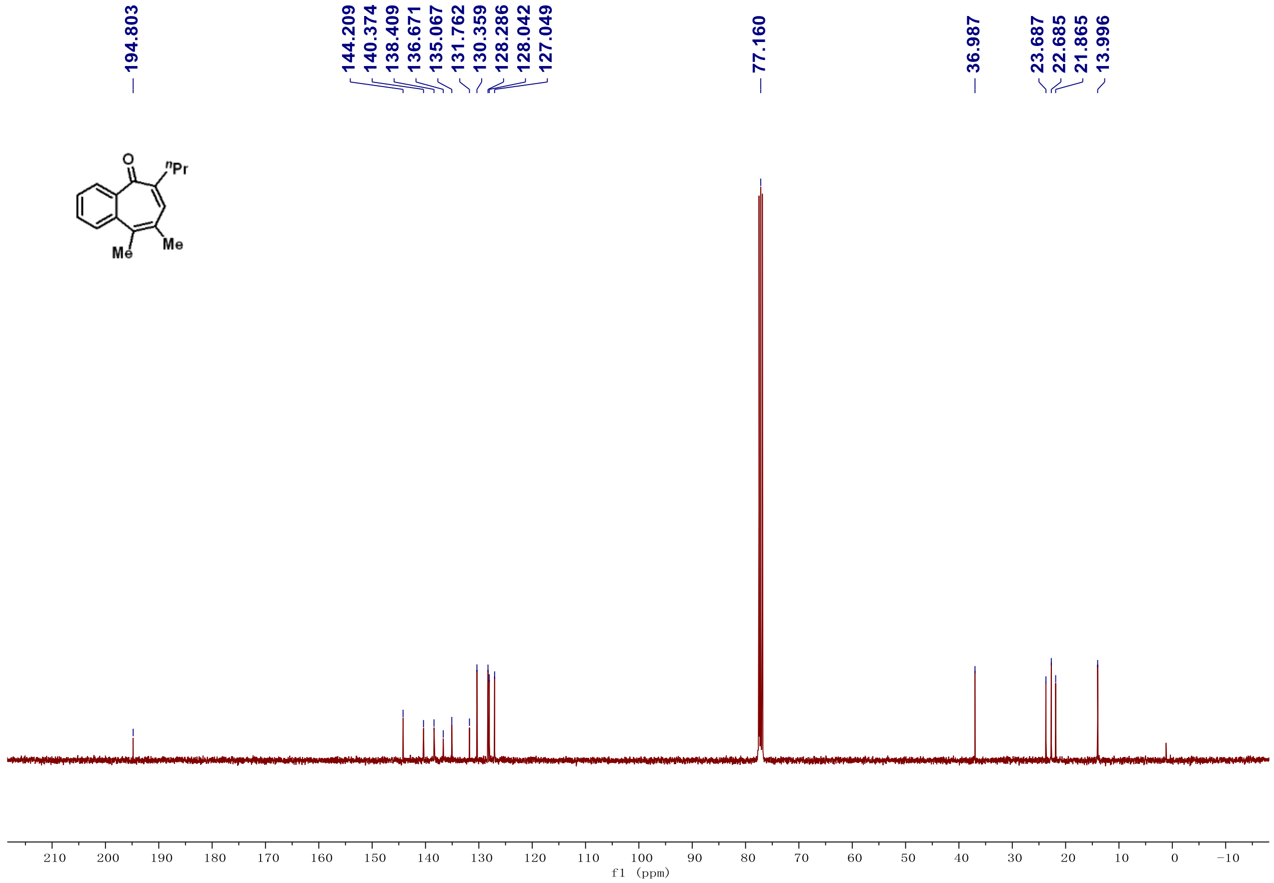

^{1}H NMR-spectrum (400 MHz, $CDCl_3$) of **7**

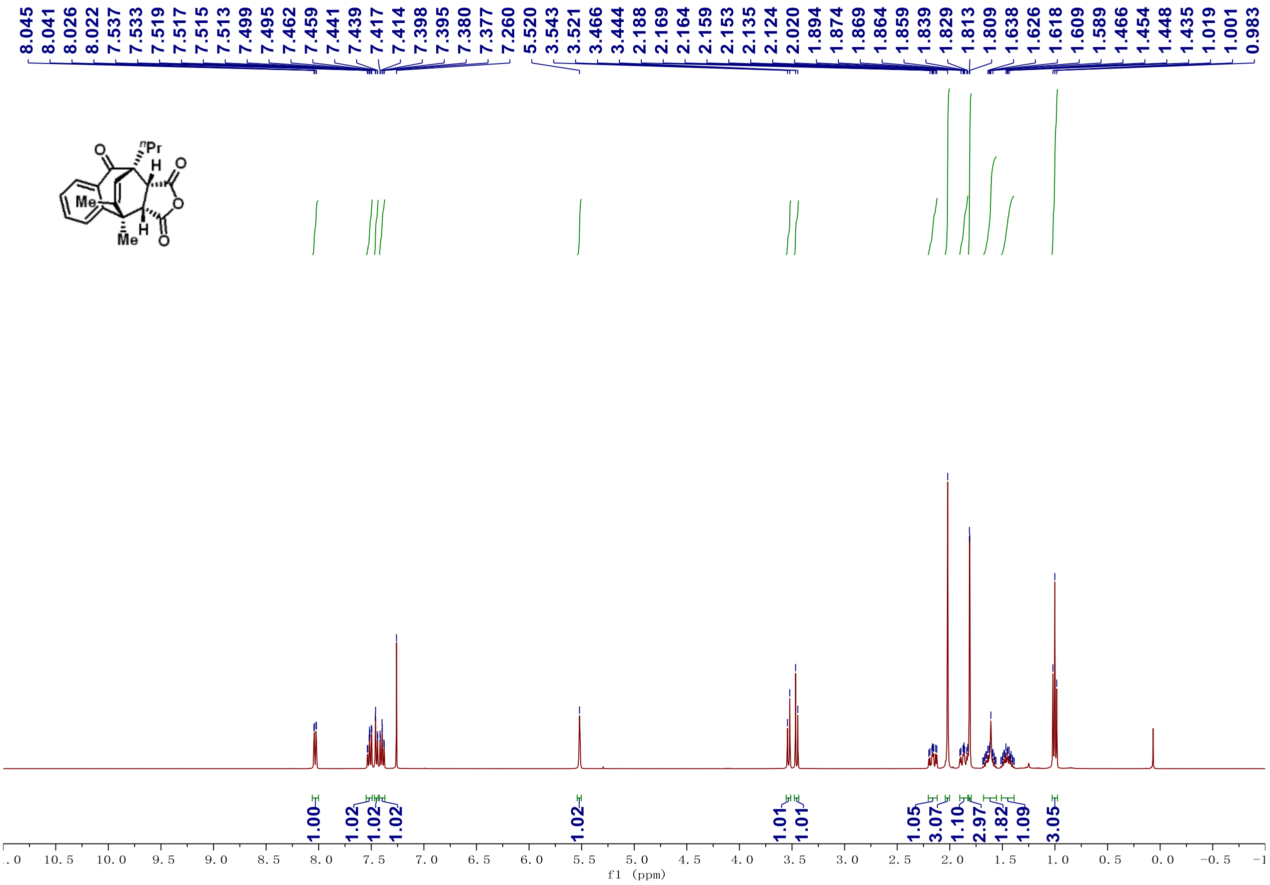

^{13}C NMR-spectrum (100 MHz, $CDCl_3$) of **7**

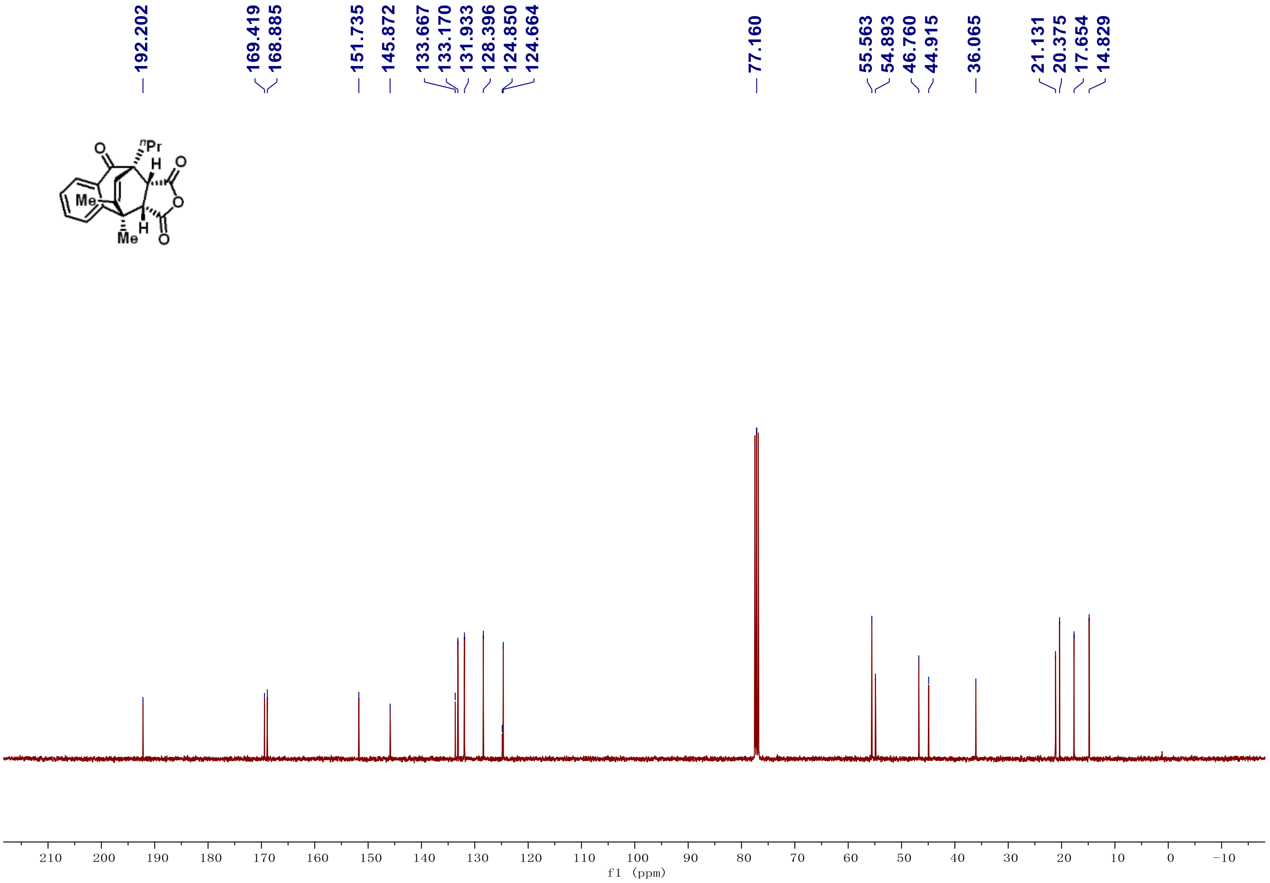

^{1}H NMR-spectrum (400 MHz, $CDCl_3$) of **8**

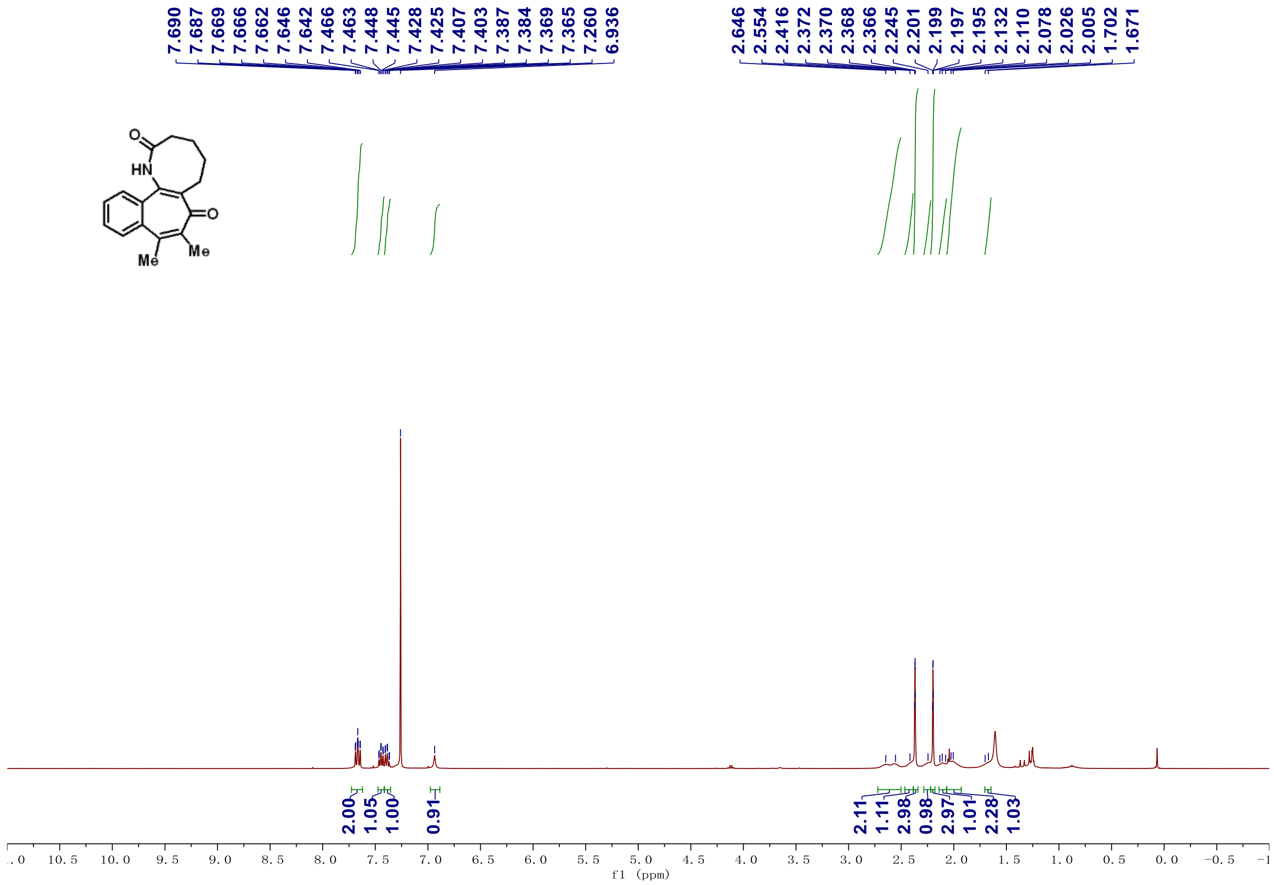

^{13}C NMR-spectrum (100 MHz, $CDCl_3$) of **8**

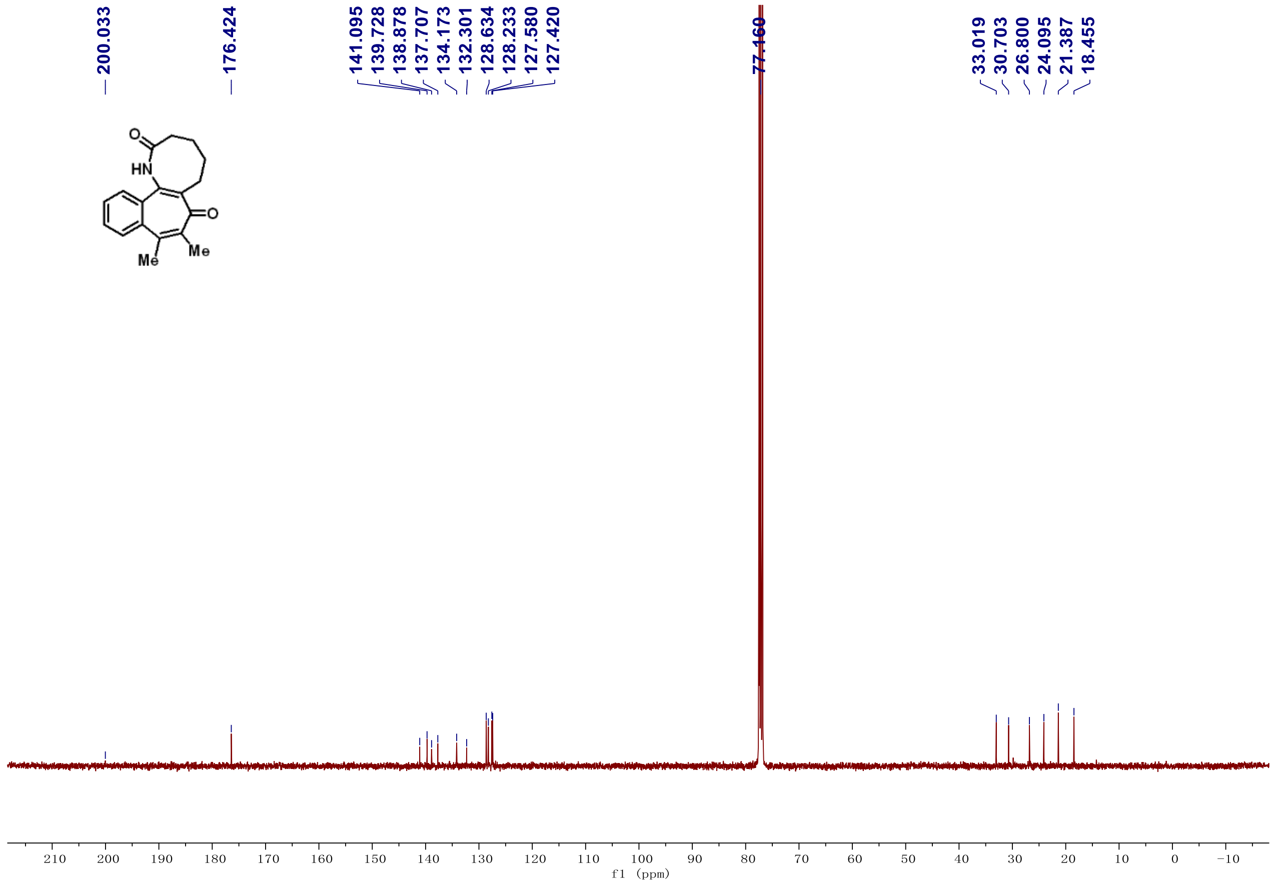

^{1}H NMR-spectrum (400 MHz, $CDCl_3$) of **9**

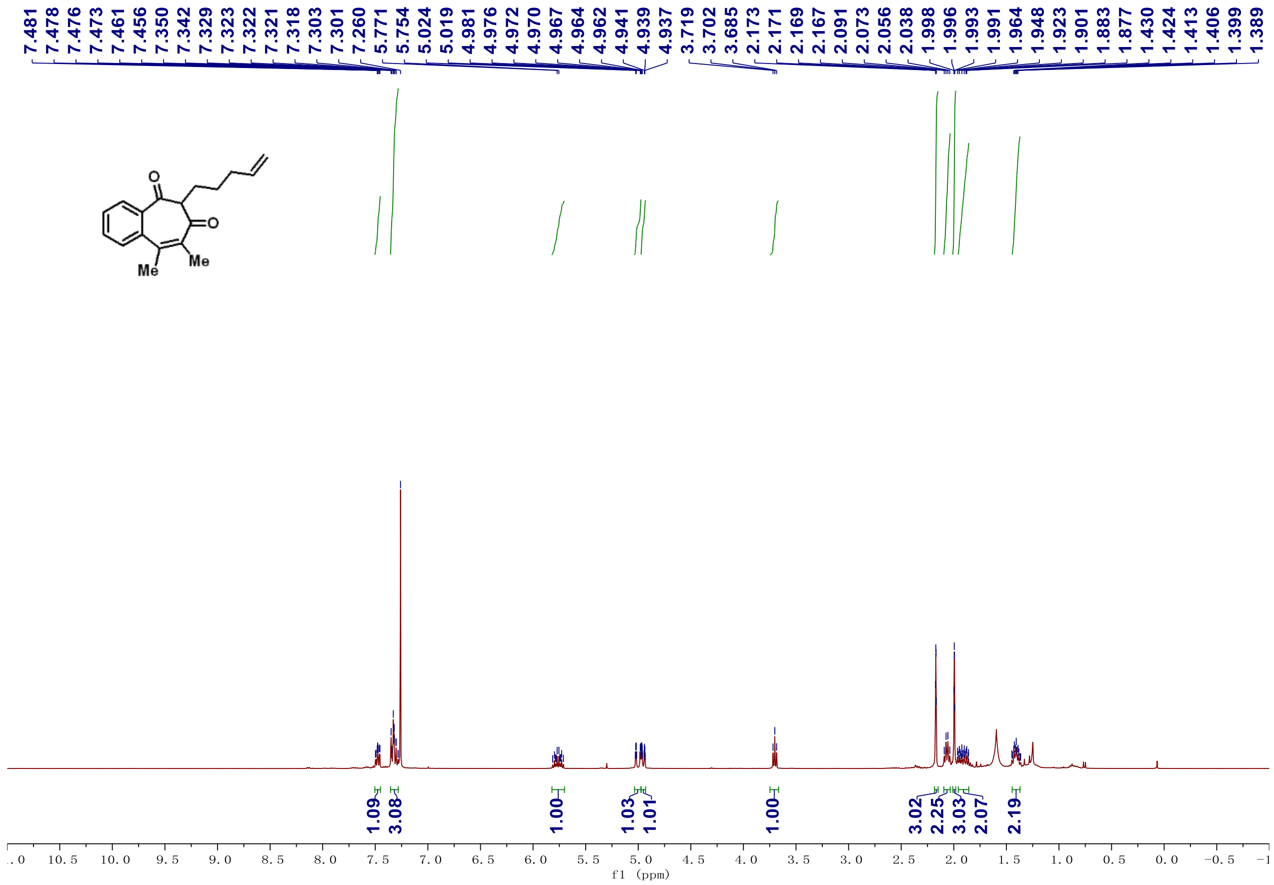

^{13}C NMR-spectrum (100 MHz, $CDCl_3$) of **9**

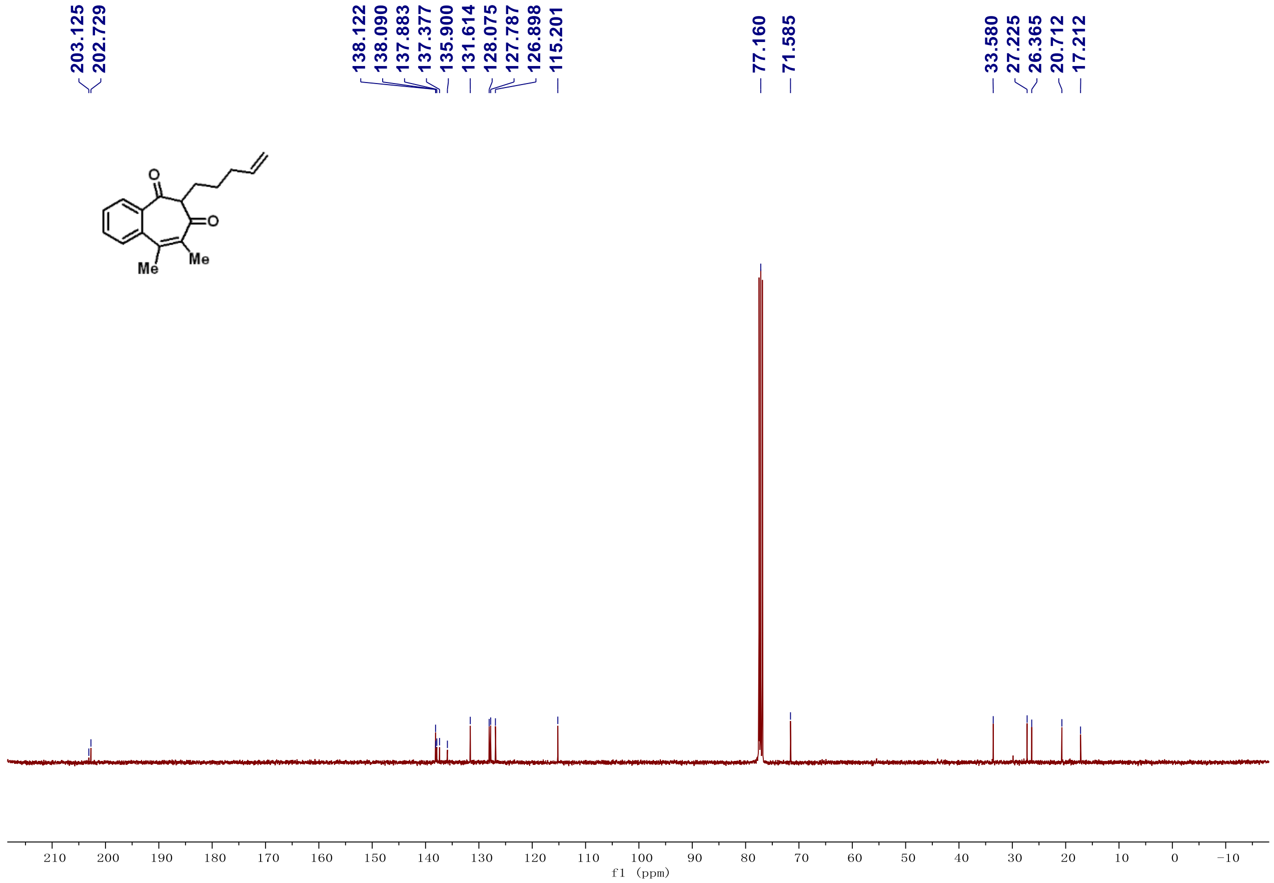

^{1}H NMR-spectrum (400 MHz, $CDCl_3$) of **10**

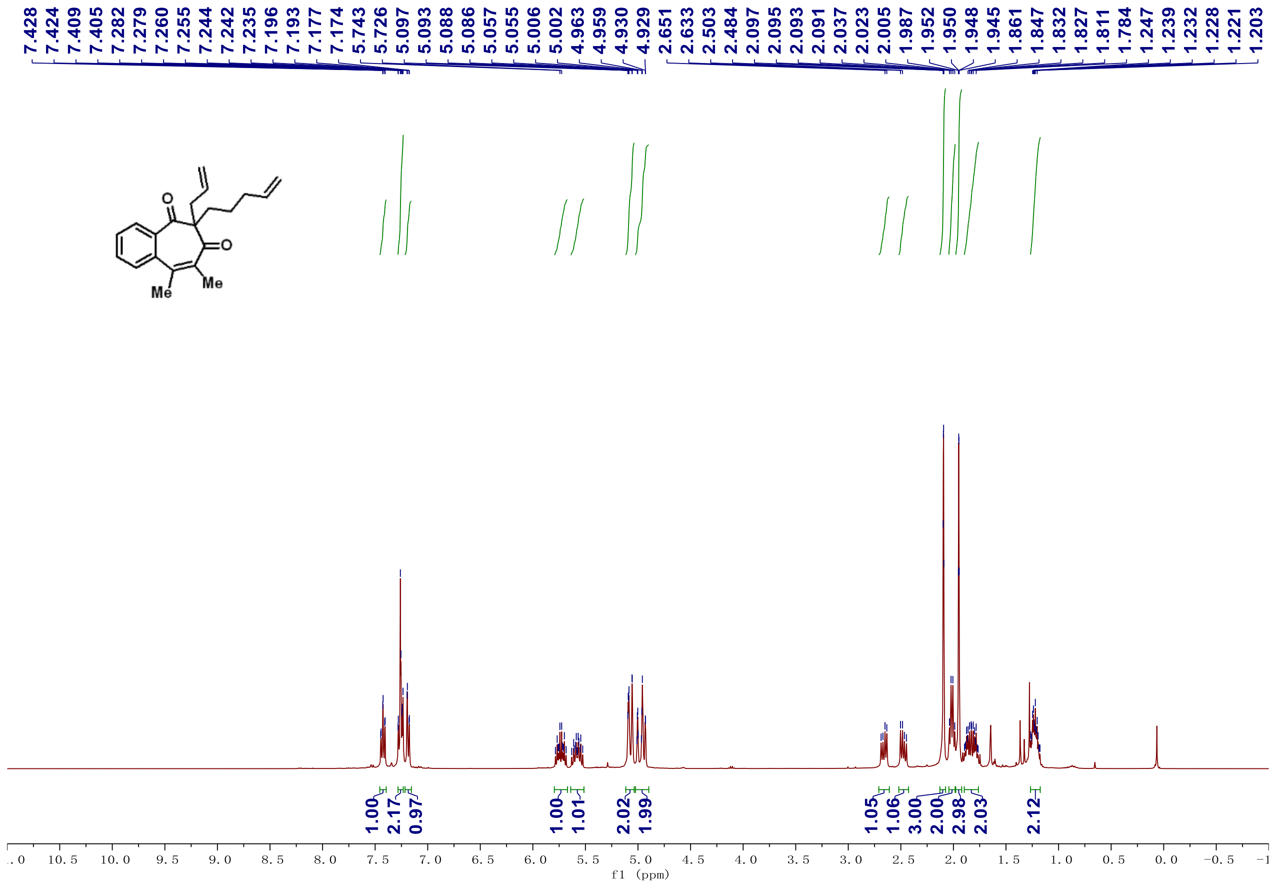

^{13}C NMR-spectrum (100 MHz, $CDCl_3$) of **10**

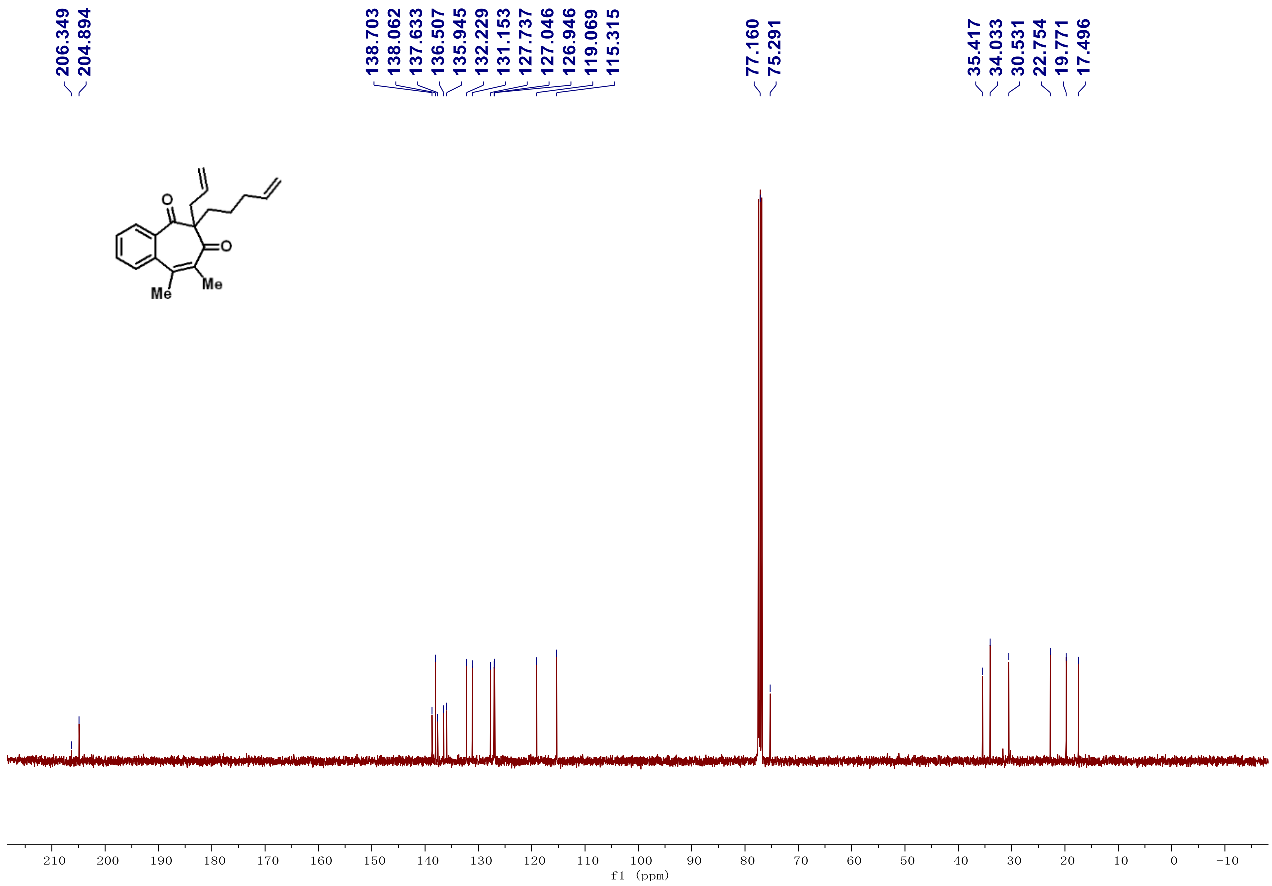

^{1}**H NMR**-spectrum (400 MHz, $CDCl_3$) of **11**

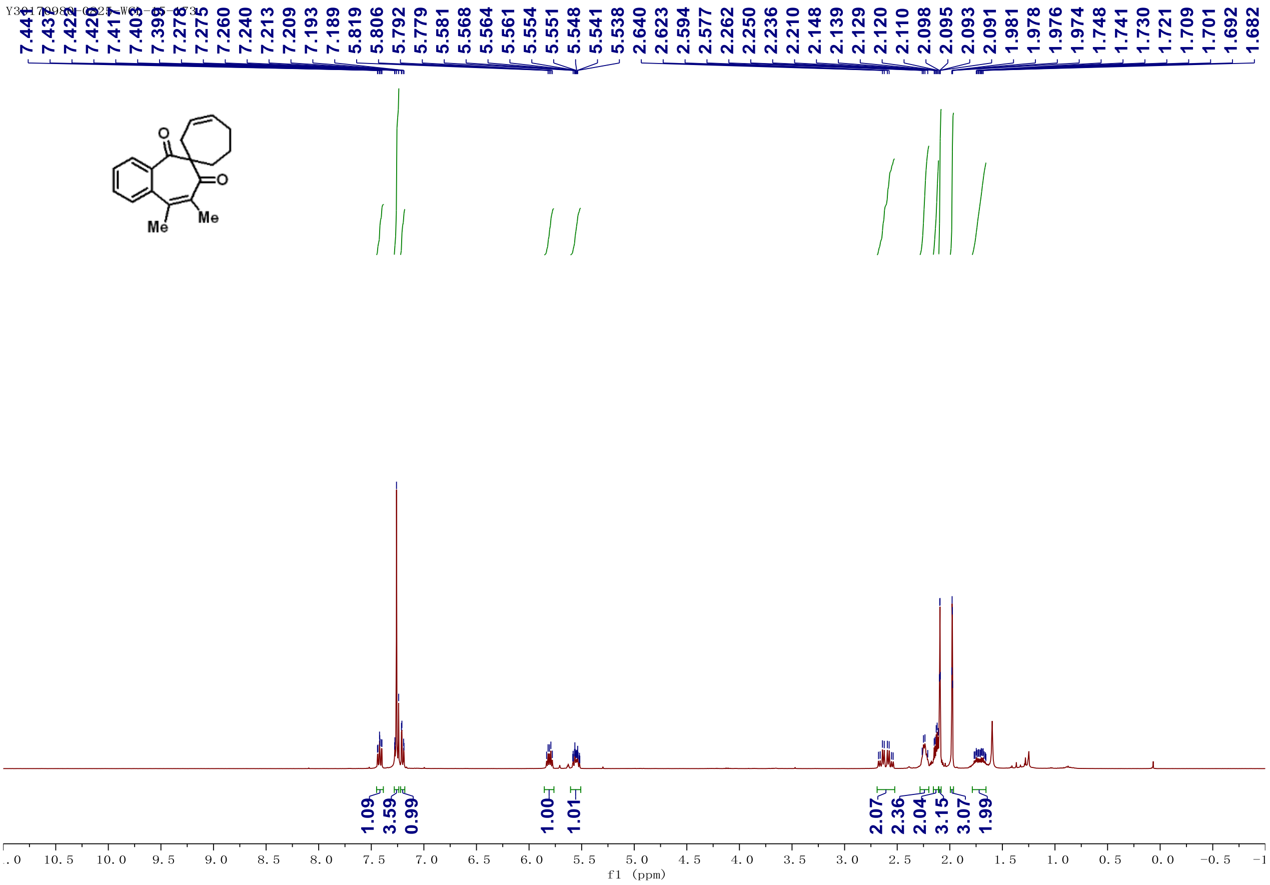

^{13}**C NMR**-spectrum (100 MHz, $CDCl_3$) of **11**

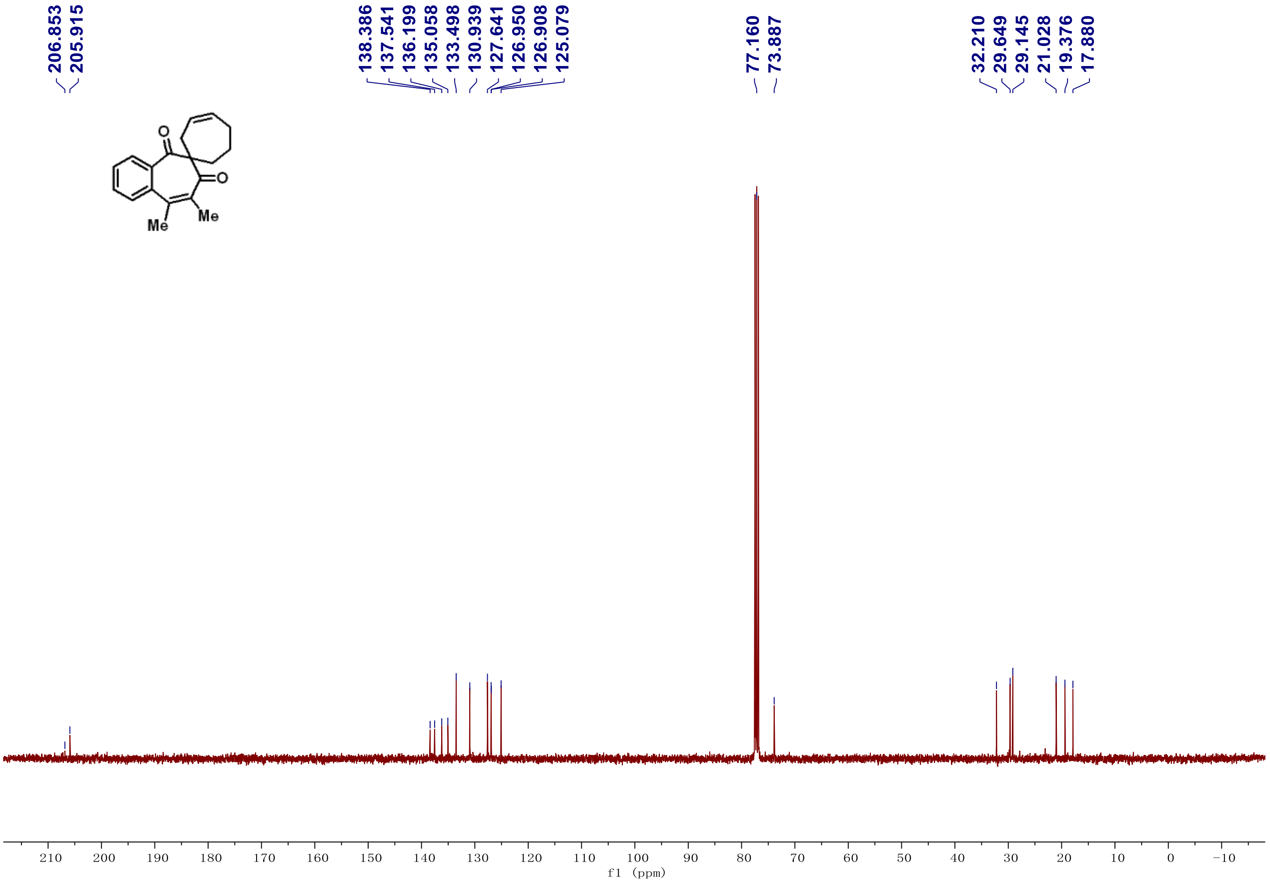

^{1}H NMR-spectrum (400 MHz, $CDCl_3$) of **4a**

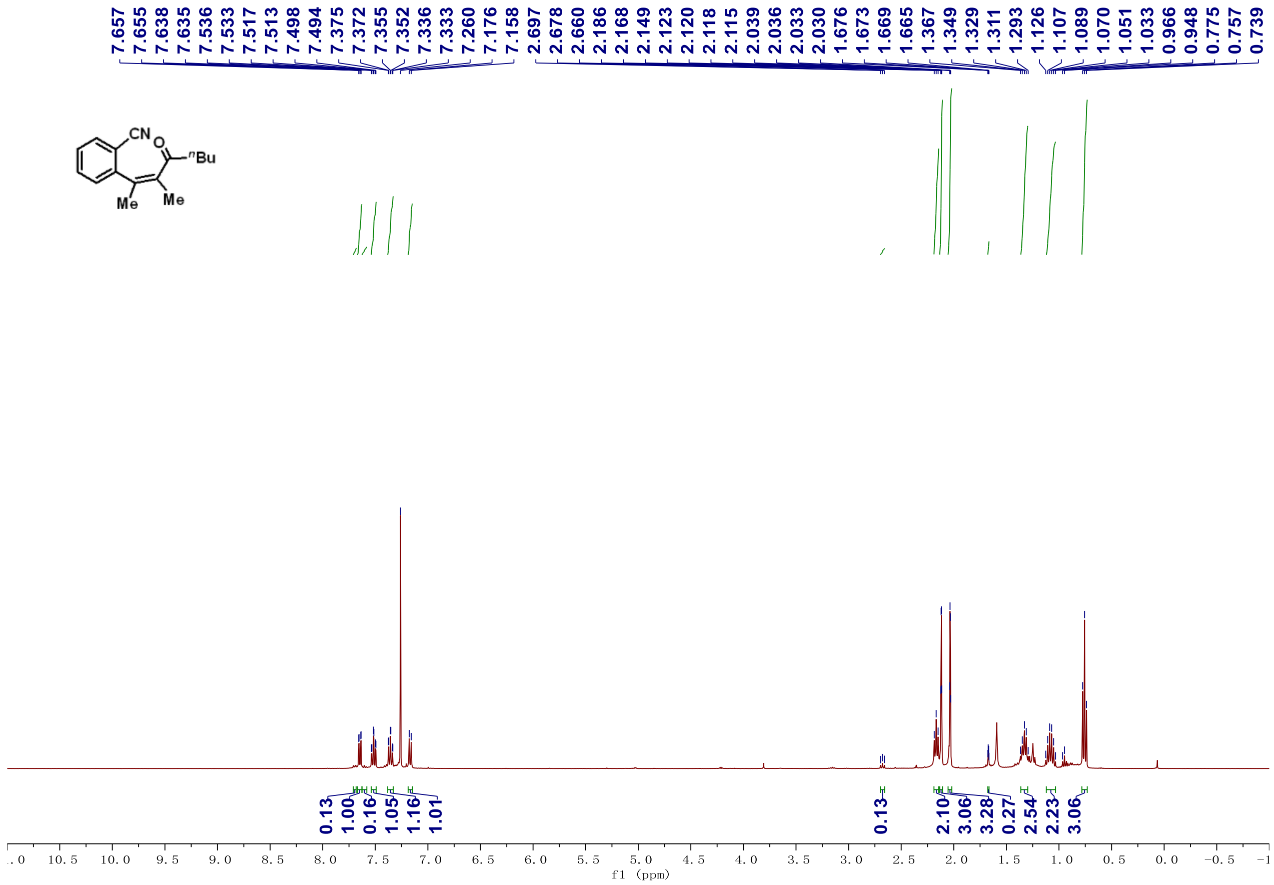

^{13}C NMR-spectrum (100 MHz, $CDCl_3$) of **4a**

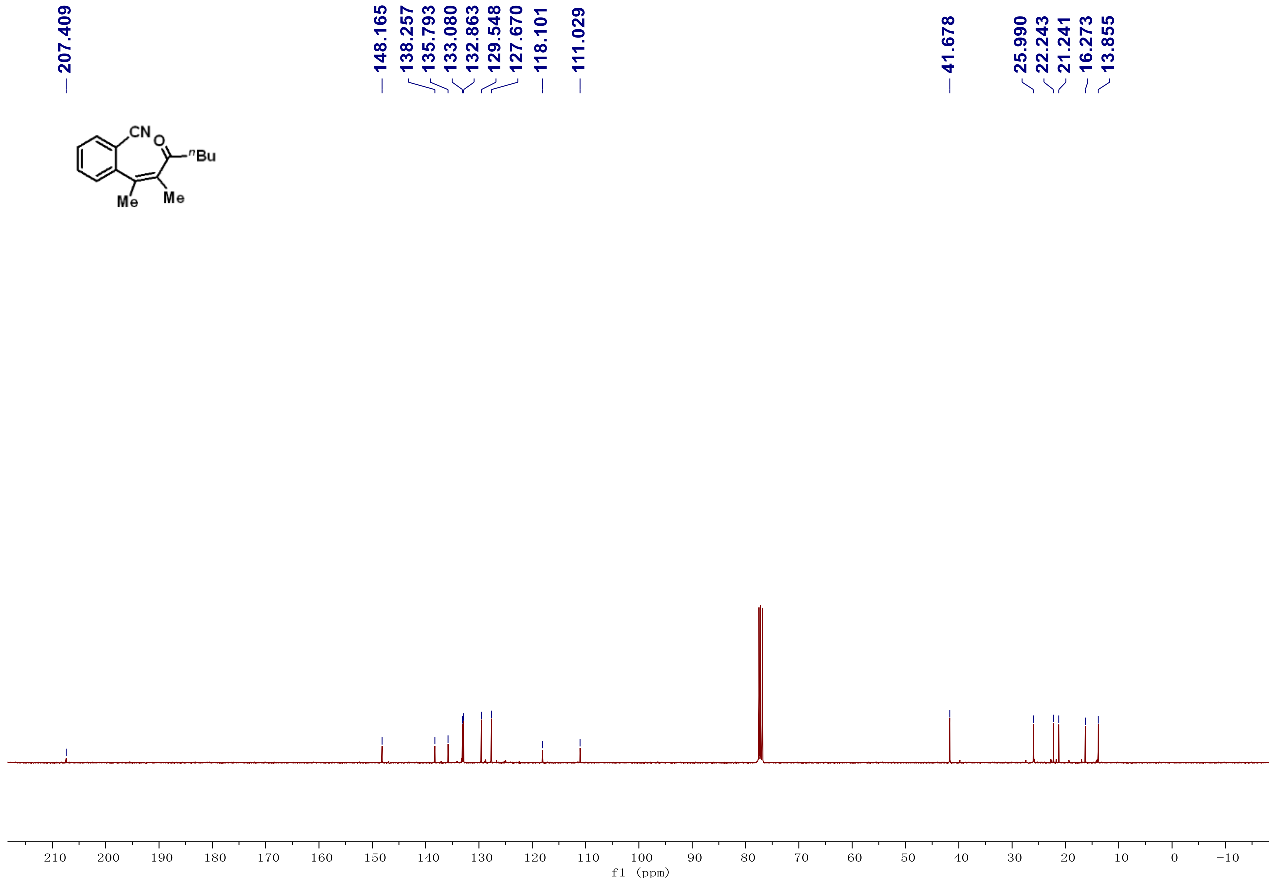

References

1. Iwata, T., Inagaki, F. & Mukai, C. *Angew. Chem. Int. Ed.* **52***,* 11138 (2013).
2. Krasovskiy, A. & Knochel, P. *Synthesis* **5**, 0890 (2006).
3. Calvin, J. R., Frederick, M. O., Laird, D. L. T., Remacle, J. R. & May, S. A. *Org. Lett*. **14**, 1038 (2012).
